# Estimating the impact of HIV PrEP regimens containing long-acting injectable cabotegravir or daily oral tenofovir disoproxil fumarate/emtricitabine among men who have sex with men in the United States: a mathematical modelling study for HPTN 083

**DOI:** 10.1016/j.lana.2022.100416

**Published:** 2023-01-17

**Authors:** Kate M. Mitchell, Marie-Claude Boily, Brett Hanscom, Mia Moore, Jeffery Todd, Gabriela Paz-Bailey, Cyprian Wejnert, Albert Liu, Deborah J. Donnell, Beatriz Grinsztejn, Raphael J. Landovitz, Dobromir T. Dimitrov

**Affiliations:** a MRC Centre for Global Infectious Disease Analysis, School of Public Health, Imperial College London, London, United Kingdom; b HIV Prevention Trials Network Modelling Centre, Imperial College London, London, United Kingdom; c Vaccine and Infectious Disease Division, Fred Hutchinson Cancer Center, Seattle, WA, USA; d Centers for Disease Control and Prevention, Atlanta, GA, USA; e Division of Vector-Borne Diseases, Centers for Disease Control and Prevention, San Juan, Puerto Rico; f Bridge HIV, Population Health Division, San Francisco Department of Public Health, San Francisco, CA, USA; g Department of Medicine, University of California San Francisco, San Francisco, CA, USA; h Instituto Nacional de Infectologia Evandro Chagas, Fundação Oswaldo Cruz, Rio de Janeiro, Brazil; i Center for Clinical AIDS Research and Education, University of California Los Angeles, Los Angeles, CA, USA; j Division of Infectious Diseases, David Geffen School of Medicine at UCLA, Los Angeles, CA, USA

**Keywords:** HIV, Pre-exposure prophylaxis, Long-acting PrEP, Injectable PrEP, Cabotegravir, Men who have sex with men, MSM, United States, Atlanta, Mathematical modelling

## Abstract

**Background:**

The HPTN 083 trial demonstrated superiority of HIV pre-exposure prophylaxis (PrEP) containing long-acting injectable cabotegravir (CAB) to daily oral tenofovir disoproxil fumarate/emtricitabine (TDF/FTC) among men who have sex with men (MSM). We compared the potential population-level impact of TDF/FTC and CAB among MSM in Atlanta, Georgia.

**Methods:**

An MSM HIV transmission model was calibrated to Atlanta-specific data on HIV prevalence and PrEP usage (percentage of uninfected MSM on PrEP), assuming only PrEP-indicated MSM used PrEP. CAB effectiveness (efficacy × adherence) of 91% was estimated using data from HPTN 083 and previous TDF/FTC trials. We estimated HIV infections averted over 5/10 years if TDF/FTC use were maintained, or if all TDF/FTC users switched to CAB in January 2022 (vs. no PrEP or continued TDF/FTC use). CAB scenarios with 10%/20% more users were also considered. Progress towards Ending the HIV Epidemic (EHE) goals (75%/90% fewer HIV infections in 2025/2030 vs. 2017) was estimated.

**Findings:**

We predicted TDF/FTC at current usage (∼28%) would avert 36.3% of new HIV infections (95% credible interval 25.6–48.7%) among all Atlanta MSM over 2022–2026 vs. no PrEP. Switching to CAB with similar usage may prevent 44.6% (33.2–56.6%) infections vs. no PrEP and 11.9% (5.2–20.2%) infections vs. continued TDF/FTC. Increasing CAB usage 20% could increase the incremental impact over TDF/FTC to 30.0% over 2022–2026, getting ∼60% towards reaching EHE goals (47%/54% fewer infections in 2025/2030). Reaching the 2030 EHE goal would require 93% CAB usage.

**Interpretation:**

If CAB effectiveness were like HPTN 083, CAB could prevent more infections than TDF/FTC at similar usage. Increased CAB usage could contribute substantially towards reaching EHE goals, but the usage required to meet EHE goals is unrealistic.

**Funding:**

10.13039/100000002NIH, 10.13039/501100000265MRC.

**Research in context**

**Evidence before this study**

The HPTN 083 trial demonstrated superiority of HIV pre-exposure prophylaxis (PrEP) containing long-acting injectable cabotegravir over daily oral tenofovir disoproxil fumarate/emtricitabine among men who have sex with men (MSM). The population-level impact of long-acting injectable PrEP for MSM is not yet known.

We searched PubMed for articles estimating the population-level impact of long-acting injectable PrEP compared with daily oral PrEP among MSM published 1st January 2012–30th April 2022, with no language restrictions, using the following search terms: (“long-acting” OR “injectable”) AND (PrEP OR “pre-exposure prophylaxis”) AND (HIV OR AIDS) AND (“men who have sex with men” OR MSM). We identified three studies that had estimated the impact of long-acting injectable PrEP among MSM, all of which estimated the impact of long-acting injectable cabotegravir for MSM in the United States. Two of these studies were conducted before human trial data were available and used data from macaque studies to estimate cabotegravir efficacy. Both studies used dynamic HIV transmission models to estimate the impact of long-acting injectable vs. daily oral PrEP among MSM in Atlanta, and both found similar impact: with 30% of uninfected MSM using PrEP, switching all PrEP users from daily oral to long-acting injectable PrEP would prevent around 13% of new HIV infections over 10 years. Both studies also found that a larger impact would be achieved if the availability of long-acting injectable PrEP led to a greater number of MSM using PrEP. The third study used a cohort model without onwards HIV transmission to estimate the cost-effectiveness of long-acting injectable cabotegravir compared with daily oral PrEP among MSM in the United States, using data from the HPTN 083 trial. This modelling suggested that switching MSM from daily oral to long-acting injectable PrEP would avert around 12% of HIV infections over 10 years but did not estimate the impact of preventing secondary infections.

**Added value of this study**

We used a dynamic HIV transmission model, parameterised with long-acting injectable PrEP effectiveness estimates from the HPTN 083 trial, to estimate the population-level impact (including prevention of secondary infections) of long-acting injectable PrEP on new HIV infections among MSM in Atlanta. With this transmission model and up-to-date PrEP effectiveness information, we also found that switching US MSM from daily oral to long-acting injectable PrEP would avert around 13% of new HIV infections over 10 years and predicted greater impact if the availability of long-acting injectable PrEP led to more MSM using PrEP. In addition, we estimated that increased use of long-acting injectable PrEP among MSM could contribute substantially towards reaching US Ending the HIV Epidemic goals but is unlikely to be sufficient to achieve them.

**Implications of all the available evidence**

Long-acting injectable cabotegravir could have a substantial impact on the HIV epidemic among MSM in the US, especially if it is used by MSM who are not already using daily oral PrEP. Other measures, such as improvements in diagnosis and viral suppression, will also likely be needed to meet national goals for Ending the HIV Epidemic.

## Introduction

Men who have sex with men (MSM) in the United States (US) continue to experience high rates of HIV infection. An estimated 23,100 MSM were newly infected with HIV nationally in 2019, only a small (9%) decrease since 2015.^1^ Higher HIV incidence is seen among younger and among Black and Hispanic MSM, and MSM living in the Southern US.^1^ Atlanta, Georgia, in the Southern US, is one of the US cities with the highest HIV prevalence among MSM, 33% in 2017.^2^ The Ending the HIV Epidemic (EHE) initiative, announced in 2019, seeks to reduce the number of new HIV infections in the US by 75% by 2025, and by at least 90% by 2030, through improving levels of HIV diagnosis, antiretroviral therapy (ART) usage and viral suppression, and prevention including pre-exposure prophylaxis (PrEP).^3^ Atlanta is included in the priority jurisdictions which are the initial focus for EHE interventions.^3^

HIV PrEP involves people who are not infected with HIV but are at high risk of HIV acquisition using antiretrovirals as prophylaxis to reduce their HIV acquisition risk. Oral PrEP using tenofovir disoproxil fumarate/emtricitabine (TDF/FTC) has been shown to greatly reduce the risk of HIV acquisition when taken daily by MSM.^4^ For MSM, taking four or more oral doses of TDF/FTC per week is also associated with high protection.^5^ However, PrEP initiation, continuation, and adherence by US MSM has been variable, with lower levels of initiation, continuation, and adherence seen among younger MSM, Black MSM and MSM living in the South,^6^, ^7^, ^8^ despite these populations having higher HIV exposure. In addition to more effective ways of supporting adherence to oral PrEP, there is also a need for alternative PrEP delivery modes, including those not relying on daily dosing.

The HIV Prevention Trials Network (HPTN) 083 trial investigated the safety and efficacy of a regimen containing long-acting injectable cabotegravir (CAB) compared with daily oral TDF/FTC for HIV prevention among MSM and transgender women. The CAB regimen included a month-long lead-in phase of daily oral cabotegravir followed by long-acting cabotegravir injections administered every 8 weeks. The CAB regimen was shown to be superior to TDF/FTC for HIV prevention, resulting in a 66% (95% confidence interval 38–82%) reduction in HIV incidence,^9^ suggesting that CAB overcomes some of the adherence problems associated with daily TDF/FTC.

Preference studies suggest that 31–67% of US MSM who currently or previously used daily oral PrEP are interested in switching to injectable PrEP,^10^^,^^11^ and that 25–47% of MSM not using PrEP would prefer an injectable PrEP option.^12^^,^^13^

Two dynamic transmission modelling studies among US MSM (both in Atlanta, Georgia) have provided theoretical estimates of population-level CAB impact^14^^,^^15^ using data from macaque studies to estimate CAB efficacy, as human trial data were not yet available. One study used an individual-based model,^14^ the other a dynamic network model.^15^ Both studies estimated CAB efficacy from the same macaque study. Both models suggested similar impact of CAB: with 30% of uninfected MSM using PrEP, switching all PrEP users from TDF/FTC to CAB would prevent around 13% of new HIV infections over 10 years.

We used mathematical modelling, together with initial estimates of CAB efficacy (vs. no PrEP) from the HPTN 083 trial, to assess the potential population-level impact on new HIV infections among MSM in Atlanta of (a) current TDF/FTC users continuing on TDF/FTC (compared with no PrEP use), (b) current TDF/FTC users switching to CAB (compared with no PrEP use or with continued TDF/FTC use), or (c) current TDF/FTC users switching to CAB with additional new recruits starting CAB (increasing the total number of PrEP users by 10% or 20%; vs. no PrEP or continued TDF/FTC). We also assessed the potential role of these PrEP scenarios in achieving the EHE goals.

## Methods

### Mathematical model structure

We extended a previous mathematical model of sexual HIV transmission and treatment among MSM^16^ to include PrEP use and stratification by PrEP indication (based on 2017 US guidelines).^17^

The model is a deterministic, compartmental model of HIV transmission among sexually active MSM. The modelled MSM population is stratified by age, race, PrEP indication, HIV infection status, PrEP use, and (for those living with HIV) set-point viral load, HIV infection stage by CD4 T-cell count, and engagement with the HIV care continuum.

A proportion *p* of MSM in the model never undergo routine HIV testing, only testing for HIV if they develop AIDS symptoms (following infection and progression to AIDS). The remainder (1 − *p*) undergo routine HIV testing. They may be offered PrEP (at a rate proportional to their testing rate) and may accept PrEP. Those on PrEP may become infected with HIV, at a lower rate than if they were not on PrEP. Modelled MSM on PrEP test regularly for HIV and may drop out of PrEP into the equivalent uninfected/undiagnosed compartments. PrEP is introduced in the model in 2012, with all PrEP use assumed to be TDF/FTC until the end of 2021. In the main analysis, only MSM in the PrEP-indicated group ever use PrEP. Further model details are given in the Supplementary Material.

### Data sources

The model was parameterised using demographic, sexual risk behaviour, and intervention behaviour data for MSM in Atlanta where possible, and for MSM elsewhere in the US otherwise, and using data on HIV disease progression, infection probabilities, and intervention efficacy from the published literature, where possible from MSM populations (key parameters in Table 1, all parameters in Table S1).

**Table 1:** Summary of key sexual behaviour and intervention parameters in model fits, and data ranges used for model fitting.

Parameters	Range	Source
Sexual behaviour parameters (2021)		
Average number of new anal sex partners per year		Estimated from Atlanta NHBS data
18- to 24-year-old Black MSM – not PrEP indicated	0.88–2.06	
>24-year-old Black MSM – not PrEP indicated	1.07–1.69	
18- to 24-year-old White MSM – not PrEP indicated	0.93–3.55	
>24-year-old White MSM – not PrEP indicated	1.13–3.29	
18- to 24-year-old Black MSM – PrEP indicated	2.46–4.28	
>24-year-old Black MSM – PrEP indicated	3.63–7.06	
18- to 24-year-old White MSM – PrEP indicated	0.44–2.00	
>24-year-old White MSM – PrEP indicated	4.69–18.3	
Percentage of sex acts in which condom used		Atlanta NHBS
Main partnerships, both partners Black – not PrEP indicated	42–69	
Main partnerships, both partners Black – PrEP indicated	18–56	
Main partnerships, either partner White – not PrEP indicated	21–56	
Main partnerships, either partner White – PrEP indicated	3–31	
Casual/commercial partnerships (any race) – not PrEP indicated	41–91	
Casual/commercial partnerships (any race) – PrEP indicated	18–59	
HIV testing parameters (2021)		
Percentage of undiagnosed MSM (not on PrEP) testing for HIV per year		Relative levels from Atlanta NHBS, reduced to fit to diagnosis levels
18-to 24-year-old Black MSM	29–47	
>24-year-old Black MSM	25–42	
18- to 24-year-old White MSM	13–45	
>24-year-old White MSM	25–42	
PrEP parameters (2021)		
Percentage of those accepting PrEP when offered		US PrEP Demo project
18- to 24-year-old Black MSM	42–63	
>24-year-old Black MSM	41–64	
18- to 24-year-old White MSM	65–73	
>24-year-old White MSM	65–74	
Percentage on TDF/FTC PrEP taking ≥4 doses/week [i.e. adherence]	Main (sensitivity analysis)	US PrEP Demo project (US MSM in HPTN 083 trial)
18- to 24-year-old Black MSM	48–78 (58–64)	
>24-year-old Black MSM	45–63 (58–64)	
18- to 24-year-old White MSM	87–96 (75–81)	
>24-year-old White MSM	88–93 (75–81)	
Percentage on PrEP dropping out each year		US PrEP Demo project
18- to 24-year-old Black MSM	26–55	
>24-year-old Black MSM	28–55	
18- to 24-year-old White MSM	4–30	
>24-year-old White MSM	15–23	
Intervention efficacies		
Reduction in HIV acquisition risk due to correct condom use (%)	58–79	US MSM estimate
Reduction in HIV transmission risk due to HIV-infected partner being on ART and fully suppressed (%)	100 (fixed)	European MSM estimate
Reduction in HIV acquisition risk due to taking ≥4 doses/week of oral TDF/FTC (%)	90–100	MSM trial data
Reduction in HIV acquisition risk due to taking 2–4 doses/week of oral TDF/FTC (%)	56–96	MSM trial data
Reduction in HIV acquisition risk among those using CAB (with CAB adherence observed in HPTN 083; %)	82–96	HPTN 083 trial estimate

Fitting outcomes	Range	Source
Percentage with a PrEP indication 2014		
18- to 24-year-old Black MSM	46–76	Atlanta NHBS
>24-year-old Black MSM	45–61	
18- to 24-year-old White MSM	13–67	
>24-year-old White MSM	47–61	
HIV prevalence 2014 (%)		
18- to 24-year-old Black MSM	19–41	Atlanta NHBS
>24-year-old Black MSM	42–57	
18- to 24-year-old White MSM	1–39	
>24-year-old White MSM	16–34	
Care continuum indicators		
Percentage of HIV-positive MSM diagnosed, 2012	76–83	CDC data for Georgia
Percentage of diagnosed MSM in care, 2014		Georgia DPH data for Georgia
Black	56–66	
White	59–69	
Percentage of diagnosed MSM virally suppressed, 2014		Georgia DPH data for Georgia
Black	38–48	
White	49–59	
Percentage of HIV-negative MSM on PrEP, 2018		Gay pride survey Atlanta
All	16–26	
Black	11–24	
White	18–34	

DPH = Department of Public Health. NHBS = National HIV Behavioral Surveillance.

Race- and age-specific PrEP acceptance (when offered PrEP) and PrEP dropout were based on US PrEP Demo project^7^ data from MSM in San Francisco, Miami, and Washington DC (Table 1). In the absence of data on real-world CAB use, PrEP acceptance was assumed to be the same for both TDF/FTC and CAB. PrEP dropout was also assumed to be the same for both TDF/FTC and CAB based upon similar retention and product discontinuation for the TDF/FTC and CAB arms of the HPTN 083 trial, and in the absence of real-world CAB use data. TDF/FTC efficacy was based on previously published analysis of iPrEx MSM trial data^5^ (Table 1). Race-/age-specific TDF/FTC adherence (% taking ≥4 doses/week) was based on US PrEP Demo project^7^ data (Table 1), and partial adherence among other PrEP users (% taking 2–4 doses/week) on HPTN 083 trial data.^9^ CAB effectiveness (efficacy × adherence) of 91% (82–96%) was calculated by combining the effectiveness results from the HPTN 083 trial (CAB vs. TDF/FTC)^9^ with a meta-regression estimate of the effectiveness of TDF/FTC vs. hypothetical placebo using TDF/FTC adherence data from HPTN 083^18^ (see Supplementary Material for details).

The model was calibrated to the total population size, age- and race-distribution, and PrEP-indication proportion of the MSM population in Atlanta (from National HIV Behavioural Surveillance (NHBS)^19^ data for Atlanta MSM 2004–2014), HIV prevalence (NHBS 2011, 2014) among MSM in Atlanta, race-specific engagement with HIV care and viral suppression among MSM in Georgia (Georgia Department of Public Health (DPH) data 2011–2014) and overall engagement and viral suppression among MSM in Georgia (Georgia DPH 2011–2018), to overall levels of awareness of HIV-positive status (Georgia state 2012), and overall and age-/race-specific PrEP usage (proportion of uninfected MSM reporting using PrEP; Atlanta NHBS 2014, 2017,^2^ other surveys 2013–18^20^, ^21^, ^22^) among MSM in Atlanta (latest values in Table 1, all values in Table S2).

PrEP indication was based on 2017 US Public Health Service recommendations for MSM,^17^ approximated using data available in NHBS surveys as reporting: 1) ≥2 male partners AND either condomless anal sex or a bacterial STD in the past 12 months, or 2) a main HIV-infected male partner in the past 12 months.^23^

Model outputs were also validated against race-specific levels of engagement with HIV care and viral suppression among all people living with HIV in Georgia 2016–2018 and race-specific NHBS HIV prevalence from 2017.

### Model calibration

The model was calibrated simultaneously to multiple data sources (listed above) in a Bayesian framework by assigning plausible ranges to model parameters based on available data (exploring wide ranges in the absence of data; Table S1), using Latin Hypercube Sampling to select seven million different combinations of parameters from these ranges, running the model from 1979 to 2021 using each parameter combination in turn, comparing model outputs (demographic, epidemiological, HIV care continuum, PrEP use) to calibration data, and selecting those combinations of parameters (model ‘fits’) for which model outputs fell within all the pre-determined ranges estimated from the data (Table S2).

The model was coded, solved, and calibrated in C++ using CodeBlocks v17.12.

### Analysis

For all scenarios, the model was run from 1979 to 2021 using each model fit in turn. From 2022 to 2031, the scenarios differed as follows:

0. Base case scenario with no PrEP use: All those on TDF/FTC were taken off PrEP, PrEP initiation set to 0, all other parameters were held constant at their 2021 values.

PrEP scenarios:

1. TDF/FTC continuation: All PrEP-related parameters (including TDF/FTC offer, acceptance, adherence, efficacy, and retention) were held constant at their 2021 values.
2. CAB replacement: All MSM on TDF/FTC switched over to CAB (PrEP efficacy and adherence parameters changed to CAB effectiveness parameter), with all other parameters (including PrEP offer, acceptance, and dropout) held constant at their 2021 values.
3. CAB replacement+expansion (10, 20% increased usage): All MSM on TDF/FTC switched over to CAB (PrEP efficacy and adherence parameters changed to CAB effectiveness parameter), additional uninfected PrEP-indicated MSM started CAB to increase the number of PrEP users by 10% or 20%, the PrEP offer rate was increased to maintain the 10% or 20% higher number of PrEP users (vs. oral PrEP users in 2025), all other parameters (including PrEP dropout) held constant at 2021 values. 10% and 20% increased usage were chosen as plausible increases, given the proportion indicated for PrEP (∼50%).

In all these scenarios, only PrEP-indicated MSM used PrEP.

### PrEP adherence assumptions sensitivity analysis

We tested the sensitivity of our results to our assumption that TDF/FTC adherence was similar to that for MSM in the PrEP Demo project, rerunning our model scenarios assuming that TDF/FTC adherence was similar to that observed among US MSM in the HPTN 083 trial (on average four percentage points higher among Black MSM but 13 percentage points lower among White MSM and two percentage points lower overall than PrEP Demo project levels; Table 1).^24^

### Contribution of PrEP to reaching the EHE goals

We estimated progress towards the EHE goals (75% reduction in new HIV infections in 2025 and 90% reduction in new infections in 2030, compared with 2017) for this population for each scenario, using TDF/FTC adherence levels from the PrEP Demo project (as in the main analysis). We also estimated the level of TDF/FTC or CAB usage required to meet these goals, increasing PrEP initiation and retention and expanding PrEP initiation to those without a PrEP indication and those never testing for HIV (after increasing usage among those with a PrEP indication to 90%).

### Outcome measures

The impact of TDF/FTC and CAB were measured as the % of cumulative infections averted over 5 and 10 years from the start of 2022, in comparison with the base case (no PrEP) scenario (scenario 0), or (for CAB) in comparison with the scenario with TDF/FTC continuation (scenario 1). This latter comparison we term incremental impact; it estimates the % of infections that would occur with continued TDF/FTC use which are averted by CAB. Progress toward EHE goals was measured by comparing annual new infections during 2025 and 2030 with the number during 2017.

### Data statement

All of the data used for model parameterisation and calibration can be found in Tables S1 and S2 in the Supplement. The model code is available from the authors on request.

### Role of the funding source

The funders had no role in study design, collection, analysis, or interpretation of data, writing the report, or the decision to submit the paper for publication. Authors were not precluded from accessing the underlying data in the study and all authors had final responsibility for the decision to submit for publication.

## Results

### Model fits

**Fig. 1:**
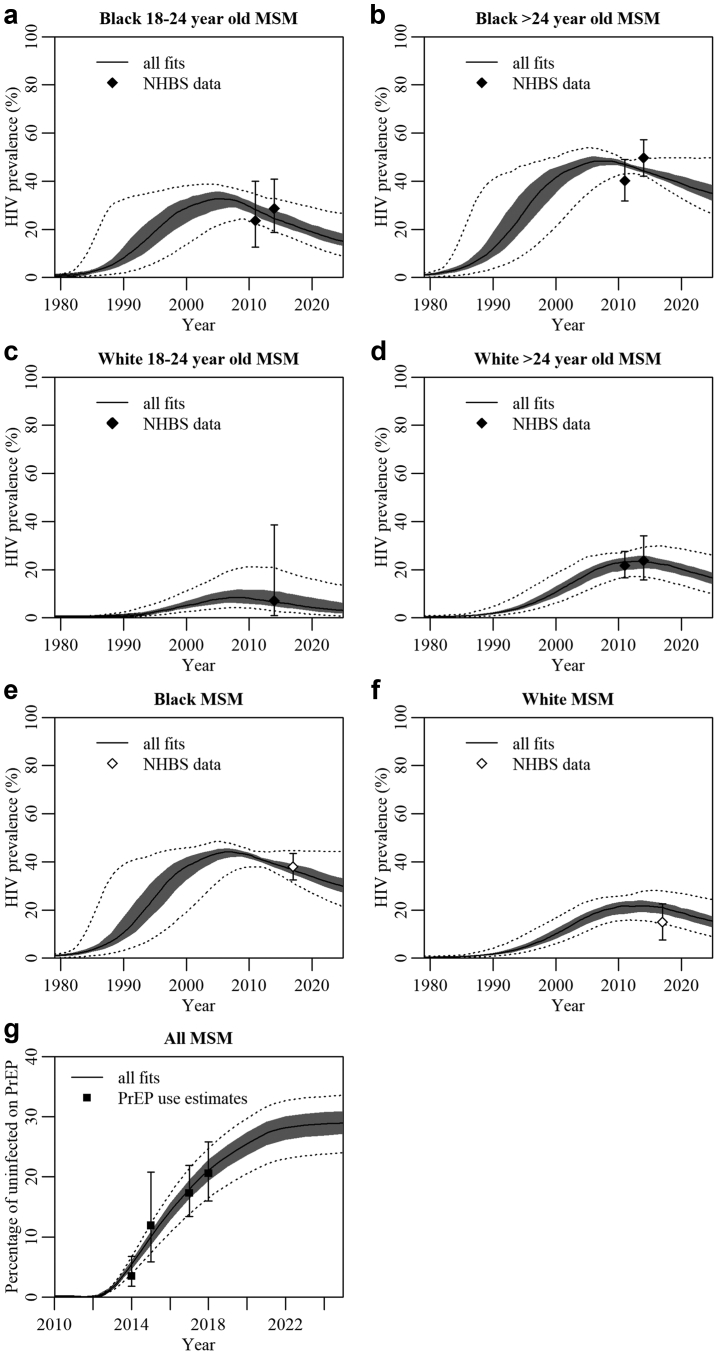
**Model fits to available data for men who have sex with men (MSM) in Atlanta.** (a–d) HIV prevalence among Black young (18- to 24-year-old), Black older (>24-year-old), White young, White older MSM, (e and f) HIV prevalence among Black, White MSM, (g) percentage of all uninfected MSM on PrEP. Results are for all 114 fitting parameter combinations. Results show median (thick lines), 25th to 75th percentile (dark shaded area), and 2.5th and 97.5th percentiles (dotted lines) across model fits. Points and error bars show the mean and 95% CI for data. Black filled points used for model fitting, white points for model validation. Note the different axis scale used in (g).

We found 114 unique parameter combinations which fitted the data (Fig. 1, Fig. S4). The model fitted age- and race-specific HIV prevalence fairly well (Fig. 1a–d) and predicted HIV prevalence among Black MSM in 2017 very well (Fig. 1e), but slightly overestimated HIV prevalence among White MSM in 2017 (Fig. 1f). The model captured the age distribution of MSM surveyed in the NHBS well (Fig. S4a) and captured the racial composition of the NHBS survey population up to 2011, although the proportion of Black participants was smaller than in the NHBS population in subsequent years (Fig. S4b), in line with smaller changes in the race composition of the wider Atlanta population in census data. The model fitted the proportion of older MSM with a PrEP indication very well and was consistent with the most recent PrEP indication estimates for younger MSM (Fig. S4c–f). The model fitted data on HIV-positive status awareness, engagement in care, and viral suppression well (Fig. S4g–n). The model fitted estimates of overall PrEP usage very well (Fig. 1g) and was able to predict recent PrEP use among White MSM (Fig. S4s) while capturing uncertainty around PrEP use among Black MSM due to differences between data sources (Fig. S4o, p and r).

### Projected PrEP usage and HIV incidence trends

**Fig. 2:**
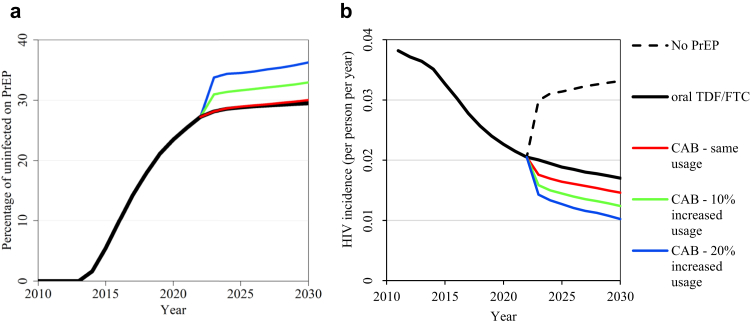
**Modelled trends in(a)PrEP usage and(b)HIV incidence over time under different PrEP scenarios.** Values shown are median values across 114 fits. In panel (a), note that the percentage on PrEP is very similar for the ‘oral TDF/FTC’ and ‘CAB – same usage’ scenarios. Note that PrEP initiation rates are assumed to increase until the start of 2022, and then to remain constant subsequently. Only MSM with a PrEP indication use PrEP, but usage is shown out of all uninfected MSM.

Across the model fits, a median 46% (range 38–53%) of uninfected MSM had a PrEP indication in 2021. With PrEP initiation rates kept constant from 2022 onwards, and PrEP only used by PrEP-indicated MSM, overall TDF/FTC PrEP usage (percentage of all uninfected MSM on PrEP) increased slightly from 28.2% (95% credible interval (CrI) 23.0–32.7%) at the start of 2022 to 29.6% (24.6–34.4%) at the start of 2030 (Fig. 2a). For CAB replacement, usage was very similar, increasing from 28.2% (23.0–32.7) to 30.1% (24.8–34.8%) over the same period (the very slightly increased usage is due to reduced incidence on CAB vs. TDF/FTC). Scenarios assuming 10% and 20% CAB expansion (with only PrEP-indicated MSM using PrEP) over the next 10 years resulted in 33.2% and 36.6% overall CAB usage at the start of 2030 (Fig. 2a).

Modelled HIV incidence among the MSM population in Atlanta declined from a median 3.8% in 2011 (consistent with 3.8% incidence measured in the InvolveMENt cohort, 2010–2014^25^) to 2.1% in 2021 (Fig. 2b). If PrEP use ceases from 2022 onwards, incidence is projected to increase and flatten out around 3.4%. If current levels of PrEP initiation are maintained (using TDF/FTC or CAB) incidence continues to decline, with incremental benefits from replacing TDF/FTC with CAB and expanding CAB usage by 10% and 20%, resulting in 14%, 27%, and 40% reduced HIV incidence in 2030, respectively, vs. TDF/FTC continuation (Fig. 2b).

### PrEP impact

**Fig. 3:**
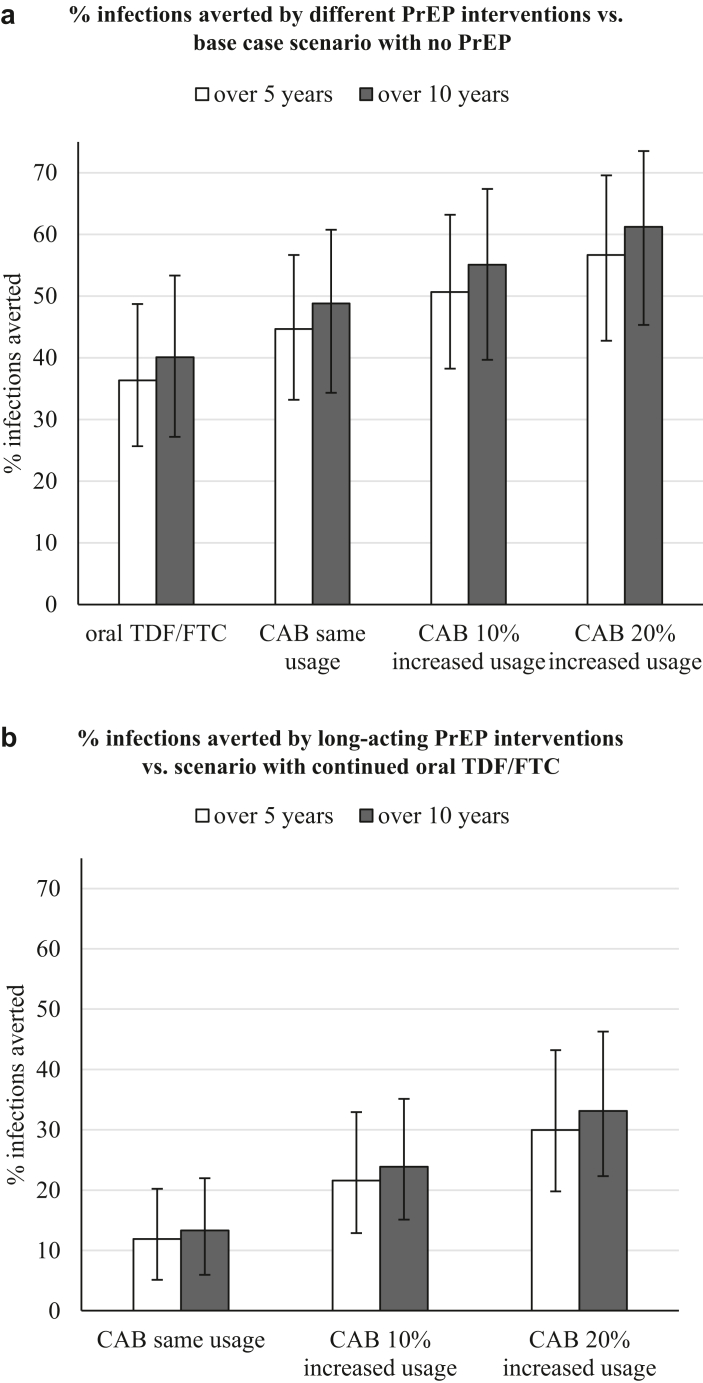
**PrEP impact on new HIV infections.** % of infections averted over 5 years (2022–2026) and 10 years (2022–2031), for different PrEP interventions shown. (a) Impact calculated in comparison with no PrEP base case scenario, (b) impact of CAB calculated in comparison with continued TDF/FTC use scenario (“incremental impact”). Only MSM with a PrEP indication use PrEP. Bars and error bars show median and 95% credible interval across 114 fits.

Our modelling analysis predicted that TDF/FTC continuation, with PrEP use restricted to PrEP-indicated MSM, would avert 36.3% of expected cumulative incident HIV infections (95% CrI 25.6–48.7%) among Atlanta MSM over 5 years (2022–2026), compared with the no-PrEP base-case scenario. Switching all MSM from TDF/FTC to CAB could prevent more infections on average than TDF/FTC: 44.6% (33.2–56.6%) over 2022–2026 compared with no PrEP (Fig. 3a). Increasing levels of CAB usage by 10 or 20% (with PrEP use only among those PrEP-indicated) resulted in 50.6% and 56.7% of infections averted over 5 years compared with no PrEP, respectively. Predicted 10-year proportional impacts (2022–2031) vs. no PrEP were 8–10% greater than 5-year impacts, respectively, across all the different PrEP scenarios (Fig. 3a).

Replacing daily TDF/FTC with CAB always (in all 114 fits) had a positive incremental impact, with CAB preventing 11.9% (5.2–20.2%) of the HIV infections which would otherwise occur with TDF/FTC continuation, over 5 years (2022–2026; Fig. 3b). With 10% and 20% CAB expansion, the projected 5-year incremental impact over the TDF/FTC continuation scenario rose to 21.6% (12.9–32.9%) and 30.0% (19.8–43.2%), respectively. These proportional differences are projected to grow slightly over time to 23.9% and 33.1% over 10 years, respectively (Fig. 3b).

### PrEP adherence assumptions sensitivity analysis

If adherence to TDF/FTC was like that estimated in HPTN 083, we projected a similar median impact (0.5 percentage points smaller) over 5 years of TDF/FTC vs. no PrEP to the main analysis (with PrEP Demo project adherence; Fig. S5a). This resulted in a similar incremental impact of CAB over continued TDF/FTC use, averting 12.1% (6.3–20.4%) of infections that would have otherwise occurred over 5 years with continued TDF/FTC usage at the same level (Fig. S5b).

### Contribution of PrEP to reaching the EHE goals

**Fig. 4:**
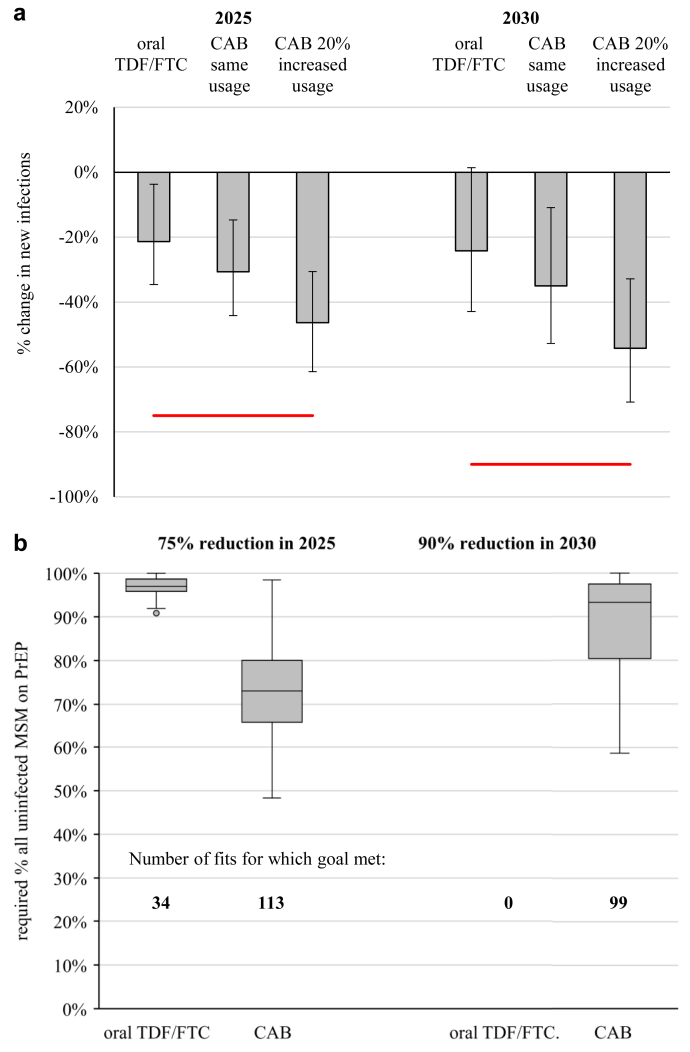
**Contribution of PrEP to reaching EHE goals.** (a) Percentage change in number of new infections occurring in 2025 and 2030 compared with 2017 for different PrEP interventions shown. (b) Percentage of all uninfected MSM who must be using PrEP by 2025 to achieve a 75% reduction in new HIV infections in 2025 (vs. 2017) and a 90% reduction in 2030, when PrEP users use daily oral TDF/FTC or long-acting injectable CAB. In (a), bars and error bars show median and 95% credible interval across 114 fits. Horizontal red lines show the EHE goals of a 75% reduction in 2025 and a 90% reduction in 2030. In (b) central line shows the median, box the interquartile range (25th–75th percentiles), whiskers extend up to 1.5× the inter-quartile range, and dots show outliers. Numbers on plot are the number of fits (out of 114) for which it was possible to reach the goal. In (a), only MSM with a PrEP indication use PrEP. In (b), both MSM with and without a PrEP indication may use PrEP.

With continuing TDF/FTC use assuming 2017 US PrEP indication guidelines, PrEP Demo project TDF/FTC adherence, and no HIV care cascade improvements, we predict a median 22% reduction in numbers of new infections in 2025 vs. 2017, and 24% in 2030 (Fig. 4a). For CAB with the same usage, we see a 31% reduction in new infections in 2025, rising to 36% in 2030 (vs. 2017). With 20% increased CAB usage, we predict a 47% reduction in 2025, and 54% in 2030 (Fig. 4a). These all fall short of the EHE goals of a 75% reduction in 2025, and 90% in 2030.

Expanding daily oral TDF/FTC PrEP use among those with and without a PrEP indication, the 2025 EHE goal (75% reduction in new HIV infections vs. 2017) could only sometimes be met (for 34/114 fits) if no improvements were made to ART use and adherence, with TDF/FTC used by a median 97% (95% CrI 92–100%) of all uninfected MSM by 2025 to reach the 2025 goal (Fig. 4b). The 2030 90% reduction goal could not be met expanding TDF/FTC alone, even with 100% TDF/FTC usage. With CAB, the 2025 and 2030 goals could be met if 73% (53–96%) and 93% (68–100%) of all uninfected MSM (including ≥90% of PrEP-indicated MSM) used CAB by 2025 (Fig. 4b).

## Discussion

Our modelling analysis suggests that continuation of current daily oral TDF/FTC PrEP use among Atlanta MSM would avert around 36% of new HIV infections that would otherwise occur over 2022–2026 without any PrEP use, while switching all these TDF/FTC users to regimens containing long-acting injectable cabotegravir would prevent an average 45% of new infections compared with no PrEP use, and around 12% of the remaining HIV infections that would otherwise occur with continued TDF/FTC use, if adherence to CAB were like HPTN 083. Increasing overall PrEP usage as well as switching TDF/FTC users to CAB would bring additional substantial prevention benefits, with a switch to CAB accompanied by a 20% increase in PrEP use increasing the incremental 5-year impact of CAB over TDF/FTC more than two-fold, to 30% of infections averted. Without further improvements to the treatment cascade in the MSM population, these changes in PrEP use on their own would not be sufficient to meet the EHE goals. Nevertheless, they could make a substantial contribution, with a switch to CAB and a 20% increase in PrEP usage predicted to achieve over half the EHE goal reduction, alongside other existing measures. To achieve the EHE goal of a 90% reduction in new HIV infections by 2030, we estimate 93% of all uninfected MSM would need to use CAB, which is unrealistic. This highlights that other measures, such as improvements in diagnosis and viral suppression, will also likely be needed to meet the EHE goals.

We estimated similar incremental impact (of 12%) of CAB compared with TDF/FTC continuation when we assumed adherence to TDF/FTC like that observed among US MSM in the HPTN 083 trial rather than for participants in the US PrEP Demo project. Although the two studies estimated different race-specific levels of TDF/FTC adherence, they resulted in similar overall TDF/FTC adherence in our modelled population (69% taking ≥4 doses/week using US PrEP Demo project data, 67% using HPTN 083 data). PrEP adherence levels from four cities in the 2017 NHBS^26^ are similar to those in the PrEP Demo project^7^ (58% vs. 57% among Black MSM, 87% vs. 91% among White MSM), adding confidence that our TDF/FTC PrEP adherence assumptions are realistic.

Our findings assume that adherence to CAB will be similar to that seen in the HPTN 083 trial, but real-world adherence could be different. Data on adherence to CAB injection schedules is being collected in the open-label extension to HPTN 083.

In agreement with our findings, a previous modelling study by Jenness et al. found that major expansion of oral TDF/FTC would not be sufficient to meet the EHE goals among MSM in Atlanta.^27^ Their modelling suggested that the 2025 EHE goal (75% reduction in new HIV infections) could be met for this population with ambitious ten-fold improvements in HIV testing rates and HIV care retention, with oral PrEP initiation linked to testing (increasing as HIV testing rates increase). This same intervention was predicted to subsequently lead to a 90% reduction in new HIV infections in 2032 (2 years after the 2030 target date).^27^ It would be interesting to assess the conditions under which the addition of CAB alongside improvements to HIV testing and care retention could help reach the 2030 EHE target in this population.

Two modelling studies have evaluated the likely impact of CAB regimens among MSM, both for Atlanta.^14^^,^^15^ Both these previous studies estimated a similar incremental impact of CAB vs. TDF/FTC continuation to us (e.g., 13% infections averted by CAB vs. TDF/FTC over 10 years^14^ vs. 13% in our main analysis). Marshall et al. estimated that CAB use by 35% of uninfected MSM would prevent 44% of new infections over 10 years, compared with no PrEP use,^14^ slightly lower than our estimate of 49% over 10 years with lower (30%) CAB usage, likely because their model did not prioritise PrEP by indication or risk.

Our results confirmed the conclusion from previous modelling studies that the largest incidence reduction could be achieved if CAB availability led to increased overall PrEP usage.^14^^,^^15^ There are no ‘real-world’ data available yet on whether offering CAB will increase overall PrEP user numbers, but preference studies suggest that it could, with 25–47% of (mostly PrEP naïve) US MSM reporting they would prefer injectable PrEP.^12^^,^^13^ However, widespread use of CAB could be restricted by PrEP stigma, insurance barriers, staffing and workflow constraints, or its cost in comparison with generic TDF/FTC.^28^^,^^29^ The legal requirement that PrEP services be provided without cost to US consumers^30^ may increase PrEP initiation, but this remains to be quantified. Levels of PrEP usage are affected by retention as well as initiation. In agreement with data from the HPTN 083 trial, and in the absence of real-world data, we assumed in this analysis that dropout from TDF/FTC and CAB were the same. If dropout from CAB regimens turns out to be lower than dropout from TDF/FTC, then switching MSM to CAB regimens could lead to increased PrEP coverage and impact. Conversely, higher dropout from CAB vs. TDF/FTC regimens could lead to lower PrEP coverage and impact if PrEP initiations were to stay constant.

An advantage of our study is that we were able to use initial estimates of overall effectiveness (efficacy × adherence) of CAB from a human trial, HPTN 083, while previous studies relied on animal model estimates. A strength of our model is that it was parametrised and fitted to local data on HIV risk behaviour, demography, HIV prevalence, viral suppression, and PrEP use among MSM in Atlanta, stratified by age and race wherever possible.

However, there are limitations to our analysis, including the following. We only evaluated situations where all MSM used TDF/FTC, or all used CAB, and did not look at more realistic scenarios where both modes are used by different people. However, our projections with all TDF/FTC and all CAB use represent lower and upper bounds on the plausible impact of programmes using both modes of PrEP. We assume that only MSM with PrEP indications use PrEP, whereas in reality not all those taking PrEP have an indication, meaning we may overestimate PrEP impact for a given usage level. We used trial data to inform CAB effectiveness, which may exceed levels seen under ‘real-life’ conditions, meaning we may have overestimated CAB impact. Our modelling does not account for heterogeneity in CAB efficacy. Subgroup analyses for HPTN 083 did not detect substantial heterogeneity in CAB effect between groups,^9^ but future modelling could explore the potential implications of heterogeneities in efficacy if evidence of this does emerge. In our main analysis, we used TDF/FTC adherence data from PrEP Demo project participants, which may not reflect TDF/FTC adherence among current users in Atlanta. However, our sensitivity analysis using TDF/FTC adherence based on HPTN 083 participants led to similar estimates of impact. Much of the behavioural and HIV prevalence data used in our model came from NHBS surveys, which used time-location sampling and may not be fully representative of the wider MSM population in Atlanta. Additionally, our model did not take into account sexual positioning, sero-sorting, or sero-positioning, which may impact how HIV spreads through this population.

Our impact estimates were sensitive to CAB usage levels. Data on real-world CAB acceptance, adherence, and retention will be crucial to refine our estimates; planned open-label extensions and demonstration projects will be valuable in informing future modelling work.

In conclusion, our findings suggest that switching current daily oral TDF/FTC users to CAB may prevent 12% of HIV infections that would occur over 5 years with TDF/FTC continuation, if CAB adherence is similar to that in HPTN 083. CAB could have a greater impact if MSM start CAB who would not have used PrEP otherwise; if 37% of uninfected MSM used CAB (giving CAB only to those with a PrEP indication), this could get around 60% of the way to reaching the EHE goals. However, it is unlikely the EHE goals can be met through expansion of CAB alone, even if CAB is also offered to those without a PrEP indication. Future research into real-life CAB acceptance, adherence, and retention by groups with higher exposure to HIV, including young or Black MSM in the South, will be valuable to assess the potential additional benefits of CAB for HIV prevention in the US.

## Contributors

K.M.M., M.-C.B., M.M., and D.T.D. contributed to the conception and design of the modelling analysis. J.T., G.P.-B., C.W., A.L., D.J.D., B.G., and R.J.L. contributed data. K.M.M., B.H., C.W., D.J.D., and A.L. verified the underlying data. B.H. conducted the CAB effectiveness analysis. K.M.M. analysed other data to inform the model. K.M.M. did the modelling analysis. M.M. and D.T.D. provided advice on technical aspects of the modelling analysis. K.M.M., M.-C.B., B.H., M.M., G.P.-B., A.L., R.J.L., and D.T.D. contributed to interpreting the modelling results. K.M.M. drew the figures and wrote the first draft of the manuscript. All authors contributed to manuscript revision and critical review.

## Declaration of interests

Prof Boily, and Dr Mitchell, Prof Donnell and Dr Dimitrov report grants from the National Institutes of Health, during the conduct of the study. Dr Mitchell reports a fellowship from the Wellcome Trust and an honorarium for speaking from Gilead Sciences and has received payment from Pfizer for teaching of the mathematical modelling of infectious disease transmission, outside the submitted work. Dr Liu reports funding for investigator sponsored research from Gilead Sciences and ViiV Healthcare and has led studies in which Gilead has donated study drug, and payment from IAS-USA for manuscript preparation, outside the submitted work. Prof Donnell reports funding from USAID and the Bill and Melinda Gates Foundation, outside the submitted work. Prof Landovitz reports funding from the National Institutes of Health and ViiV Healthcare, scientific advisory board payments from Gilead Sciences and Merck Inc, and consultancy payments from Cepheid and Janssen. All other authors declare no competing interests.

## Appendix A Supplementary data

Supplementary Material

Caption for Supplementary Material
