# Supplementary material for: Estimating the impact of HIV PrEP regimens containing long-acting injectable cabotegravir or daily oral tenofovir disoproxil fumarate/emtricitabine among men who have sex with men in the United States: a mathematical modelling study for HPTN 083
Source: Lancet Reg Health Am. 2023 Jan 17;18:100416. doi: 10.1016/j.lana.2022.100416 (PMC9950652; doi:10.1016/j.lana.2022.100416)
Supplement: Supplementary Material [file mmc1.docx]

**Supplementary material**

**Estimating the impact of HIV PrEP regimens containing long-acting injectable cabotegravir or daily oral tenofovir disoproxil fumarate/emtricitabine among men who have sex with men in the United States: a mathematical modelling study for HPTN 083**

Kate M Mitchell, Marie-Claude Boily, Brett Hanscom, Mia Moore, Jeffery Todd, Gabriela Paz-Bailey, Cyprian Wejnert, Albert Liu, Deborah Donnell, Beatriz Grinsztejn, Raphael J Landovitz, Dobromir T Dimitrov

**Contents**

[SUPPLEMENTAL METHODS 2](#_Toc104218846)

[Model structure 2](#_Toc104218847)

[Model equations 7](#_Toc104218848)

[Model parameters 14](#_Toc104218882)

[Parameter table 17](#_Toc104218883)

[Table of fitting data 27](#_Toc104218884)

[SUPPLEMENTAL RESULTS 30](#_Toc104218885)

[References 35](#_Toc104218886)

# SUPPLEMENTAL METHODS

## Model structure

In the model equations, uninfected MSM are denoted by $X_{v,w,u}^{z}$, those with acute HIV infection by $A_{v,w,u}^{z}$ and MSM with chronic HIV infection by $Y_{v,w,u}^{x,y,z}$. The subscripts represent the following: $v$ is age (0 = 18-24 years old, 1 = >24 years old), $w$ is race (0 = Black, 1 = White), $u$ is PrEP indication (0 = not indicated for PrEP (lower risk), 1 = indicated for PrEP (higher risk)). The superscripts represent: $x$ is CD4 count (1 = CD4>500, 2 = CD4 350-500, 3 = CD4 200-350, 4 = CD4 <200 cells per µL), y is set-point viral load (SPVL; 1 = Log_10_ SPVL <4.0, 2 = Log_10_ SPVL 4.0-4.5, 3 = Log_10_ SPVL 4.5-5.0, 4 = Log_10_ SPVL >5.0), $z$ is care state (0 = never testing, 1 = testing but undiagnosed, 2 = on PrEP, 3 = diagnosed not linked to care, 4 = linked into HIV care, 5 = on ART, adherent and partially suppressed, 6 = in first year on ART, adherent and fully suppressed, 7 = 2^nd^ year on ART adherent and fully suppressed, 8 = 3^rd^ and subsequent years on ART adherent and fully suppressed, 9 = on ART but non-adherent and not suppressed, 10 = stopped taking ART (due to failure or dropout)). The only possible care states for uninfected MSM are $z$=0, 1 or 2. MSM with acute HIV infection may be any of care states $z$=0-5; after achieving full viral suppression on ART they are assumed to no longer be in the acute stage.

Figure S1 shows the different modelled age, race and PrEP indication groups. A proportion $m_{v,w,u}$ of MSM entering the modelled sexually active MSM population enter each combination of age, race and PrEP-indication group. The proportion is calculated from $m_{black}$, the time-varying proportion of incoming MSM who are Black, $m_{young,w}$, the constant proportion of incoming MSM of each race who are aged 18-24 years old, and $m_{v,w,high}$, the constant proportion of incoming MSM in each race and age group meeting the criteria for a PrEP indication. MSM move from the younger (18-24 year old) to the older (>24 year old) age groups at a race-specific annual rate $\pi_{w}$, reflecting the average number of years (${1/\pi}_{w}$) that MSM of race $w$ are sexually active in Baltimore between the ages of 18 and 24 years old.

Figure S2 shows the transitions between different HIV disease states in the model for those not virally suppressed (i.e. for everyone apart from those on ART and adherent, who are either partially or fully virally suppressed), by CD4 count and SPVL. These transitions are the same for all age, race and PrEP indication groups, and for all care groups apart from those virally suppressed, with the exception of $\mu$, the background mortality rate, which varies by age and race, and $\lambda$, the rate of HIV infection, which varies by age, race, PrEP indication and PrEP use.

All MSM entering the modelled population are assumed to be uninfected with HIV (susceptible;$X^{z}$). MSM acquire HIV infection at a rate $\lambda_{v,w,u,z}$, which varies by age, race, PrEP indication (risk) and PrEP use, moving initially into the short acute infection stage, $A^{z}$ (of average duration $1/{\gamma_{a}}$). At the end of the acute infection stage, infected MSM in the model move into one of 16 chronic infection states ($Y^{x,y,z}$), distinguished by CD4 count and SPVL, with a proportion $\theta_{y}$ entering each SPVL strata, and for each SPVL strata, $y$, a proportion $f_{x,y}$ starting in each CD4 category, $x$. Chronically infected individuals are assumed in the model to retain the same SPVL over time, and in the absence of treatment, to move sequentially through progressively lower CD4 states, at a rate $\gamma_{x,y}$, with faster progression for those with higher SPVL.

Modelled MSM with HIV experience HIV-related mortality and leave the model at a rate, $\alpha_{x,y,z}$, related to their CD4 count, SPVL and care status. Lower CD4 counts and higher SPVL are associated with higher rates of HIV-related mortality, viral suppression with lower HIV-related mortality. All MSM in the model experience non-HIV-related (background) mortality and leave the model at a rate $\mu_{v,w}$, which varies by age and race.

Figure S3 shows the transitions between different care states in the model. Of the MSM entering the model ($\Gamma$ per year), a proportion, $p_{w}$, which varies by race, are assumed to never undergo routine HIV testing, and enter the never testing ($X^{0}$) compartment. The remainder ($1-p_{w}$) will undergo routine HIV testing and enter the testing (undiagnosed) compartment ($X^{1}$). Uninfected MSM in either the never testing or testing compartment can acquire HIV at a rate $\lambda_{1}$. Uninfected MSM who undergo routine testing (at a rate $\tau_{1,v,w}$, which varies with age and race), may be offered PrEP (at a relative rate $\delta_{u}$ (proportional to their testing rate) which depends upon their PrEP indication) with a proportion ($o_{v,w}$, which varies with age and race) accepting PrEP and moving into the PrEP compartment ($X_{2}$). Those on PrEP may also acquire HIV, at a rate $\lambda_{2}$. Those on PrEP may drop out, at a rate $\iota_{v,w}$, which varies with age and race. Those on PrEP are assumed to test regularly for HIV, at a rate $\tau_{2}$.

When undiagnosed infected MSM (including those on PrEP) test for HIV, they are assumed to leave the undiagnosed/PrEP compartments, and a proportion $q_{z,w}$, which varies by race, and whether or not they are on PrEP, are assumed to link rapidly into care and move directly into the ‘linked into care’ compartment ($A^{4}$ for those with acute infection, $Y^{x,y,4}$ for those with chronic infection), with the remainder ($1-q_{z,w}$) initially entering the ‘diagnosed (not linked into care)’ compartment ($A^{3}$ for those with acute infection, $Y^{x,y,3}$ for those with chronic infection). Those in the diagnosed but not linked compartment may be subsequently linked into care at a race-specific rate $\epsilon_{w}$ (entering $A^{4}$ or $Y^{x,y,4}$), and those linked into care may drop out of care at a rate $\omega_{w}\phi_{5,1}$ (where $\phi_{5,1}$ is the rate of dropout from ART among White MSM, and $\omega_{w}$ is the race-specific relative rate of dropout from HIV care vs. dropout from ART), moving into the ‘diagnosed (not linked into care)’ compartment ($A^{3}$ or $Y^{x,y,3}$).

Those linked into care initiate ART at a rate $\xi_{x,w}$, which varies by race, with ART initiation only occurring at certain CD4 counts between 1996-2012, in line with CDC recommendations, and no restriction on ART initiation by CD4 count from 2012 onwards. A race-varying proportion $\chi_{w}$ of those starting ART are assumed to eventually achieve viral suppression, and move into the ‘on ART, partially virally suppressed’ compartments ($A^{5}$ for those with acute infection, $Y^{x,y,5}$ for those with chronic infection). The remainder of those starting ART ($1-\chi_{w}$) are assumed to never achieve viral suppression due to non-adherence to ART, and they enter the ‘on ART, non-adherent, unsuppressed’ compartment ($Y^{x,y,9}$), with those in the acute stage of infection assumed to progress to chronic infection at this point. Those who are non-adherent and unsuppressed are assumed to have the same HIV-related mortality, disease progression and infectiousness as those not on ART (shown in Figure S2). Infected individuals in any untreated care state, including those never routinely testing for HIV, may test for HIV due to AIDS symptoms (related to CD4 count), at a rate $\psi_{x,z}$, and start ART, of whom a proportion $\chi_{w}$ will eventually achieve viral suppression, moving into the ‘on ART, partially virally suppressed’ compartment ($Y^{x,y,5}$), with the remainder ($1-\chi_{w}$) entering the ‘on ART, non-adherent, unsuppressed’ compartment ($Y^{x,y,9}$). Those in the ‘on ART, partially virally suppressed’ compartment are assumed to have reduced infectiousness, intermediate between the infectiousness levels of those not on ART and those who are fully virally suppressed. They remain in this compartment for a few months until they achieve full viral suppression, which takes less time for those with lower SPVL, moving into the first fully virally suppressed compartment ($Y^{x,y,6}$) at a rate $\sigma_{y}$. Those partially virally suppressed who were still in the acute stage of infection are assumed to be in the chronic stage of infection by the time they achieve full viral suppression, entering compartment $Y^{x,y,6}$ at a rate $\sigma_{0}$. Those achieving full viral suppression spend the remainder of their first year on ART in $Y^{x,y,6}$, then move into the second fully suppressed compartment, $Y^{x,y,7}$, where they stay for one year on average, before moving into the third fully suppressed compartment, $Y^{x,y,8}$. Changes in CD4 count among those who are adherent to ART and achieve viral suppression are not explicitly modelled; instead, their survival is modelled as a function of initial CD4 count. Those who are fully suppressed are assumed to not be infectious to their sexual partners. They have reduced HIV-related mortality, which decreases with increased time on ART.

Those on ART may stop taking ART (due to drop out or ART failure) at a rate $\phi_{z,w}$ (which varies by duration on ART and race), entering the ‘stopped ART’ compartment, $Y^{x,y,10}$. Those who were virally suppressed before stopping ART are assumed to rapidly return to the same CD4 count as they had before starting treatment, entering the same CD4 compartment in the model that they were in before starting ART. Those who have stopped taking ART may re-enter HIV care (entering $Y^{x,y,4}$) at a rate $\zeta$. In the model, these individuals may subsequently re-start ART and achieve viral suppression, at the same rate as those who have never taken ART before.

**Fig S1: Age groups, race groups, PrEP indication (risk) groups, and movement between them in the model** **Fig S2. HIV disease progression, by HIV states and SPVL, for those not on ART, and for those on ART but not adherent.** Superscripts on states and subscripts on HIV-related death rates are x,y,z (x = CD4 category; y = set-point viral load category; z = care state); subscripts for age, race and PrEP indication are omitted for clarity.

**Fig S3: Different stages of HIV care and transitions between them.** Superscripts on states and subscripts on HIV-related death rates are x,y,z (x = CD4 category; y = set-point viral load category; z = care state); subscripts for age, race and PrEP indication are omitted for clarity.

## Model equations

*MSM who never get tested for HIV:*

$$\frac{d}{dt}\left( X_{v,w,u}^{0} \right)=\Gamma p_{w}m_{v,w,u}+{v\pi}_{w}X_{1-v,w,u}^{0}-X_{v,w,u}^{0}\left( \lambda_{v,w,u,1}+\mu_{v,w}+\left( 1-v \right)\pi_{w} \right)$$

$$\frac{d}{dt}\left( A_{v,w,u}^{0} \right)=\lambda_{v,w,u,1}X_{v,w,u}^{0}+{v\pi}_{w}A_{1-v,w,u}^{0}-A_{v,w,u}^{0}\left( \gamma_{a}+\mu_{v,w}+\alpha_{0,0,0}+\left( 1-v \right)\pi_{w}+\psi_{0,0} \right)$$

$$\frac{d}{dt}\left( Y_{v,w,u}^{1,y,0} \right)=\gamma_{a}{\theta_{y}f_{1,y}A}_{v,w,u}^{0}+{v\pi}_{w}Y_{1-v,w,u}^{1,y,0}-Y_{v,w,u}^{1,y,0}\left( \gamma_{1,y}+\mu_{v,w}+\alpha_{1,y,0}+\left( 1-v \right)\pi_{w}+\psi_{1,0} \right)$$

$$\frac{d}{dt}\left( Y_{v,w,u}^{x,y,0} \right)=\gamma_{a}{\theta_{y}f_{x,y}A}_{v,w,u}^{0}+\gamma_{x-1,y}Y_{v,w,u}^{x-1,y,0}+{v\pi}_{w}Y_{1-v,w,u}^{x,y,0}-Y_{v,w,u}^{x,y,0}\left( \gamma_{x,y}+\mu_{v,w}+\alpha_{x,y,0}+\left( 1-v \right)\pi_{w}+\psi_{x,0} \right);x\in\left\{ 2,3 \right\}$$

$$\frac{d}{dt}\left( Y_{v,w,u}^{4,y,0} \right)=\gamma_{a}{\theta_{y}f_{4,y}A}_{v,w,u}^{0}+\gamma_{3,y}Y_{v,w,u}^{3,y,0}+{v\pi}_{w}Y_{1-v,w,u}^{4,y,0}-Y_{v,w}^{4,y,0}\left( \mu_{v,w}+\alpha_{4,y,0}+\left( 1-v \right)\pi_{w}+\psi_{4,0} \right)$$

*MSM who may get tested, not diagnosed:*

$$\frac{d}{dt}\left( X_{v,w,u}^{1} \right)=\Gamma\left( 1-p_{w} \right)m_{v,w,u}+{v\pi}_{w}X_{1-v,w,u}^{1}+\iota_{v,w}X_{v,w,u}^{2}-X_{v,w,u}^{1}\left( \lambda_{v,w,u,1}+\mu_{v,w}+\left( 1-v \right)\pi_{w}+\delta_{u}{o_{v,w}\tau}_{v,w,1} \right)$$

$$\frac{d}{dt}\left( A_{v,w,u}^{1} \right)=\lambda_{v,w,u,1}X_{v,w,u}^{1}+{v\pi}_{w}A_{1-v,w,u}^{1}+\iota_{v,w}A_{v,w,u}^{2}-A_{v,w,u}^{1}\left( \gamma_{a}+\mu_{v,w}+\alpha_{0,0,1}+\left( 1-v \right)\pi_{w}+\psi_{0,1}+\tau_{v,w,1} \right)$$

$$\frac{d}{dt}\left( Y_{v,w,u}^{1,y,1} \right)=\gamma_{a}{\theta_{y}f_{1,y}A}_{v,w,u}^{1}+{v\pi}_{w}Y_{1-v,w,u}^{1,y,1}+\iota_{v,w}Y_{v,w,u}^{1,y,2}-Y_{v,w,u}^{1,y,1}\left( \gamma_{1,y}+\mu_{v,w}+\alpha_{1,y,1}+\left( 1-v \right)\pi_{w}+\psi_{1,1}+\tau_{v,w,1} \right)$$

$$\frac{d}{dt}\left( Y_{v,w,u}^{x,y,1} \right)=\gamma_{a}{\theta_{y}f_{x,y}A}_{v,w,u}^{1}+\gamma_{x-1,y}Y_{v,w,u}^{x-1,y,1}+{v\pi}_{w}Y_{1-v,w,u}^{x,y,1}+\iota_{v,w}Y_{v,w,u}^{x,y,2}-Y_{v,w,u}^{x,y,1}\left( \gamma_{x,y}+\mu_{v,w}+\alpha_{x,y,1}+\left( 1-v \right)\pi_{w}+\psi_{x,1}+\tau_{v,w,1} \right);x\in\left\{ 2,3 \right\}$$

$$\frac{d}{dt}\left( Y_{v,w,u}^{4,y,1} \right)=\gamma_{a}{\theta_{y}f_{4,y}A}_{v,w,u}^{1}+\gamma_{3,y}Y_{v,w,u}^{3,y,1}+{v\pi}_{w}Y_{1-v,w,u}^{4,y,1}+\iota_{v,w}Y_{v,w,u}^{4,y,2}-Y_{v,w,u}^{4,y,1}\left( \mu_{v,w}+\alpha_{4,y,1}+\left( 1-v \right)\pi_{w}+\psi_{4,1}+\tau_{v,w,1} \right)$$

*MSM taking PrEP:*

$$\frac{d}{dt}\left( X_{v,w,u}^{2} \right)={\delta_{u}{o_{v,w}\tau}_{v,w,1}X_{v,w,u}^{1}+v\pi}_{w}X_{1-v,w,u}^{2}-X_{v,w,u}^{2}\left( \lambda_{v,w,u,2}+\mu_{v,w}+\left( 1-v \right)\pi_{w}+\iota_{v,w} \right)$$

$$\frac{d}{dt}\left( A_{v,w,u}^{2} \right)=\lambda_{v,w,u,2}X_{v,w,u}^{2}+{v\pi}_{w}A_{1-v,w,u}^{2}-A_{v,w,u}^{2}\left( \gamma_{a}+\mu_{v,w}+\alpha_{0,0,2}+\left( 1-v \right)\pi_{w}+\psi_{0,2}+\iota_{v,w}+\tau_{v,w,2} \right)$$

$$\frac{d}{dt}\left( Y_{v,w,u}^{1,y,2} \right)=\gamma_{a}{\theta_{y}f_{1,y}A}_{v,w,u}^{2}+{v\pi}_{w}Y_{1-v,w,u}^{1,y,2}-Y_{v,w,u}^{1,y,2}\left( \gamma_{1,y}+\mu_{v,w}+\alpha_{1,y,2}+\left( 1-v \right)\pi_{w}+\psi_{1,2}+\iota_{v,w}+\tau_{v,w,2} \right)$$

$$\frac{d}{dt}\left( Y_{v,w,u}^{x,y,2} \right)=\gamma_{a}{\theta_{y}f_{x,y}A}_{v,w,u}^{2}+\gamma_{x-1,y}Y_{v,w,u}^{x-1,y,2}+{v\pi}_{w}Y_{1-v,w,u}^{x,y,2}-Y_{v,w,u}^{x,y,2}\left( \gamma_{x,y}+\mu_{v,w}+\alpha_{x,y,2}+\left( 1-v \right)\pi_{w}+\psi_{x,2}+\iota_{v,w}+\tau_{v,w,2} \right);x\in\left\{ 2,3 \right\}$$

$$\frac{d}{dt}\left( Y_{v,w,u}^{4,y,2} \right)=\gamma_{a}{\theta_{y}f_{4,y}A}_{v,w,u}^{2}+\gamma_{3,y}Y_{v,w,u}^{3,y,2}+{v\pi}_{w}Y_{1-v,w,u}^{4,y,2}-Y_{v,w,u}^{4,y,2}\left( \mu_{v,w}+\alpha_{4,y,2}+\left( 1-v \right)\pi_{w}+\psi_{4,2}+\iota_{v,w}+\tau_{v,w,2} \right)$$

*MSM diagnosed but not in care:*

$$\frac{d}{dt}\left( A_{v,w,u}^{3} \right)={v\pi}_{w}A_{1-v,w,u}^{3}+\left( 1-q_{1,w} \right)\tau_{v,w,1}A_{v,w,u}^{1}+\left( 1-q_{2,w} \right)\tau_{v,w,2}A_{v,w,u}^{2}+\omega_{w}\phi_{5,1}A_{v,w,u}^{4}-A_{v,w,u}^{3}\left( \gamma_{a}+\mu_{v,w}+\alpha_{0,0,3}+\left( 1-v \right)\pi_{w}+\psi_{0,3}+\epsilon_{w} \right)$$

$$\frac{d}{dt}\left( Y_{v,w,u}^{1,y,3} \right)=\gamma_{a}{\theta_{y}f_{1,y}A}_{v,w,u}^{3}+{v\pi}_{w}Y_{1-v,w,u}^{1,y,3}+\left( 1-q_{1,w} \right)\tau_{v,w,1}Y_{v,w,u}^{1,y,1}+\left( 1-q_{2,w} \right)\tau_{v,w,2}Y_{v,w,u}^{1,y,2}+\omega_{w}\phi_{5,1}Y_{v,w,u}^{1,y,4}{-Y}_{v,w,u}^{1,y,3}\left( \gamma_{1,y}+\mu_{v,w}+\alpha_{1,y,3}+\left( 1-v \right)\pi_{w}+\psi_{1,3}+\epsilon_{w} \right)$$

$$\frac{d}{dt}\left( Y_{v,w,u}^{x,y,3} \right)=\gamma_{a}{\theta_{y}f_{x,y}A}_{v,w,u}^{3}+\gamma_{x-1,y}Y_{v,w}^{x-1,y,3}+{v\pi}_{w}Y_{1-v,w,u}^{x,y,3}+\left( 1-q_{1,w} \right)\tau_{v,w,1}Y_{v,w,u}^{x,y,1}+\left( 1-q_{2,w} \right)\tau_{v,w,2}Y_{v,w,u}^{x,y,2}+\omega_{w}\phi_{5,1}Y_{v,w,u}^{x,y,4}-Y_{v,w,u}^{x,y,3}\left( \gamma_{x,y}+\mu_{v,w}+\alpha_{x,y,3}+\left( 1-v \right)\pi_{w}+\psi_{x,3}+\epsilon_{w} \right);x\in\left\{ 2,3 \right\}$$

$$\frac{d}{dt}\left( Y_{v,w,u}^{4,y,3} \right)=\gamma_{a}{\theta_{y}f_{4,y}A}_{v,w,u}^{3}+\gamma_{3,y}Y_{v,w,u}^{3,y,3}+{v\pi}_{w}Y_{1-v,w,u}^{4,y,3}+\left( 1-q_{1,w} \right)\tau_{v,w,1}Y_{v,w,u}^{4,y,1}+\left( 1-q_{2,w} \right)\tau_{v,w,2}Y_{v,w,u}^{4,y,2}+\omega_{w}\phi_{5,1}Y_{v,w,u}^{4,y,4}-Y_{v,w,u}^{4,y,3}\left( \mu_{v,w}+\alpha_{4,y,3}+\left( 1-v \right)\pi_{w}+\psi_{4,3}+\epsilon_{w} \right)$$

*MSM in care:*

$$\frac{d}{dt}\left( A_{v,w,u}^{4} \right)={v\pi}_{w}A_{1-v,w,u}^{4}+q_{1,w}\tau_{v,w,1}A_{v,w,u}^{1}+q_{2,w}\tau_{v,w,2}A_{v,w,u}^{2}+\epsilon_{w}A_{v,w,u}^{3}-A_{v,w,u}^{4}\left( \gamma_{a}+\mu_{v,w}+\alpha_{0,0,4}+\left( 1-v \right)\pi_{w}+\psi_{0,4}+\omega_{w}\phi_{5,1}+\xi_{0,w} \right)$$

$$\frac{d}{dt}\left( Y_{v,w,u}^{1,y,4} \right)=\gamma_{a}{\theta_{y}f_{1,y}A}_{v,w,u}^{4}+{v\pi}_{w}Y_{1-v,w,u}^{1,y,4}+q_{1,w}\tau_{v,w,1}Y_{v,w,u}^{1,y,1}+q_{2,w}\tau_{v,w,2}Y_{v,w,u}^{1,y,2}+\epsilon_{w}Y_{v,w,u}^{1,y,3}+\zeta Y_{v,w,u}^{1,y,10}-Y_{v,w,u}^{1,y,4}\left( \gamma_{1,y}+\mu_{v,w}+\alpha_{1,y,4}+\left( 1-v \right)\pi_{w}+\psi_{1,4}+\omega_{w}\phi_{5,1}+\xi_{1,w} \right)$$

$$\frac{d}{dt}\left( Y_{v,w,u}^{x,y,4} \right)=\gamma_{a}{\theta_{y}f_{x,y}A}_{v,w,u}^{4}+\gamma_{x-1,y}Y_{v,w,u}^{x-1,y,4}+{v\pi}_{w}Y_{1-v,w,u}^{x,y,4}+q_{1,w}\tau_{v,w,1}Y_{v,w,u}^{x,y,1}+q_{2,w}\tau_{v,w,2}Y_{v,w,u}^{x,y,2}+\epsilon_{w}Y_{v,w,u}^{x,y,3}+\zeta Y_{v,w,u}^{x,y,10}-Y_{v,w,u}^{x,y,4}\left( \gamma_{x,y}+\mu_{v,w}+\alpha_{x,y,4}+\left( 1-v \right)\pi_{w}+\psi_{x,4}+\omega_{w}\phi_{5,1}+\xi_{x,w} \right);x\in\left\{ 2,3 \right\}$$

$$\frac{d}{dt}\left( Y_{v,w,u}^{4,y,4} \right)=\gamma_{a}{\theta_{y}f_{4,y}A}_{v,w,u}^{4}+\gamma_{3,y}Y_{v,w,u}^{3,y,4}+{v\pi}_{w}Y_{1-v,w,u}^{4,y,4}+q_{1,w}\tau_{v,w,1}Y_{v,w,u}^{4,y,1}+{q_{2,w}\tau}_{v,w,2}Y_{v,w,u}^{4,y,2}+\epsilon_{w}Y_{v,w,u}^{4,y,3}+\zeta Y_{v,w,u}^{4,y,10}-Y_{v,w,u}^{4,y,4}\left( \mu_{v,w}+\alpha_{4,y,4}+\left( 1-v \right)\pi_{w}+\psi_{4,4}+\omega_{w}\phi_{5,1}+\xi_{4,w} \right)$$

*MSM on ART and adherent:*

$$\frac{d}{dt}\left( A_{v,w,u}^{5} \right)={v\pi}_{w}A_{1-v,w,u}^{5}+{\chi_{w}\xi}_{0,w}A_{v,w,u}^{4}+\sum_{Z=0}^{Z=4} {{\chi_{w}\psi}_{0,Z}A}_{v,w,u}^{Z}-A_{v,w,u}^{5}\left( \gamma_{a}+\mu_{v,w}+\alpha_{0,0,5}+\left( 1-v \right)\pi_{w}+\sigma_{0}+\phi_{5,w} \right)$$

$$\frac{d}{dt}\left( Y_{v,w,u}^{x,y,5} \right)={\gamma_{a}{\theta_{y}f_{x,y}A}_{v,w,u}^{5}+v\pi}_{w}Y_{1-v,w,u}^{x,y,5}+\chi_{w}\xi_{x,w}Y_{v,w,u}^{x,y,4}+\sum_{Z=0}^{Z=4} \chi_{w}{\psi_{x,Z}Y}_{v,w,u}^{x,y,Z}+\chi_{w}\psi_{x,10}Y_{v,w,u}^{x,y,10}-Y_{v,w,u}^{x,y,5}\left( \mu_{v,w}+\alpha_{x,y,5}+\left( 1-v \right)\pi_{w}+\sigma_{y}+\phi_{5,w} \right)$$

$$\frac{d}{dt}\left( Y_{v,w,u}^{x,y,6} \right)={v\pi}_{w}Y_{1-v,w,u}^{x,y,6}+\sigma_{0}\theta_{y}f_{x,y}A_{v,w,u}^{5}{+\sigma_{y}Y}_{v,w,u}^{x,y,5}-Y_{v,w,u}^{x,y,6}\left( \mu_{v,w}+\alpha_{x,y,6}+\left( 1-v \right)\pi_{w}+\eta_{y}+\phi_{6,w} \right)$$

$$\frac{d}{dt}\left( Y_{v,w,u}^{x,y,7} \right)={v\pi}_{w}Y_{1-v,w,u}^{x,y,7}+\eta_{y}Y_{v,w,u}^{x,y,6}-Y_{v,w,u}^{x,y,7}\left( \mu_{v,w}+\alpha_{x,y,7}+\left( 1-v \right)\pi_{w}+1+\phi_{7,w} \right)$$

$$\frac{d}{dt}\left( Y_{v,w,u}^{x,y,8} \right)={v\pi}_{w}Y_{1-v,w,u}^{x,y,8}+Y_{v,w,u}^{x,y,7}-Y_{v,w,u}^{x,y,8}\left( \mu_{v,w}+\alpha_{x,y,8}+\left( 1-v \right)\pi_{w}+\phi_{8,w} \right)$$

*MSM on ART but non-adherent:*

$$\frac{d}{dt}\left( Y_{v,w,u}^{1,y,9} \right)=(1-{\chi_{w})\xi}_{0,w}\theta_{y}f_{1,y}A_{v,w,u}^{4}+\left( 1-\chi_{w} \right)\xi_{1,w}Y_{v,w,u}^{1,y,4}+\sum_{Z=0}^{Z=4} {{\left( 1-\chi_{w} \right)\psi}_{0,Z}\theta_{y}f_{1,y}A}_{v,w,u}^{Z}+\sum_{Z=0}^{Z=4} \left( 1-\chi_{w} \right){\psi_{1,Z}Y}_{v,w,u}^{1,y,Z}+(1-\chi_{w})\psi_{x,10}Y_{v,w,u}^{1,y,10}+{v\pi}_{w}Y_{1-v,w,u}^{1,y,9}-Y_{v,w,u}^{1,y,9}\left( \gamma_{1,y}+\mu_{v,w}+\alpha_{1,y,9}+\left( 1-v \right)\pi_{w}+\phi_{9,w} \right)$$

$$\frac{d}{dt}\left( Y_{v,w,u}^{x,y,9} \right)={(1-{\chi_{w})\xi}_{0,w}\theta_{y}f_{x,y}A_{v,w,u}^{4}+\left( 1-\chi_{w} \right)\xi_{1,w}Y_{v,w,u}^{x,y,4}+\sum_{Z=0}^{Z=4} {{\left( 1-\chi_{w} \right)\psi}_{0,Z}\theta_{y}f_{x,y}A}_{v,w,u}^{Z}+\sum_{Z=0}^{Z=4} \left( 1-\chi_{w} \right){\psi_{x,Z}Y}_{v,w,u}^{x,y,Z}+(1-\chi_{w})\psi_{x,10}Y_{v,w,u}^{x,y,10}+v\pi}_{w}Y_{1-v,w,u}^{x,y,9}+\gamma_{x-1,y}Y_{v,w,u}^{x-1,y,9}-Y_{v,w,u}^{x,y,9}\left( \gamma_{x,y}+\mu_{v,w}+\alpha_{x,y,9}+\left( 1-v \right)\pi_{w}+\phi_{9,w} \right);x\in\left\{ 2,3 \right\}$$

$$\frac{d}{dt}\left( Y_{v,w,u}^{4,y,9} \right)=(1-{\chi_{w})\xi}_{0,w}\theta_{y}f_{4,y}A_{v,w,u}^{4}+\left( 1-\chi_{w} \right)\xi_{1,w}Y_{v,w,u}^{4,y,4}+\sum_{Z=0}^{Z=4} {{\left( 1-\chi_{w} \right)\psi}_{4,Z}\theta_{y}f_{4,y}A}_{v,w,u}^{Z}+\sum_{Z=0}^{Z=4} \left( 1-\chi_{w} \right){\psi_{x,Z}Y}_{v,w,u}^{4,y,Z}+(1-\chi_{w})\psi_{x,10}Y_{v,w,u}^{4,y,10}+{v\pi}_{w}Y_{1-v,w,u}^{4,y,9}+\gamma_{3,y}Y_{v,w,u}^{3,y,9}-Y_{v,w,u}^{4,y,9}\left( \mu_{v,w}+\alpha_{4,y,9}+\left( 1-v \right)\pi_{w}+\phi_{9,w} \right)$$

*MSM dropped out of ART:*

$$\frac{d}{dt}\left( Y_{v,w,u}^{1,y,10} \right)={\sum_{Z=5}^{Z=9} {\phi_{Z,w}Y}_{v,w,u}^{1,y,Z}+v\pi}_{w}Y_{1-v,w,u}^{1,y,10}+\theta_{y}f_{x,y}\phi_{5,w}A_{v,w,u}^{5}-Y_{v,w,u}^{1,y,10}\left( \gamma_{1,y}+\mu_{v,w}+\alpha_{1,y,10}+\left( 1-v \right)\pi_{w}+\psi_{1,10}+\zeta\right)$$

$$\frac{d}{dt}\left( Y_{v,w,u}^{x,y,10} \right)=\sum_{Z=5}^{Z=9} {\phi_{Z,w}Y}_{v,w,u}^{x,y,Z}{+v\pi}_{w}Y_{1-v,w,u}^{x,y,10}{+\theta}_{y}f_{x,y}\phi_{5,w}A_{v,w,u}^{5}+\gamma_{x-1,y}Y_{v,w,u}^{x-1,y,10}-Y_{v,w,u}^{x,y,10}\left( \gamma_{x,y}+\mu_{v,w}+\alpha_{x,y,10}+\left( 1-v \right)\pi_{w}+\psi_{x,10}+\zeta\right);x\in\left\{ 2,3 \right\}$$

$$\frac{d}{dt}\left( Y_{v,w,u}^{4,y,10} \right)=\sum_{Z=5}^{Z=9} {\phi_{Z,w}Y}_{v,w,u}^{4,y,Z}+{{v\pi}_{w}Y_{1-v,w,u}^{4,y,10}+\theta}_{y}f_{x,y}\phi_{5,w}A_{v,w,u}^{5}+\gamma_{3,y}Y_{v,w,u}^{3,y,10}-Y_{v,w,u}^{4,y,10}\left( \mu_{v,w}+\alpha_{4,y,10}+\left( 1-v \right)\pi_{w}+\psi_{4,10}+\zeta\right)$$

*Force of infection*

Not on PrEP:

$$\lambda_{v,w,u,1}=r_{w}\left( -\ln\left( 1-\left( 1-\left( {\prod_{j=1}^{j=3} \prod_{v^{'}=0}^{v^{'}=1} \prod_{w^{'}=0}^{w^{'}=1} \prod_{u^{'}=0}^{u^{'}=1} \left( \frac{\sum_{z=0}^{z=2} \left( X_{v^{'},w^{'},u^{'}}^{z} \right)}{N_{v^{'},w^{'},u^{'}}}+\frac{\sum_{z=0}^{z=4} \left( A_{v^{'},w^{'},u^{'}}^{z} \right)}{N_{v^{'},w^{'},u^{'}}}\left( 1-d_{1}\beta\left( 1-e_{c}s_{c,ww^{'},uu^{'},j} \right)\left( 1-e_{n}s_{n,w} \right) \right)^{n_{j}}+\sum_{y=1}^{y=4} \left( {\frac{\sum_{x=1}^{x=3} \left( \sum_{z=0}^{z=4} \left( Y_{v^{'},w^{'},u^{'}}^{x,y,z} \right)+Y_{v^{'},w^{'},u^{'}}^{x,y,9}+Y_{v^{'},w^{'},u^{'}}^{x,y,10} \right)}{N_{v^{'},w^{'},u^{'}}}\left( 1-h_{y}\beta\left( 1-e_{c}s_{c,ww^{'},uu^{'},j} \right)\left( 1-e_{n}s_{n,w} \right) \right)}^{n_{j}} \right)+\sum_{y=1}^{y=4} \left( {\frac{\left( \sum_{z=0}^{z=4} \left( Y_{v^{'},w^{'},u^{'}}^{4,y,z} \right)+Y_{v^{'},w^{'},u^{'}}^{4,y,9}+Y_{v^{'},w^{'},u^{'}}^{4,y,10} \right)}{N_{v^{'},w^{'},u^{'}}}\left( 1-d_{2}h_{y}\beta\left( 1-e_{c}s_{c,ww^{'},uu^{'},j} \right)\left( 1-e_{n}s_{n} \right) \right)}^{n_{j}} \right)+\frac{A_{v^{'},w^{'},u^{'}}^{5}}{N_{v^{'},w^{'},u^{'}}}\left( 1-d_{3}\beta\left( 1-e_{c}s_{c,ww^{'},uu^{'},j} \right)\left( 1-e_{n}s_{n,w} \right) \right)^{n_{j}}+\sum_{y=1}^{y=4} \left( {\frac{\sum_{x=1}^{x=3} \left( Y_{v^{'},w^{'},u^{'}}^{x,y,5} \right)}{N_{v^{'},w^{'},u^{'}}}\left( 1-d_{4}h_{y}\beta\left( 1-e_{c}s_{c,ww^{'},uu^{'},j} \right)\left( 1-e_{n}s_{n,w} \right) \right)}^{n_{j}} \right)+\sum_{y=1}^{y=4} \left( {\frac{\left( Y_{v^{'},w^{'},u^{'}}^{4,y,5} \right)}{N_{v^{'},w^{'},u^{'}}}\left( 1-d_{5}h_{y}\beta\left( 1-e_{c}s_{c,ww^{'},uu^{'},j} \right)\left( 1-e_{n}s_{n,w} \right) \right)}^{n_{j}} \right)+\sum_{y=1}^{y=4} \left( {\frac{\sum_{x=1}^{x=4} \sum_{z=6}^{z=8} \left( Y_{v^{'},w^{'},u^{'}}^{x,y,z} \right)}{N_{v^{'},w^{'},u^{'}}}\left( 1-d_{6}h_{y}\beta\left( 1-e_{c}s_{c,ww^{'},uu^{'},j} \right)\left( 1-e_{n}s_{n,w} \right) \right)}^{n_{j}} \right) \right)}^{\rho_{vwu,v^{'}w^{'}u^{'},j}c_{v,w,u,j}} \right) \right) \right) \right)$$

On daily oral TDF/FTC PrEP:

$$\lambda_{v,w,u,2}=r_{w}\left( -\ln\left( 1-\left( 1-\left( {\prod_{j=1}^{j=3} \prod_{v^{'}=0}^{v^{'}=1} \prod_{w^{'}=0}^{w^{'}=1} \prod_{u^{'}=0}^{u^{'}=1} \left( \frac{\sum_{z=0}^{z=2} \left( X_{v^{'},w^{'},u^{'}}^{z} \right)}{N_{v^{'},w^{'},u^{'}}}+\frac{\sum_{z=0}^{z=4} \left( A_{v^{'},w^{'},u^{'}}^{z} \right)}{N_{v^{'},w^{'},u^{'}}}\left( 1-d_{1}\beta\left( 1-e_{c}s_{c,ww^{'},uu^{'},j} \right)\left( 1-e_{n}s_{n} \right)\left( 1-\left( e_{ph}s_{ph,v,w}+e_{pm}s_{pm,v,w}(1-s_{ph,v,w}) \right) \right) \right)^{n_{j}}+\sum_{y=1}^{y=4} \left( {\frac{\sum_{x=1}^{x=3} \left( \sum_{z=0}^{z=4} \left( Y_{v^{'},w^{'},u^{'}}^{x,y,z} \right)+Y_{v^{'},w^{'},u^{'}}^{x,y,9}+Y_{v^{'},w^{'},u^{'}}^{x,y,10} \right)}{N_{v^{'},w^{'},u^{'}}}\left( 1-h_{y}\beta\left( 1-e_{c}s_{c,ww^{'},uu^{'},j} \right)\left( 1-e_{n}s_{n,w} \right)\left( 1-\left( e_{ph}s_{ph,v,w}+e_{pm}s_{pm,v,w}(1-s_{ph,v,w}) \right) \right) \right)}^{n_{j}} \right)+\sum_{y=1}^{y=4} \left( {\frac{\left( \sum_{z=0}^{z=4} \left( Y_{v',w'}^{4,y,z} \right)+Y_{v^{'},w^{'},u^{'}}^{4,y,9}+Y_{v^{'},w^{'},u^{'}}^{4,y,10} \right)}{N_{v^{'},w^{'},u^{'}}}\left( 1-d_{2}h_{y}\beta\left( 1-e_{c}s_{c,ww^{'},uu^{'},j} \right)\left( 1-e_{n}s_{n,w} \right)\left( 1-\left( e_{ph}s_{ph,v,w}+e_{pm}s_{pm,v,w}(1-s_{ph,v,w}) \right) \right) \right)}^{n_{j}} \right)+\frac{A_{v^{'},w^{'},u^{'}}^{5}}{N_{v^{'},w^{'},u^{'}}}\left( 1-d_{3}\beta\left( 1-e_{c}s_{c,ww^{'},uu^{'},j} \right)\left( 1-e_{n}s_{n,w} \right)\left( 1-\left( e_{ph}s_{ph,v,w}+e_{pm}s_{pm,v,w}(1-s_{ph,v,w}) \right) \right) \right)^{n_{j}}+\sum_{y=1}^{y=4} \left( {\frac{\sum_{x=1}^{x=3} \left( Y_{v^{'},w^{'},u^{'}}^{x,y,5} \right)}{N_{v^{'},w^{'},u^{'}}}\left( 1-d_{4}h_{y}\beta\left( 1-e_{c}s_{c,ww^{'},uu^{'},j} \right)\left( 1-e_{n}s_{n,w} \right)\left( 1-\left( e_{ph}s_{ph,v,w}+e_{pm}s_{pm,v,w}(1-s_{ph,v,w}) \right) \right) \right)}^{n_{j}} \right)+\sum_{y=1}^{y=4} \left( {\frac{\left( Y_{v^{'},w^{'},u^{'}}^{4,y,5} \right)}{N_{v^{'},w^{'},u^{'}}}\left( 1-d_{5}h_{y}\beta\left( 1-e_{c}s_{c,ww^{'},uu^{'},j} \right)\left( 1-e_{n}s_{n,w} \right)\left( 1-\left( e_{ph}s_{ph,v,w}+e_{pm}s_{pm,v,w}(1-s_{ph,v,w}) \right) \right) \right)}^{n_{j}} \right)+\sum_{y=1}^{y=4} \left( {\frac{\sum_{x=1}^{x=4} \sum_{z=6}^{z=8} \left( Y_{v^{'},w^{'},u^{'}}^{x,y,z} \right)}{N_{v^{'},w^{'},u^{'}}}\left( 1-d_{6}h_{y}\beta\left( 1-e_{c}s_{c,ww^{'},uu^{'},j} \right)\left( 1-e_{n}s_{n,w} \right)\left( 1-\left( e_{ph}s_{ph,v,w}+e_{pm}s_{pm,v,w}(1-s_{ph,v,w}) \right) \right) \right)}^{n_{j}} \right) \right)}^{\rho_{vwu,v^{'}w^{'}u^{'},j}c_{v,w,u,j}} \right) \right) \right) \right)$$

On long-acting injectable CAB PrEP:

$$\lambda_{v,w,u,2}=r_{w}\left( -\ln\left( 1-\left( 1-\left( {\prod_{j=1}^{j=3} \prod_{v^{'}=0}^{v^{'}=1} \prod_{w^{'}=0}^{w^{'}=1} \prod_{u^{'}=0}^{u^{'}=1} \left( \frac{\sum_{z=0}^{z=2} \left( X_{v^{'},w^{'},u^{'}}^{z} \right)}{N_{v^{'},w^{'},u^{'}}}+\frac{\sum_{z=0}^{z=4} \left( A_{v^{'},w^{'},u^{'}}^{z} \right)}{N_{v^{'},w^{'},u^{'}}}\left( 1-d_{1}\beta\left( 1-e_{c}s_{c,ww^{'},uu^{'},j} \right)\left( 1-e_{n}s_{n} \right)(1-e_{pi}) \right)^{n_{j}}+\sum_{y=1}^{y=4} \left( {\frac{\sum_{x=1}^{x=3} \left( \sum_{z=0}^{z=4} \left( Y_{v^{'},w^{'},u^{'}}^{x,y,z} \right)+Y_{v^{'},w^{'},u^{'}}^{x,y,9}+Y_{v^{'},w^{'},u^{'}}^{x,y,10} \right)}{N_{v^{'},w^{'},u^{'}}}\left( 1-h_{y}\beta\left( 1-e_{c}s_{c,ww^{'},uu^{'},j} \right)\left( 1-e_{n}s_{n,w} \right)(1-e_{pi}) \right)}^{n_{j}} \right)+\sum_{y=1}^{y=4} \left( {\frac{\left( \sum_{z=0}^{z=4} \left( Y_{v',w'}^{4,y,z} \right)+Y_{v^{'},w^{'},u^{'}}^{4,y,9}+Y_{v^{'},w^{'},u^{'}}^{4,y,10} \right)}{N_{v^{'},w^{'},u^{'}}}\left( 1-d_{2}h_{y}\beta\left( 1-e_{c}s_{c,ww^{'},uu^{'},j} \right)\left( 1-e_{n}s_{n,w} \right)(1-e_{pi}) \right)}^{n_{j}} \right)+\frac{A_{v^{'},w^{'},u^{'}}^{5}}{N_{v^{'},w^{'},u^{'}}}\left( 1-d_{3}\beta\left( 1-e_{c}s_{c,ww^{'},uu^{'},j} \right)\left( 1-e_{n}s_{n,w} \right)(1-e_{pi}) \right)^{n_{j}}+\sum_{y=1}^{y=4} \left( {\frac{\sum_{x=1}^{x=3} \left( Y_{v^{'},w^{'},u^{'}}^{x,y,5} \right)}{N_{v^{'},w^{'},u^{'}}}\left( 1-d_{4}h_{y}\beta\left( 1-e_{c}s_{c,ww^{'},uu^{'},j} \right)\left( 1-e_{n}s_{n,w} \right)(1-e_{pi}) \right)}^{n_{j}} \right)+\sum_{y=1}^{y=4} \left( {\frac{\left( Y_{v^{'},w^{'},u^{'}}^{4,y,5} \right)}{N_{v^{'},w^{'},u^{'}}}\left( 1-d_{5}h_{y}\beta\left( 1-e_{c}s_{c,ww^{'},uu^{'},j} \right)\left( 1-e_{n}s_{n,w} \right)(1-e_{pi}) \right)}^{n_{j}} \right)+\sum_{y=1}^{y=4} \left( {\frac{\sum_{x=1}^{x=4} \sum_{z=6}^{z=8} \left( Y_{v^{'},w^{'},u^{'}}^{x,y,z} \right)}{N_{v^{'},w^{'},u^{'}}}\left( 1-d_{6}h_{y}\beta\left( 1-e_{c}s_{c,ww^{'},uu^{'},j} \right)\left( 1-e_{n}s_{n,w} \right)(1-e_{pi}) \right)}^{n_{j}} \right) \right)}^{\rho_{vwu,v^{'}w^{'}u^{'},j}c_{v,w,u,j}} \right) \right) \right) \right)$$

where the total number of MSM partners in age group $v'$ and race group $w'$ is calculated as:

$$N_{v^{'},w^{'},u^{'}}=\sum_{z=0}^{z=2} \left( X_{v^{'},w^{'},u^{'}}^{z} \right)+\sum_{z=0}^{z=5} \left( A_{v^{'},w^{'},u^{'}}^{z} \right)+\sum_{x=1}^{x=4} \sum_{y=1}^{y=4} \sum_{z=0}^{z=10} \left( Y_{v^{'},w^{'},u^{'}}^{x,y,z} \right)$$

Infection risk is estimated for three partner types (j = 1: regular partners, j = 2: casual partners; j = 3: commercial partners). $e_{c}$ is per-sex-act condom efficacy, $s_{c,ww^{'},uu^{'},j}$ is the proportion of sex acts in which a condom is used with partners of type $j$, which varies with the race of both partners and with the PrEP-indication of both partners, $e_{n}$ is per-sex act reduction in HIV acquisition risk due to male circumcision, $s_{n,w}$ is the proportion of MSM who are circumcised, which varies by race, $e_{ph}$ is the per-sex act reduction in HIV acquisition due to daily oral TDF/FTC PrEP use with high adherence (≥4 doses/week), $e_{pm}$ is the per-sex act reduction in HIV acquisition due to daily oral TDF/FTC PrEP use with medium adherence (2-4 doses/week), $s_{ph,v,w}$ is the proportion of men in each age and race group who have high adherence to TDF/FTC PrEP (≥4 doses/week), and $s_{pm,v,w}$ is the proportion of the remaining men on TDF/FTC PrEP who have medium adherence (2-4 doses/week).$e_{pi}$ is the per-sex act reduction in HIV acquisition due to CAB PrEP use. $\beta$ is the average probability of acquiring HIV infection from an anal sex act with an HIV-positive male partner with chronic infection and CD4>200 cells per µL who is not taking ART,$\rho_{vwu,v^{'}w^{'}u^{'},j}$ is, for MSM in age group $v,$ race group $w$ and PrEP-indication group $u$, the proportion of partners of type $j$ who are in age group $v'$ race group $w'$ and PrEP-indication group $u^{'}$(see further details in ‘Mixing’ section below). $c_{v,w,u,j}$ is the average number of new partners per year of type $j$ for MSM in age group $v$, race group $w$ and PrEP-indication group $u$, $n_{j}$ is the average number of sex acts per partnership for a partnership of type $j$, $d_{1}$is the relative infectiousness of those in the acute versus chronic stage of infection, $d_{2}$ is the relative infectiousness of those with CD4<200 cells per µL versus those with chronic infection and CD4>200 cells per µL, $d_{3}, d_{4},d_{5}$ are the relative infectiousness of those on ART with a partially suppressed viral load who have acute infection, chronic infection (CD4>200 cells per µL) or CD4<200 cells per µL, respectively, versus those untreated with chronic infection and CD4>200 cells per µL, $d_{6}$is the relative infectiousness of those on ART with a fully suppressed viral load versus those untreated with chronic infection and CD4>200 cells per µL, and $h_{y}$ is the relative infectiousness of those not fully virally suppressed who have SPVL $y$.

The relative infectiousness of those on ART with a partially suppressed viral load are calculated as follows:

$${d_{3}=d}_{6}+d_{r}\left( d_{1}-d_{6} \right)$$

$${d_{4}=d}_{6}+d_{r}\left( 1-d_{6} \right)$$

$${d_{5}=d}_{6}+d_{r}\left( d_{2}-d_{6} \right)$$

Where $d_{r}$ is the relative level of infectiousness of those partially suppressed, scaled between the level for those fully suppressed ${(d}_{r}=0)$ and those unsuppressed ($d_{r}=1)$.

*Mixing*

$\rho_{vwu,v^{'}w^{'}u^{'},j}$ is calculated from estimates of mixing by age, race and PrEP-indication group, assuming that mixing by each characteristic occurs independently of mixing by the other characteristics:

$$\rho_{vwu,v^{'}w^{'}u^{'},j}= \varepsilon_{a}\varepsilon_{r}\varepsilon_{p}\rho_{vv^{'}}^{A}\rho_{ww^{'}}^{A}\rho_{uu^{'}}^{A}+\varepsilon_{a}\varepsilon_{r}\left( 1-\varepsilon_{p} \right)\rho_{vv^{'}}^{A}\rho_{ww^{'}}^{A}\frac{N_{v^{'}w^{'}u^{'}}c_{v^{'}w^{'}u^{'}j}}{N_{v^{'}w^{'}0}c_{v^{'}w^{'}0j}+N_{v^{'}w^{'}1}c_{v^{'}w^{'}1j}} +\varepsilon_{a}\left( 1-\varepsilon_{r} \right)\varepsilon_{p}\rho_{vv^{'}}^{A}\rho_{uu^{'}}^{A}\frac{N_{v^{'}w^{'}u^{'}}c_{v^{'}w^{'}u^{'}j}}{N_{v^{'}0u^{'}}c_{v^{'}0u^{'}j}+N_{v^{'}1u^{'}}c_{v^{'}1u^{'}j}}+\left( 1-\varepsilon_{a} \right){\varepsilon_{r}\varepsilon}_{p}\rho_{ww^{'}}^{A}\rho_{uu^{'}}^{A}\frac{N_{v^{'}w^{'}u^{'}}c_{v^{'}w^{'}u^{'}j}}{N_{{0w}^{'}u^{'}}c_{{0w}^{'}u^{'}j}+N_{{1w}^{'}u^{'}}c_{{1w}^{'}u^{'}j}} +\varepsilon_{a}\left( 1-\varepsilon_{r} \right)\left( 1-\varepsilon_{p} \right)\rho_{vv^{'}}^{A}\frac{N_{v^{'}w^{'}u^{'}}c_{v^{'}w^{'}u^{'}j}}{N_{v^{'}00}c_{v^{'}00j}+N_{v^{'}10}c_{v^{'}10j}+N_{v^{'}10}c_{v^{'}10j}+N_{v^{'}11}c_{v^{'}11j}}+\left( 1-\varepsilon_{a} \right)\varepsilon_{r}\left( 1-\varepsilon_{p} \right)\rho_{ww^{'}}^{A}\frac{N_{v^{'}w^{'}u^{'}}c_{v^{'}w^{'}u^{'}j}}{N_{{0w}^{'}0}c_{0w^{'}0j}+N_{{1w}^{'}0}c_{1w^{'}0j}+N_{{0w}^{'}1}c_{0w^{'}1j}+N_{1w^{'}1}c_{1w^{'}1j}}+\left( 1-\varepsilon_{a} \right)\left( 1-\varepsilon_{r} \right)\varepsilon_{p}\rho_{uu^{'}}^{A}\frac{N_{v^{'}w^{'}u^{'}}c_{v^{'}w^{'}u^{'}j}}{N_{{00u}^{'}}c_{00u^{'}j}+N_{{10u}^{'}}c_{1{0u}^{'}j}+N_{{01u}^{'}}c_{01u^{'}j}+N_{{11u}^{'}}c_{1{1u}^{'}j}}+\left( 1-\varepsilon_{a} \right)\left( 1-\varepsilon_{r} \right)\left( 1-\varepsilon_{p} \right) \frac{N_{v^{'}w^{'}u^{'}}c_{v^{'}w^{'}u^{'}j}}{N_{000}c_{000j}+N_{100}c_{100j}+N_{010}c_{010j}+N_{110}c_{110j}+N_{001}c_{001j}+N_{101}c_{101j}+N_{011}c_{011j}+N_{111}c_{111j}}$$

where $\varepsilon_{a},\varepsilon_{r}$ and $\varepsilon_{p}$are parameters governing mixing by age, race and PrEP-indication, ranging between 0 (fully proportionate) and 1 (fully assortative, i.e. “like-with-like” mixing). $\rho_{xx^{'}}^{A}$is the assortative mixing matrix, where:

$$\rho_{xx^{'}}^{A}=1 if x=x^{'}$$

$$\rho_{xx^{'}}^{A}=0 if x\neq x^{'}$$

## Model parameters

The values and ranges used for all model parameters, together with data sources and justification, are given in Table S1.

*Initial conditions*

The size of the MSM population in Atlanta in 1979 was estimated from 1980 census data on the total number of men living in Atlanta and more recent published estimates of the proportion of men who have had sex with other men nationwide and by county.^1,2^ The race and age distribution of the MSM population in 1979 was estimated from 1980 and 1990 census data on the race and age distribution of the general male population.

The percentage of MSM meeting the criteria for a PrEP indication (both among the MSM population in 1979 and among new MSM in subsequent years) was based upon data from the 2011 and 2014 NHBS surveys. The US Public Health Service recommends indication for PrEP use for MSM who: have had any male sex partners in the past 6 months, are not in a monogamous partnership with a recently tested, HIV-negative man, and have either a) had any anal sex without condoms in the past 6 months or b) had a bacterial STI diagnosed or reported in the past 6 months.^3^ This was approximated using data available in the NHBS as those who: reported 1) ≥2 male partners AND either condomless anal sex or a bacterial STD in the past 12 months or 2) a main HIV-infected male partner in the past 12 months.^4^

*Demography*

The race and age distribution of new MSM joining the population were estimated from NHBS data and adjusted to ensure that the model fit to NHBS demography. Non-HIV-related death rates were based on CDC data for Georgia state.^5^ Rates of migration into and out of the population (including rates of ceasing to attend NHBS venues including bars, clubs and cafés) were estimated to enable the model to fit to demography data.

*Sexual behaviour*

Age-, race- and PrEP indication-specific numbers of new main, casual and commercial anal sex partners, condom use in different partnerships, changes over time in partner numbers and condom use, and sexual mixing by age and race were estimated from NHBS data for MSM in Atlanta. Note that data from the 2008 NHBS survey was not used, as this population had a very different demographic and epidemiological profile from the other surveys (2004, 2011, 2014).

Numbers of new partners were estimated from NHBS data on total numbers of main and casual anal sex partners, data on numbers of new main and casual sex partners (including those with whom they did not have anal sex), and data on exchange sex partners. Numbers of casual and commercial partners were assumed to decline linearly over time until 2011, remaining constant subsequently, while numbers of main partners were assumed to stay constant over time, all in line with NHBS data for 2004-2014.

Parameters describing mixing by age and race were estimated from 2011 and 2014 NHBS data on the age and race of partners by respondent’s age and race (note that data on partner age-group was only available for MSM aged 18-24 years old). Assuming that the proportion of MSM in each age and race group in the NHBS survey was representative of the wider MSM population and using the total annual number of anal sex partners reported by each group, we used least-squares fitting to identify plausible values for mixing parameters by age and race (on a scale between 0 for fully proportionate (partners chosen at random) to 1 for fully assortative (mixing only with partners in the same group)). This analysis suggested some preference for partners of similar age, and strong preferences for partners of the same race. No data was available on sexual mixing by PrEP indication, so we explored the range of mixing by PrEP indication between fully proportionate and fully assortative (0-1).

Condom use was assumed to be higher in casual than main partnerships, in line with NHBS data. There was insufficient data available to estimate condom use in commercial partnerships, so this was assumed to be similar to condom use in casual partnerships. For main (but not casual or commercial) partnerships, higher condom use was assumed for Black vs. White MSM, and condom use in all partnerships was lower for MSM with vs. without a PrEP indication, in line with NHBS data. Condom use was assumed to decrease over time, with changes in earlier years (1996-2008) based on surveys conducted among MSM at Gay Pride events in Atlanta,^6^ and subsequent declines up to 2014 based on NHBS data.

The number of sex acts per main partnership was estimated from data on frequency of sex acts durations of partnerships from previous studies of US MSM.^7-9^ The number of sex acts per casual and commercial partnership was estimated from previous analysis using this modelling framework to study the HIV epidemic among MSM in Baltimore, obtained when fitting the model to data on partner numbers and the proportion of most recent sex acts which were with main, casual and commercial partners.^10^

*HIV disease progression, transmission probabilities and intervention efficacy*

HIV disease progression rates (including HIV-related death rates by CD4 count, SPVL and HIV treatment status, and CD4 decay rates by SPVL), transmission probabilities and intervention efficacies (for condoms, circumcision, HIV treatment, TDF/FTC PrEP) were obtained from published studies.^11-27^

To account for differences in HIV incidence by race which were not captured by differences in sexual risk behaviour, mixing and access to care, a relative susceptibility term for White vs Black MSM was included and varied between 0.2-1 to enable fitting to race-specific HIV prevalence data.

*CAB effectiveness*

A synthesis estimate of CAB effectiveness versus placebo was calculated by combining the primary effectiveness results from the HPTN 083 trial, comparing CAB to TDF/FTC, with a meta-regression estimate of the effectiveness of TDF/FTC compared to a hypothetical placebo arm in the 083 trial. The meta-regression model, which estimates the association between TDF/FTC adherence (as measured by the proportion of participants with any detectable tenofovir in plasma) and HIV risk reduction, was calibrated using historical randomized, blinded, placebo-controlled trials of TDF/FTC.^28,29^ A random sample of 390 TDF/FTC-arm participants was selected for adherence assessment in 083, and plasma was assessed for TFV at multiple time points for each person. Approximately 86% of tested samples had detectable TFV, which when applied to the meta-regression model yielded an estimated 75% (95% CI: 63-83%) reduction in HIV acquisition risk compared to placebo. The synthesis estimate was taken as the product Relative Risk (CAB vs TDF/FTC) × Relative Risk (TDF/FTC vs Placebo), and the 95% confidence interval was calculated by summing the variances on the log scale (i.e. assuming independence).

Note that this estimate is for overall CAB effectiveness; separate estimates of efficacy and adherence for CAB were not available.

*Intervention behaviour*

The race-specific percentage of MSM who never test for HIV was estimated from NHBS data among those aged >25 years old.

Age- and race-specific HIV testing rates from 2004 onwards were estimated from the proportion of MSM reporting testing for HIV in the last year in the NHBS. Earlier testing rates were estimated for MSM from a national survey conducted in 1996,^30^ with HIV testing rates assumed to have increased linearly between 1996 and 2004. The model input proportion testing in the last year was reduced by 50-60% compared with the data values in order to fit the model to state-level diagnosis data, as in previous analysis for Baltimore.^19^ The proportion testing was converted into a rate of testing at least once a year for use in the model.

The race-specific proportion of MSM living with HIV and not on PrEP who link to HIV care straight away after diagnosis was estimated at the state level from surveillance data from the Georgia Department of Health.^31,32^ The proportion linking to HIV care straight away was assumed to increase over time, in line with national CDC data.^33,34^ Those on PrEP at the time of diagnosis were all assumed to link to HIV care straight away. Subsequent rates of linkage for those not linking straight away were varied to fit data on the proportion of MSM living with HIV receiving HIV care. Rates of dropout from care relative to ART dropout rates, and for Black MSM compared with White MSM, were estimated from US studies.^35-40^

ART initiation among those diagnosed and in care followed historical US ART guidelines - which restricted ART initiation by CD4 count - from 1996 to 2012, when restrictions on ART initiation were removed. Initiation rates reflected recommended CD4 testing frequency and ART acceptance and were varied to fit ART coverage data. ART initiation rates due to developing AIDS symptoms were based on incidence of AIDS-defining illness (following CDC definitions) by CD4 count among ART cohorts,^41,42^ assuming that those developing AIDS-defining illness would seek and initiate treatment promptly.

Race-specific levels of adherence to ART were based on US cohort and surveillance data on the proportion of those on ART who achieved viral suppression.^35,43-47^

Rates of ART dropout, by race and duration on ART, and rates of re-enrolment into HIV care following ART dropout, were estimated from US cohort studies.^35-39,48^

Race-specific levels of circumcision among MSM were estimated from NHBS data.

Age-and race-specific rates of PrEP acceptance, adherence and dropout were estimated from US PrEP Demo project data for all participants. PrEP dropout rates were calculated from the % in each group retained at the end of the study. Rates of being offered PrEP were assumed to be proportional to HIV testing rates, and to increase linearly over time from 2012-2021, with PrEP offer rates in 2021 exceeding HIV testing rates (by 2-6-fold) in order to achieve observed PrEP coverage levels. For the sensitivity analysis, race-specific levels of PrEP adherence were estimated from measures of adherence to TDF/FTC for a subset of HPTN 083 participants in the US.^49^

## Parameter table

**Table S1. Parameters used in the HIV transmission model, with source and justification**

| **Symbol** | **Parameter** | **Range of values^a^** | **Source/justification** |
| --- | --- | --- | --- |
| **INITIAL CONDITIONS** | | | |
| $N_{0}$ | Initial size of MSM population (1979) | 21500-30000 | 740,030 men aged 18+ in Fulton county in the 1980 census; Lower bound: Purcell et al. 2012^2^ estimate % of US men had same-sex behaviour last 12 months 2.9%. Upper bound: 4%; Grey et al 2016^1^ estimate 5.4% of men in Atlanta had sex with another man in the last 5 years, adjusted for ratio for last year: last 5 years from Purcell et al. |
|  | Percentage of MSM who are Black in 1979 | 10-23 | Upper bound: overall Atlanta population 1980 census. Lower bound used to improve fitting to race data in later years. |
|  | Percentage of Black MSM aged 18-24 in 1979 | 22-32 | 1990 census estimate (27%) ±5 percentage points (pp) |
|  | Percentage of White MSM aged 18-24 in 1979 | 6-16 | 1990 census estimate (11%) ±5 percentage points (pp) |
| $m_{0,0,high}$ | Percentage of Black 18-24 year old MSM meeting criteria for a PrEP indication in 1979 and among new incoming MSM | 14-76 | NHBS data 2011-2014 (highest and lowest from 95% CI); PrEP indication if reported 1) ≥2 male partners AND (condomless anal sex OR bacterial STD) in the past 12 months or 2) main HIV-infected male partner in the past 12 months |
| $m_{0,1,high}$ | Percentage of White 18-24 year old MSM meeting criteria for a PrEP indication in 1979 and among new incoming MSM | 40-61 | NHBS data 2011-2014 (highest and lowest from 95% CI); PrEP indication if reported 1) ≥2 male partners AND (condomless anal sex OR bacterial STD) in the past 12 months or 2) main HIV-infected male partner in the past 12 months |
| $m_{1,0,high}$ | Percentage of Black 25+ year old MSM meeting criteria for a PrEP indication in 1979 and among new incoming MSM | 13-67 | NHBS data 2011-2014 (highest and lowest from 95% CI); PrEP indication if reported 1) ≥2 male partners AND (condomless anal sex OR bacterial STD) in the past 12 months or 2) main HIV-infected male partner in the past 12 months |
| $m_{1,1,high}$ | Percentage of Black 25+ year old MSM meeting criteria for a PrEP indication in 1979 and among new incoming MSM | 37-61 | NHBS data 2011-2014 (highest and lowest from 95% CI); PrEP indication if reported 1) ≥2 male partners AND (condomless anal sex OR bacterial STD) in the past 12 months or 2) main HIV-infected male partner in the past 12 months |
|  | HIV prevalence Black MSM 1979 (%) | 0.1-2 | Assumption |
|  | HIV prevalence White MSM 1979 (%) | 0.1-1 | Assumption |
| ***Demography*** | |  |  |
| $\Gamma$ | Rate at which new MSM join the sexually active MSM population (number per year) | 3000-5500 | estimate – fitting to NHBS demography |
| $m_{black}$ | Percentage of new incoming MSM who are Black, 2014^b^ | 45-75 | Lower bound: % of current MSM who are Black NHBS 2011; Upper bound set to fit data on race (% of current MSM who are Black NHBS 2014: 62%) |
| $m_{black}\_inc$ | Annual increase in percentage of new incoming MSM who are Black, 1979-2014 | 0.2-1.5 | Estimate – fitting to NHBS demography |
| $m_{young,0}$ | Percentage of new incoming Black MSM who are aged 18-24 years old | 51-70 | % of Black MSM in NHBS who say they entered sexually active Atlanta MSM population aged <25 – 2014 NHBS |
| $m_{young,1}$ | Percentage of new incoming White MSM who are aged 18-24 years old | 30-56 | % of White MSM in NHBS who say they entered sexually active Atlanta MSM population aged <25 – 2014 NHBS; lower bound reduced to fit data on age |
| $\pi_{0}$ | rate of moving from 18-24 year old age group to >24 year old age group, Black MSM, per year | 0.17 (fixed) | Mean age at joining the local MSM population in NHBS 2011 for 18-24 yr old MSM ~16 yrs old (95% CI 15-17), in NHBS 2014 17.5 yrs old (17-18) |
| $\pi_{1}$ | rate of moving from 18-24 year old age group to >24 year old age group, White MSM, per year | 0.17-0.25 | Mean age at joining the local MSM population in NHBS 2011 for 18-24 yr old MSM ~19 yrs old (95% CI 18-20), in NHBS 2014 ~19.5 yrs old (18-21). Assume they spend 4-6 years in 18-24 yr old age group. |
| $\mu_{0,0}$ | Non-HIV related death rate, 18-24 year old Black men, per year | 0.0014-0.0018 | CDC WONDER database data for Georgia^5^; data for 15-24 years olds |
| $\mu_{1,0}$ | Non-HIV related death/leaving rate, >24 year old Black men, per year | 0.037-0.11 | CDC WONDER database data for Georgia^5^: average death rate over ages 25-64 years old, add on 1/29 (double current duration as an MSM) to upper bound, additionally assume extra rate of  ceasing to attend NHBS venues |
| $\mu_{0,1}$ | Non-HIV related death rate, 18-24 year old White men, per year | 0.0011-0.0015 | CDC WONDER database data for Georgia^5^; data for 15-24 years olds |
| $\mu_{1,1}$ | Non-HIV related death/leaving rate, >24 year old White men, per year | 0.035-0.1 | CDC WONDER database data for Georgia^5^: average death rate over ages 25-64 years old, add on 1/43 (double current duration as an MSM) to upper bound, additionally assume extra rate of  ceasing to attend NHBS venues |
| ***Sexual behaviour*** | | | |
| $n_{1}$ | Number of anal sex acts per main partnership | 40-470 | 48.2-85.1 sex episodes/year with main partners ^7^, partnerships last 3.5-5.5 years ^8,9^, but assume some are shorter (~1 year) |
| $n_{2}$ | Number of anal sex acts per casual partnership | 1-40 | In line with previous fitted estimates for MSM in Baltimore^10^ |
| $n_{3}$ | Number of anal sex acts per commercial partnership | 1-40 | In line with previous fitted estimates for MSM in Baltimore^10^ |
| $c_{0,0,0,1}$ | Number of new main partners per year, 18-24 year old Black MSM without a PrEP indication | 0.29-0.65 | NHBS 2014 - number of main male anal sex partners in the last 12 months multiplied by the proportion of all main partners in the past 12 months who were new partners. |
| $c_{0,0,0,2}$ | Number of new casual partners per year, 18-24 year old Black MSM without a PrEP indication 2011 onwards^b^ | 0.4-1.45 | NHBS 2014 - number of casual male anal sex partners in the last 12 months multiplied by the proportion of all casual partners in the past 12 months who were new partners, subtracting the number of new commercial partners |
| $c_{0,0,0,3}$ | Number of new commercial partners per year, 18-24 year old Black MSM without a PrEP indication 2011 onwards^b^ | 0-0.06 | NHBS 2014 - number of exchange male sex partners in the last 12 months multiplied by the proportion of all casual partners in the past 12 months who were new partners and multiplied by the proportion of casual partners with whom they had anal sex |
| $c_{0,0,1,1}$ | Number of new main partners per year, 18-24 year old Black MSM with a PrEP indication | 0.65-1.12 | NHBS 2014 - number of main male anal sex partners in the last 12 months multiplied by the proportion of all main partners in the past 12 months who were new partners. |
| $c_{0,0,1,2}$ | Number of new casual partners per year, 18-24 year old Black MSM with a PrEP indication 2011 onwards^b^ | 1.5-2.93 | NHBS 2014 - number of casual male anal sex partners in the last 12 months multiplied by the proportion of all casual partners in the past 12 months who were new partners, subtracting the number of new commercial partners |
| $c_{0,0,1,3}$ | Number of new commercial partners per year, 18-24 year old Black MSM with a PrEP indication 2011 onwards^b^ | 0.0005-0.45 | NHBS 2014 - number of exchange male sex partners in the last 12 months multiplied by the proportion of all casual partners in the past 12 months who were new partners and multiplied by the proportion of casual partners with whom they had anal sex |
| $c_{1,0,0,1}$ | Number of new main partners per year, >24 year old Black MSM without a PrEP indication | 0.41-0.66 | NHBS 2014 - number of main male anal sex partners in the last 12 months multiplied by the proportion of all main partners in the past 12 months who were new partners. |
| $c_{1,0,0,2}$ | Number of new casual partners per year, >24 year old Black MSM without a PrEP indication 2011 onwards^b^ | 0.52-0.93 | NHBS 2014 - number of casual male anal sex partners in the last 12 months multiplied by the proportion of all casual partners in the past 12 months who were new partners, subtracting the number of new commercial partners |
| $c_{1,0,0,3}$ | Number of new commercial partners per year, >24 year old Black MSM without a PrEP indication 2011 onwards^b^ | 0.01-0.18 | NHBS 2014 - number of exchange male sex partners in the last 12 months multiplied by the proportion of all casual partners in the past 12 months who were new partners and multiplied by the proportion of casual partners with whom they had anal sex |
| $c_{1,0,1,1}$ | Number of new main partners per year, >24 year old Black MSM with a PrEP indication | 0.41-0.62 | NHBS 2014 - number of main male anal sex partners in the last 12 months multiplied by the proportion of all main partners in the past 12 months who were new partners. |
| $c_{1,0,1,2}$ | Number of new casual partners per year, >24 year old Black MSM with a PrEP indication 2011 onwards^b^ | 2.78-5.14 | NHBS 2014 - number of casual male anal sex partners in the last 12 months multiplied by the proportion of all casual partners in the past 12 months who were new partners, subtracting the number of new commercial partners |
| $c_{1,0,1,3}$ | Number of new commercial partners per year, >24 year old Black MSM with a PrEP indication 2011 onwards^b^ | 0.19-1.67 | NHBS 2014 - number of exchange male sex partners in the last 12 months multiplied by the proportion of all casual partners in the past 12 months who were new partners and multiplied by the proportion of casual partners with whom they had anal sex |
| $c_{0,1,0,1}$ | Number of new main partners per year, 18-24 year old White MSM without a PrEP indication | 0.7-2.24 | NHBS 2014 - number of main male anal sex partners in the last 12 months multiplied by the proportion of all main partners in the past 12 months who were new partners. |
| $c_{0,1,0,2}$ | Number of new casual partners per year, 18-24 year old White MSM without a PrEP indication 2011 onwards^b^ | 0-1.38 | NHBS 2014 - number of casual male anal sex partners in the last 12 months multiplied by the proportion of all casual partners in the past 12 months who were new partners, subtracting the number of new commercial partners |
| $c_{0,1,0,3}$ | Number of new commercial partners per year, 18-24 year old White MSM without a PrEP indication 2011 onwards^b^ | 0-0.07 | NHBS 2014 - number of exchange male sex partners in the last 12 months multiplied by the proportion of all casual partners in the past 12 months who were new partners and multiplied by the proportion of casual partners with whom they had anal sex |
| $c_{0,1,1,1}$ | Number of new main partners per year, 18-24 year old White MSM with a PrEP indication | 0.18-0.62 | NHBS 2014 - number of main male anal sex partners in the last 12 months multiplied by the proportion of all main partners in the past 12 months who were new partners. |
| $c_{0,1,1,2}$ | Number of new casual partners per year, 18-24 year old White MSM with a PrEP indication 2011 onwards^b^ | 0-1.45 | NHBS 2014 - number of casual male anal sex partners in the last 12 months multiplied by the proportion of all casual partners in the past 12 months who were new partners, subtracting the number of new commercial partners |
| $c_{0,1,1,3}$ | Number of new commercial partners per year, 18-24 year old White MSM with a PrEP indication 2011 onwards^b^ | 0-0.19 | NHBS 2014 - number of exchange male sex partners in the last 12 months multiplied by the proportion of all casual partners in the past 12 months who were new partners and multiplied by the proportion of casual partners with whom they had anal sex |
| $c_{1,1,0,1}$ | Number of new main partners per year, >24 year old White MSM without a PrEP indication | 0.2-0.43 | NHBS 2014 - number of main male anal sex partners in the last 12 months multiplied by the proportion of all main partners in the past 12 months who were new partners. |
| $c_{1,1,0,2}$ | Number of new casual partners per year, >24 year old White MSM without a PrEP indication 2011 onwards^b^ | 0.78-2.92 | NHBS 2014 - number of casual male anal sex partners in the last 12 months multiplied by the proportion of all casual partners in the past 12 months who were new partners, subtracting the number of new commercial partners |
| $c_{1,1,0,3}$ | Number of new commercial partners per year, >24 year old White MSM without a PrEP indication 2011 onwards^b^ | 0-0.06 | NHBS 2014 - number of exchange male sex partners in the last 12 months multiplied by the proportion of all casual partners in the past 12 months who were new partners and multiplied by the proportion of casual partners with whom they had anal sex |
| $c_{1,1,1,1}$ | Number of new main partners per year, >24 year old White MSM with a PrEP indication | 0.21-0.44 | NHBS 2014 - number of main male anal sex partners in the last 12 months multiplied by the proportion of all main partners in the past 12 months who were new partners. |
| $c_{1,1,1,2}$ | Number of new casual partners per year, >24 year old White MSM with a PrEP indication 2011 onwards^b^ | 3.69-16.4 | NHBS 2014 - number of casual male anal sex partners in the last 12 months multiplied by the proportion of all casual partners in the past 12 months who were new partners, subtracting the number of new commercial partners |
| $c_{1,1,1,3}$ | Number of new commercial partners per year, >24 year old White MSM with a PrEP indication 2011 onwards^b^ | 0-2.52 | NHBS 2014 - number of exchange male sex partners in the last 12 months multiplied by the proportion of all casual partners in the past 12 months who were new partners and multiplied by the proportion of casual partners with whom they had anal sex |
| Partner_number_decline_low | absolute decline per year in the number of new casual or commercial partners, for MSM without a PrEP indication | 0.51-0.71 | From trends in NHBS data on number of commercial and causal partners 2008-2014 for MSM without a PrEP indication, and trends in number of commercial and casual partners 2004-2014 for all MSM |
| Partner_number_decline_high | absolute decline per year in the number of new casual or commercial partners, for MSM with a PrEP indication | 0.92-1.12 | From trends in NHBS data on number of commercial and causal partners 2008-2014 for MSM with a PrEP indication, and trends in number of commercial and casual partners 2004-2014 for all MSM |
| $\varepsilon_{a}$ | Mixing parameter for mixing by age scaled between fully proportionate (0) and fully assortative (1) | 0.32-0.62 | 0.53 estimated from NHBS 2011 data on last partner, and 0.41 from 2014 data on last partner; range takes into account uncertainty due to incomplete data on age of last partner |
| $\varepsilon_{r}$ | Mixing parameter for mixing by race scaled between fully proportionate (0) and fully assortative (1) | 0.69-0.79 | 0.75 estimated from NHBS 2011 data on last partner and 0.73 from NHBS 2014 data on last partner |
| $\varepsilon_{p}$ | Mixing parameter for mixing by PrEP indication scaled between fully proportionate (0) and fully assortative (1) | 0-1 | No data; full range explored |
| $s_{c,1,0,0}$ | Percentage of sex acts in which a condom is used, main partnerships where both partners are Black and one or both partners do not have a PrEP indication, 2014 onwards^b^ | 41-69 | condom use last sex act reported by Black MSM without a PrEP indication with main partners NHBS 2014 |
| $s_{c,1,1,0}$ | Percentage of sex acts in which a condom is used, main partnerships where one or both partners are White and one or both partners do not have a PrEP indication, 2014 onwards^b^ | 21-57 | condom use last sex act reported by White MSM without a PrEP indication with main partners NHBS 2014 |
| $s_{c,2,0,0}{,s}_{c,2,1,0}$ | Percentage of sex acts in which a condom is used, casual partnerships (any race partner) where one or both partners do not have a PrEP indication, 2014 onwards^b^ | 40-91 | condom use last sex act reported in casual partnerships by MSM without a PrEP indication NHBS 2014 – range from values for Black and White MSM |
| $s_{c,3,0,0},s_{c,3,1,0}$ | Percentage of sex acts in which a condom is used, commercial partnerships (any race partner) where one or both partners do not have a PrEP indication, 2014 onwards^b^ | 40-91 | Assumed to be the same as casual |
| $s_{c,1,0,1}$ | Percentage of sex acts in which a condom is used, main partnerships where both partners are Black and both partners have a PrEP indication, 2014 onwards^b^ | 18-56 | condom use last sex act reported by Black MSM with a PrEP indication with main partners NHBS 2014 |
| $s_{c,1,1,1}$ | Percentage of sex acts in which a condom is used, main partnerships where one or both partners are White and both partners have a PrEP indication, 2014 onwards^b^ | 3-31 | condom use last sex act reported by White MSM with a PrEP indication with main partners NHBS 2014 |
| $s_{c,2,0,1}{,s}_{c,2,1,1}$ | Percentage of sex acts in which a condom is used, casual partnerships (any race partner) where both partners have a PrEP indication, 2014 onwards^b^ | 17-60 | condom use last sex act reported in casual partnerships by MSM with a PrEP indication NHBS 2014 – range from values for Black and White MSM |
| $s_{c,3,0,1},s_{c,3,1,1}$ | Percentage of sex acts in which a condom is used, commercial partnerships (any race partner) where both partners have a PrEP indication, 2014 onwards^b^ | 17-60 | Assumed to be the same as casual |
| Condom_change_1 | Yearly change in % of sex acts in which condoms are used, all partnerships 1996-2008 | -0.94 to -0.31 | From study by Kalichman et al^6^, decline between 1997 and 2005, ±50% |
| Condom_change_2 | Yearly change in % of sex acts in which condoms are used, all partnerships between 2008 and 2014 | -4.5 to -0.1 | From trend in condom use at last sex act NHBS 2011-2014, taking into account variation across race and PrEP-indication groups, and across main and casual partners. |
| ***HIV disease progression*** | |  |  |
| $1/\gamma_{a}$ | Average duration of acute infection, months | 2-6 | Systematic review and analysis of data from Uganda^11,12^ |
| $\alpha_{0,0,z}$ | HIV-related death rate for those with acute HIV infection, per year | 0 (fixed) | assumption |
| $\alpha_{1,y,0}$ $\alpha_{1,y,1}$ $\alpha_{1,y,2}$ $\alpha_{1,y,3}$ $\alpha_{1,y,4}$ $\alpha_{1,y,9}$ $\alpha_{1,y,10}$ | HIV-related death rate for those with CD4>500, off ART, per year | 0.0009-0.0054 | aged 25-44 in the European CASCADE cohort ^13^; general population death rate subtracted |
| $\alpha_{2,y,0}$ $\alpha_{2,y,1}$ $\alpha_{2,y,2}$ $\alpha_{2,y,3}$ $\alpha_{2,y,4}$ $\alpha_{2,y,9}$ $\alpha_{2,y,10}$ | HIV-related death rate for those with CD4 350-500, off ART, per year | 0.0009-0.0069 | aged 25-44 in the European CASCADE cohort ^13^; general population death rate subtracted |
| $\alpha_{3,y,0}$ $\alpha_{3,y,1}$ $\alpha_{3,y,2}$ $\alpha_{3,y,3}$ $\alpha_{3,y,4}$ $\alpha_{3,y,9}$ $\alpha_{3,y,10}$ | HIV related death rate for those with CD4 200-350, off ART, per year | 0.0045-0.0135 | aged 25-44 in the European CASCADE cohort ^13^; general population death rate subtracted |
| 1/$\alpha_{4,1,1}$ | Inverse of HIV-related death rate for those with CD4<200, SPVL<4.0, off ART (years) | 3.28-12.87 | Netherlands ATHENA cohort ^14^ |
| 1/$\alpha_{4,2,1}$ | Inverse of HIV-related death rate for those with CD4<200, SPVL 4.0-4.5, off ART (years) | 1.43-6.09 | Netherlands ATHENA cohort ^14^ |
| 1/$\alpha_{4,3,1}$ | Inverse of HIV-related death rate for those with CD4<200, SPVL 4,5-5,0, off ART (years) | 4.41-23.64 | Netherlands ATHENA cohort ^14^ |
| 1/$\alpha_{4,4,1}$ | Inverse of HIV-related death rate for those with CD4<200, SPVL>5.0, off ART (years) | 1.32-3.59 | Netherlands ATHENA cohort ^14^ |
| $\alpha_{1,y,5}$,  ,  ,  ,$\alpha_{2,y,8}$ | HIV-related mortality for those with CD4>500 or CD4 350-500 at start of treatment, for 1^st^ , 2^nd^ and subsequent years on ART, per year | 0-0.003 | European and North American cohorts; from probabilities for those with CD4>350 ^15^; general population death rate subtracted ^50^ |
| $a_{1}$ | Relative mortality of those with CD4 200-350 vs CD4>350 at start of treatment, 1^st^ year on ART | 1.2-2.8 | European and North American cohorts^16^ |
| $a_{2}$ | Relative mortality of those with CD4 200-350 vs CD4>350 at start of treatment, 2^nd^ year on ART | 1-2.2 | European and North American cohorts ^16^ Upper limit reduced to give main estimate as midpoint |
| $a_{3}$ | Relative mortality of those with CD4 200-350 vs CD4>350 at start of treatment, 3^rd^ year + on ART | 1-1.4 | European and North American cohorts ^16^ Upper limit reduced to give main estimate as midpoint |
| $a_{4}$ | Relative mortality of those with CD4 <200 vs CD4>350 at start of treatment, 1^st^ year on ART | 1.8-5.2 | European and North American cohorts ^16^ Main estimate and lower bound: CD4 100-199; upper bound from those with CD4 25-49 |
| $a_{5}$ | Relative mortality of those with CD4 <200 vs CD4>350 at start of treatment, 2^nd^ year on ART | 1.3-6.2 | European and North American cohorts ^16^ Main estimate and lower bound: CD4 100-199; upper bound from those with CD4 25-49 |
| $a_{6}$ | Relative mortality of those with CD4 <200 vs CD4>350 at start of treatment, 3^rd^ year + on ART | 1-3.2 | European and North American cohorts ^16^ Main estimate and lower bound: CD4 100-199; upper bound from those with CD4 50-99 |
| $b_{1}$ | Relative mortality of those with AIDS before ART initiation vs without, 1^st^ year on ART | 3.0-4.8 | European and North American cohorts ^16^ |
| $b_{2},b_{3}$ | Relative mortality of those with AIDS before ART initiation vs without, 2^nd^ , 3^rd^ + years on ART | 1.4-2.6 | European and North American cohorts ^16^ |
| $k_{4}$ | Percentage of those starting ART with CD4<200 who have a prior AIDS diagnosis | 40-60 | US data ^51^ |
| $\theta_{2}$ | Percentage of HIV-positive MSM with a SPVL 4.0-4.5 | 25 (fixed) | Netherlands ATHENA cohort ^14^; US MSM (MACS cohort)^17,18^ |
| $\theta_{3}$ | Percentage of HIV-positive MSM with a SPVL 4.5-5.0 | 25-40 | Netherlands ATHENA cohort ^14^; US MSM (MACS cohort)^17,18^ |
| $\theta_{4}$ | Percentage of HIV-positive MSM with a SPVL >5.0 | 10-25 | Netherlands ATHENA cohort ^14^; US MSM (MACS cohort)^18^ |
| ${1/\gamma}_{1,1}$ | Average duration spent with CD4>500 cells per µL, for those with SPVL <4.0 (years) | 4.56-6.37 | Netherlands ATHENA cohort ^14^ |
| ${1/\gamma}_{2,1}$ | Average duration spent with CD4 350-500, for those with SPVL <4.0 (years) | 2.98-4.53 | Netherlands ATHENA cohort ^14^ |
| ${1/\gamma}_{3,1}$ | Average duration spent with CD4 200-350, for those with SPVL <4.0 (years) | 5.04-13.69 | Netherlands ATHENA cohort ^14^ |
| ${1/\gamma}_{1,2}$ | Average duration spent with CD4>500, for those with SPVL 4.0-4.5 (years) | 2.68-3.64 | Netherlands ATHENA cohort ^14^ |
| ${1/\gamma}_{2,2}$ | Average duration spent with CD4 350-500, for those with SPVL 4.0-4.5 (years) | 2.65-3.64 | Netherlands ATHENA cohort ^14^ |
| ${1/\gamma}_{3,2}$ | Average duration spent with CD4 200-350, for those with SPVL 4.0-4.5 (years) | 5.46-15.55 | Netherlands ATHENA cohort ^14^ |
| ${1/\gamma}_{1,3}$ | Average duration spent with CD4>500, for those with SPVL 4.5-5.0 (years) | 2.08-2.64 | Netherlands ATHENA cohort ^14^ |
| ${1/\gamma}_{2,3}$ | Average duration spent with CD4 350-500, for those with SPVL 4.5-5.0 (years) | 1.98-2.72 | Netherlands ATHENA cohort ^14^ |
| ${1/\gamma}_{3,3}$ | Average duration spent with CD4 200-350, for those with SPVL 4.5-5.0 (years) | 4.73-10.22 | Netherlands ATHENA cohort ^14^ |
| ${1/\gamma}_{1,4}$ | Average duration spent with CD4>500, for those with SPVL ≥5.0 (years) | 1.28-1.76 | Netherlands ATHENA cohort ^14^ |
| ${1/\gamma}_{2,4}$ | Average duration spent with CD4 350-500, for those with SPVL ≥5.0 (years) | 1.22-1.69 | Netherlands ATHENA cohort ^14^ |
| ${1/\gamma}_{3,4}$ | Average duration spent with CD4 200-350, for those with SPVL ≥5.0 (years) | 2.12-4.19 | Netherlands ATHENA cohort ^14^ |
| ${1/\sigma}_{0}$ | Average duration from ART initiation to viral suppression (VL < 200 copies/ml) for those with acute HIV infection (months) | 3.93-8.50 | Pregnant women, Kenya^52^ |
| ${1/\sigma}_{1}$ | Average duration from ART initiation to viral suppression (VL < 200 copies/ml) for those with log_10_ SPVL <4.0 (months) | 0.95-4.1 | Data from Johns Hopkins (Baltimore) and Fenway (Boston); estimate is weighted average of median values from 2 sites^19^ |
| ${1/\sigma}_{2}$ | Average duration from ART initiation to viral suppression (VL < 200 copies/ml) for those with log_10_ SPVL 4.0-4.5 (months) | 1.03-4.75 | Data from Johns Hopkins (Baltimore) and Fenway (Boston); estimate is weighted average of median values from 2 sites^19^ |
| ${1/\sigma}_{3}$ | Average duration from ART initiation to viral suppression (VL < 200 copies/ml) for those with log_10_ SPVL 4.5-5.0 (months) | 1.4-6.43 | Data from Johns Hopkins (Baltimore) and Fenway (Boston); estimate is weighted average of median values from 2 sites^19^ |
| ${1/\sigma}_{4}$ | Average duration from ART initiation to viral suppression (VL < 200 copies/ml) for those with log_10_ SPVL >5.0 (months) | 2.03-6.49 | Data from Johns Hopkins (Baltimore) and Fenway (Boston); estimate is weighted average of median values from 2 sites^19^ |
| $f_{1,1}$ | Percentage with CD4 >500 after seroconversion, for those with SPVL <4.0 | 81-91 | Netherlands ATHENA cohort ^14^ |
| $f_{3,1}$ | Percentage with CD4 200-350 after seroconversion, for those with SPVL <4.0 | 0-4 | Netherlands ATHENA cohort ^14^ |
| $f_{4,1}$ | Percentage with CD4 <200 after seroconversion, for those with SPVL <4.0 | 0 (fixed) | Netherlands ATHENA cohort ^14^ |
| $f_{1,2}$ | Percentage with CD4 >500 after seroconversion, for those with SPVL 4.0-4.5 | 72-83 | Netherlands ATHENA cohort ^14^ |
| $f_{3,2}$ | Percentage with CD4 200-350 after seroconversion, for those with SPVL 4.0-4.5 | 1-5 | Netherlands ATHENA cohort ^14^ |
| $f_{4,2}$ | Percentage with CD4 <200 after seroconversion, for those with SPVL 4.0-4.5 | 0 (fixed) | Netherlands ATHENA cohort ^14^ |
| $f_{1,3}$ | Percentage with CD4 >500 after seroconversion, for those with SPVL 4.5-5.0 | 69-79 | Netherlands ATHENA cohort ^14^ |
| $f_{3,3}$ | Percentage with CD4 200-350 after seroconversion, for those with SPVL 4.5-5.0 | 3-8 | Netherlands ATHENA cohort ^14^ |
| $f_{4,3}$ | Percentage with CD4 <200 after seroconversion, for those with SPVL 4.5-5.0 | 0 (fixed) | Netherlands ATHENA cohort ^14^ |
| $f_{1,4}$ | Percentage with CD4 >500 after seroconversion, for those with SPVL ≥5.0 | 64-77 | Netherlands ATHENA cohort ^14^ |
| $f_{3,4}$ | Percentage with CD4 200-350 after seroconversion, for those with SPVL ≥5.0 | 2-7 | Netherlands ATHENA cohort ^14^ |
| $f_{4,4}$ | Percentage with CD4 <200 after seroconversion, for those with SPVL ≥5.0 | 0 (fixed) | Netherlands ATHENA cohort ^14^ |
| ***Transmission probabilities*** | | | |
| $d_{1}$ | Relative infectiousness of HIV-positive partner in acute stage of infection vs chronic & CD4>200 (off ART) | 4.47-18.81 | Systematic review of studies of heterosexual transmission^12^ |
| $d_{2}$ | Relative infectiousness of HIV-positive partner in late stage of infection – CD4<200 cells per µL vs chronic and CD4>200 (off ART) | 2-8 | Cohort study among African heterosexual adults^53,54^ |
| $\beta$ | Average probability of acquiring HIV infection per sex act with an HIV-positive partner with chronic untreated infection | 0.0007-0.0285 | Systematic review of anal transmission and study among Australian MSM^21,22^; assume 50% of sex acts are insertive |
| $h_{1}$ | Relative infectiousness of HIV-positive person with log_10_ SPVL <4.0 vs 4.0-4.5 | 0.337-0.68 | ^23^ Inverse of pooled increase in transmissibility per log10 decrease in viral load |
| $h_{2}$ | Relative infectiousness of HIV-positive person with log_10_ SPVL 4.0-4.5 vs 4.0-4.5 | 1 (fixed) | _ |
| $h_{3}$ | Relative infectiousness of HIV-positive person with log_10_ SPVL 4.5-5.0 vs 4.0-4.5 | 1 (fixed) | _ |
| $h_{4}$ | Relative infectiousness of HIV-positive person with log_10_ SPVL >5.0 vs 4.0-4.5 | 1.47-2.97 | ^23^ pooled increase in transmissibility per log10 increase in viral load |
| $r_{0}$ | Relative susceptibility of Black MSM | 1 (fixed) |  |
| $r_{1}$ | Relative susceptibility of White MSM | 0.2-1 | Varied to fit race-specific HIV prevalence; accounts for remaining differences in risk by race not captured by other parameters |
| ***Intervention behaviour*** | | | |
| $p_{0}$ | Percentage of new Black entrants to MSM population who never routinely test for HIV | 1-11 | NHBS Atlanta MSM 2011-2014 (highest and lowest from 95% CI across both years): % of those aged >24 years old who report never testing for HIV |
| $p_{ratio}$ | Ratio of percentage of new entrants who never routinely test for HIV for White:Black MSM | 0.13-0.63 | NHBS Atlanta MSM ratio in 2014 (lower bound), ratio in 2011 (upper bound) for % of those aged >24 years old who report never testing for HIV White:Black |
| ${test}_{0,0}$ | Percentage of undiagnosed Black MSM aged 18-24 testing for HIV in the last year, 2004 onwards^b^ | 29-48 (diagnosis fitting) | NHBS data 2011-2014(highest and lowest from 95% CI), self-reported HIV negative men, reduced by 50-60% for fitting to diagnosis data; converted into rate of testing at least once per year in the model ($\tau_{0.0.1}$) |
| $test_{0,1}$ | Percentage of undiagnosed White MSM aged 18-24 testing for HIV in the last year, 2004 onwards^b^ | 13-46 (diagnosis fitting) | NHBS data 2011-2014(highest and lowest from 95% CI), self-reported HIV negative men, reduced by 50-60% for fitting to diagnosis data; converted into rate of testing at least once per year in the model ($\tau_{0.1.1}$) |
| ${test}_{1,0}$ | Percentage of undiagnosed Black MSM aged >24 years old testing for HIV in the last year, 2004 onwards^b^ | 25-42 (diagnosis fitting) | NHBS data 2011-2014(highest and lowest from 95% CI), self-reported HIV negative men, reduced by 50-60% for fitting to diagnosis data; converted into rate of testing at least once per year in the model ($\tau_{1.0.1}$) |
| ${test}_{1,1}$ | Percentage of undiagnosed White MSM aged >24 years old testing for HIV in the last year, 2004 onwards^b^ | 25-42 (diagnosis fitting) | NHBS data 2011-2014(highest and lowest from 95% CI), self-reported HIV negative men, reduced by 50-60% for fitting to diagnosis data; converted into rate of testing at least once per year in the model ($\tau_{1.1.1}$) |
| ${test}_{early}$ | Percentage of all MSM who tested for HIV in the last year, 1996 | 8-15 (diagnosis fitting) | MSM in national NHSDA survey 1996 ^30^, reduced by 50-60% for fitting to diagnosis data; converted into rate of testing at least once per year in the model |
| $\tau_{0.0.2}$,$\tau_{0.1.2}$,$\tau_{1.0.2}$,$\tau_{1.1.2}$ | Number of HIV tests per year among those on PrEP | 4 (fixed) | Based on recommended frequency of HIV testing for those on PrEP at least 4 times per year^3^ |
| *q*_1,1_ | Percentage of White MSM not on PrEP testing positive for HIV who link to care straight away, 2008 onwards^b^ | 70-86 | Lower bound: Georgia DPH report 2012^31^ – calculated from % all and % White linking to care within 3 months of diagnosis  Upper bound: Georgia DPH report 2014^32^: % White MSM linking within 1 month of diagnosis |
| *q*_2,0_, *q*_2,1_ | Percentage of Black, White MSM on PrEP testing positive for HIV who link to care straight away, 2012 onwards | 100 | Assumption |
| $\epsilon_{1}$ | Rate of linkage to care for White MSM not linking immediately or dropped out, per year |  | Estimate |
| linkage_inc | Annual absolute increase in percentage of White MSM who link to care straight away after testing positive for HIV, 1979-2008 | 3.5 (fixed) | From changes for MSM in national CDC data ^33,34^ |
| $\epsilon_{ratio}$ | Ratio of rates of linkage to care for Black:White MSM (ratio also applied to percentage linking immediately after diagnosis for those not on PrEP) | 0.76-1.02 | Georgia DPH data 2011, 2014^32,55^, National estimates for MSM from NHBS and NHSS data ^43,56^ |
| $\omega_{1}$ | Ratio of rate of dropout from care: rate of dropout from ART for White MSM | 1-7 | Estimates from US studies - risk of dropout from care for those on vs off ART ^35-37^ |
| $\omega_{ratio}$ | Ratio of dropout from care for White:Black MSM | 0.46-1.54 | Studies of US cohort data and US HIV surveillance data ^35-40^ |
| $\xi_{x,1}$ | Rate of initiation onto ART from care for White MSM, when meeting CD4 criteria^c^, per year^b^ | 1.1-2 | Assuming CD4 testing less frequent than the recommendation in national guidelines (every 3-6 months), with acceptance of 80-90% ^57^ |
| $\xi_{ratio}$ | Ratio of ART initiation rate for Black:White MSM | 0.4-1.0 | US ART cohort data^37^ |
| ${\psi_{0,z}, \psi}_{1,z}$ | Rate of starting HAART due to AIDS symptoms, CD4>500, per year (post-1996) | 0.002-0.01 | Incidence of AIDS-defining illness among ART naives, CASCADE collaboration ^42^; similar estimates from EURO-COORD data analysis ^41^ |
| $\psi_{2,z}$ | Rate of starting HAART due to AIDS symptoms, CD4 350-500, per year (post-1996) | 0.008-0.015 | Incidence of AIDS-defining illness among ART naives, CASCADE collaboration ^42^; similar estimates from EURO-COORD data analysis ^41^ |
| $\psi_{3,z}$ | Rate of starting HAART due to AIDS symptoms, CD4 200-350, per year (post-1996) | 0.018-0.032 | Incidence of AIDS-defining illness among ART naives, CASCADE collaboration ^42^; similar estimates from EURO-COORD data analysis ^41^ |
| $\psi_{4,z}$ | Rate of starting HAART due to AIDS symptoms, CD4<200, per year (post-1996) | 0.173-0.262 | Incidence of AIDS-defining illness among ART naives, CASCADE collaboration ^42^ |
| $\chi_{1}$ | Percentage of White MSM initiating ART who are adherent (achieve viral suppression) | 73-99 | Studies of US cohort data and US HIV surveillance data ^43-46^ |
| $\chi_{ratio}$ | Ratio of percentage adherent to ART Black:White MSM | 0.82-1 | US cohort data and US HIV surveillance^35,43-47^ |
| $\phi_{5,1}\phi_{6,1}\phi_{7,1}$ | Dropout from ART, White MSM, not fully suppressed/1^st^ year on ART/2^nd^ year on ART, per year | 0.06-0.13 | Rate of dropout from ART, US ^35-39^ |
| $\phi_{z ratio}$ | Ratio of dropout from ART 3^rd^+ years: dropout 1^st^, 2^nd^ years ($\phi_{8}: \phi_{5}$) | 0.5-1.0 | Rate of dropout from US ART cohorts ^48^ |
| $\phi_{w ratio}$ | Ratio of ART dropout for Black:White MSM | 0.7-1.6 | US ART cohort data^38,48^ |
| $\zeta$ | Rate of re-enrolment into pre-ART HIV care for those dropping out of ART, per year | 0.05-1 | From rate of dropout and re-joining US ART cohorts ^48^ |
| $s_{n,0}$ | Percentage of Black MSM circumcised | 75-87 | NHBS 2014 |
| $s_{n,1}$ | Percentage of White MSM circumcised | 87-96 | NHBS 2014 |
| $\delta_{1}$ | Relative rate of being offered PrEP: testing for HIV, for those with a PrEP indication, 2020 onwards^b^ | 2-6 | Range explored; rate increases linearly from 0 in 2012 |
| $\delta_{0}$ | Relative rate of being offered PrEP: testing for HIV, for those without a PrEP indication, 2020 onwards | 0 | In main analysis, assume no PrEP initiations by those without a PrEP indication |
| $o_{0,0}$ | PrEP acceptance (% accepting PrEP when offered), 18-24 year-old Black MSM | 40.8-64.2 (mode 52.6)^d^ | Stratified analysis of US PrEP Demo project data |
| $o_{0,1}$ | PrEP acceptance (% accepting PrEP when offered), 18-24 year-old White MSM | 64.6-74.0 (mode 69.5)^d^ | Stratified analysis of US PrEP Demo project data |
| $o_{1,0}$ | PrEP acceptance (% accepting PrEP when offered), >24 year-old Black MSM | 40.8-64.2 (mode 52.6)^d^ | Stratified analysis of US PrEP Demo project data |
| $o_{1,1}$ | PrEP acceptance (% accepting PrEP when offered), >24 year-old White MSM | 64.6-74.0 (mode 69.5) ^d^ | Stratified analysis of US PrEP Demo project data |
| $s_{ph0,0}$ | Adherence to daily oral PrEP with TDF/FTC (% taking ≥4 doses/week), 18-24 year old Black MSM [values for sensitivity analysis in square brackets] | 47.2-82.7 (mode 67.3) ^d^  [58-64 (mode 61)] | Stratified analysis of US PrEP Demo project data [for sensitivity analysis: race-specific values for randomly selected adherence subset of MSM in the HPTN 083 trial^49^] |
| $s_{ph0,1}$ | Adherence to daily oral PrEP with TDF/FTC (% taking ≥4 doses/week), 18-24 year old White MSM [values for sensitivity analysis in square brackets] | 85.8-97.5 (mode 93.2) ^d^  [75-81 (mode 78)] | Stratified analysis of US PrEP Demo project data [for sensitivity analysis: race-specific values for randomly selected adherence subset of MSM in the HPTN 083 tria^49^] |
| $s_{ph1,0}$ | Adherence to daily oral PrEP with TDF/FTC (% taking ≥4 doses/week), >24 year-old Black MSM [values for sensitivity analysis in square brackets] | 43.7-64.2 (mode 54.1) ^d^  [58-64 (mode 61)] | Stratified analysis of US PrEP Demo project data [for sensitivity analysis: race-specific values randomly selected adherence subset of MSM in the HPTN 083 tria^49^] |
| $s_{ph1,1}$ | Adherence to daily oral PrEP with TDF/FTC (% taking ≥4 doses/week), >24 year-old White MSM [values for sensitivity analysis in square brackets] | 87.7-92.7 (mode 90.4) ^d^  [75-81 (mode 78)] | Stratified analysis of US PrEP Demo project data [for sensitivity analysis: race-specific values for randomly selected adherence subset of MSM in the HPTN 083 tria^49^] |
| $s_{pm0,0}$, $s_{pm0,1}$,$s_{pm1,0}$,$s_{pm1,1}$ | % of those taking <4 doses/week who take 2-4 doses/week of daily oral TDF/FTC | 45 (fixed) | Percentage estimated from full followup (of up to 81 weeks) of randomly selected adherence subset of MSM in the HPTN 083 trial^58^ |
| $\iota_{0,0}$ | PrEP dropout rate per person per year, 18-24 year old Black MSM | 0.280-0.849  (mode 0.511) ^d^ | Rate calculated from proportion not retained at the end of the study, stratified analysis of US PrEP Demo project data |
| $\iota_{0,1}$ | PrEP dropout rate per person per year, 18-24 year old White MSM | 0.033-0.357  (mode 0.190) ^d^ | Rate calculated from proportion not retained at the end of the study, stratified analysis of US PrEP Demo project data |
| $\iota_{1,0}$ | PrEP dropout rate per person per year, >24 year-old Black MSM | 0.280-0.849  (mode 0.511) ^d^ | Rate calculated from proportion not retained at the end of the study, stratified analysis of US PrEP Demo project data |
| $\iota_{1,1}$ | PrEP dropout rate per person per year, >24 year-old White MSM | 0.151-0.270  (mode 0.216) ^d^ | Rate calculated from proportion not retained at the end of the study, stratified analysis of US PrEP Demo project data |
| ***Intervention efficacy*** | | | |
| $e_{c}$ | Reduction in HIV acquisition risk due to correct condom use (%) | 58-79 | Estimate for US MSM^24^ |
| $e_{n}$ | Reduction in HIV acquisition risk due to male circumcision (%) | 12-23 | Assuming same efficacy as for heterosexual men from RCTs^25^, only protective in insertive acts, half of all sex acts are insertive, receptive sex acts carry a 2.3x higher risk of transmission than insertive^22^ |
| $d_{r}$ | Relative level of infectiousness of those on ART and partially suppressed, scaled between the level for those fully suppressed ${(d}_{r}=0$) and those unsuppressed ($d_{r}=1)$ | 0.5 (fixed) | assumption |
| $d_{6}$ | Relative level of infectiousness of those on ART and fully suppressed vs chronic infection untreated (CD4>200) | 0 (fixed) | Equivalent to 100% reduction in HIV transmission. Estimates from discordant MSM partnerships where HIV-positive partner on ART and virally suppressed^26^ |
| $e_{ph}$ | Reduction in HIV acquisition risk when adherent to daily oral PrEP with TDF/FTC (taking ≥4 tablets/week) | 90-100 (mode 96) ^d^ | Efficacy estimated by Anderson et al from iPrEx and STRAND trial data analysis for 4 doses/week^27^ |
| $e_{pm}$ | Reduction in HIV acquisition risk when partially adherent to daily oral PrEP with TDF/FTC (taking 2-4 tablets/week) | 56-96 (mode 76) ^d^ | Efficacy estimated by Anderson et al from iPrEx and STRAND trial data analysis for 2 doses/week^27^ |
| $e_{pi}$ | Reduction in HIV acquisition risk when using long-acting injectable CAB (including those not adhering to injection schedule) | 82-96 (mode 91)^d^ | Effectiveness (efficacy × adherence) estimated from HPTN 083 trial data |

^a^Limits of uniform/triangular prior distribution

^b^Final values for time-varying parameters. Earlier values or earlier gradient of parameter function given elsewhere in table S1.

^c^Guideline changes coded in: pre-1996, no initiation of ART ^59^ From 1996-1998 ART initiation at any CD4 count; from 1998-Feb 2001, initiation from care with CD4<500 (1998 guidelines); from Feb 2001-Dec 2009 initiation with CD4 <350 (2001 guidelines); from Dec 2009-March 2012 initiation from care with CD4<500 (2009 guidelines); from March 2012 onwards initiation from care with any CD4 count (2012 guidelines). These apply to all age and race groups.

^d^Triangular distribution used (mode given in brackets)

## Table of fitting data

**Table S2: Data fitted to, with fitting bounds, source and justification**

| **Output** | **Year** | **Estimate** | **Min** | **Max** | **Source & justification** | **Used for fitting** | **Used for validation** |
| --- | --- | --- | --- | --- | --- | --- | --- |
| **Demography** |  |  |  |  |  |  |  |
| Total MSM population size | 2010 | 6518 | 52098 | 71860 | Range 2.9-4%^1,2^ of male population aged 18+ in Atlanta 2010 census (1,796,488) | ✓ |  |
| Percentage of population aged 18-24 | 2004 | 18.0 | 10.3 | 25.8 | NHBS data 95% CI | ✓ |  |
|  | 2011 | 21.3 | 15.4 | 28.7 | NHBS data 95% CI | ✓ |  |
|  | 2014 | 19.5 | 12.8 | 28.6 | NHBS data 95% CI | ✓ |  |
| Percentage of White MSM aged 18-24 | 2004 | 9.8 | 6.2 | 13.4 | NHBS data 95% CI | ✓ |  |
|  | 2011 | 10.8 | 7.3 | 15.7 | NHBS data 95% CI | ✓ |  |
|  | 2014 | 9.7 | 5.4 | 16.7 | NHBS data 95% CI | ✓ |  |
| Percentage of Black MSM aged 18-24 | 2004 | 30.2 | 14.4 | 46.0 | NHBS data 95% CI | ✓ |  |
|  | 2011 | 30.2 | 19.6 | 43.4 | NHBS data 95% CI | ✓ |  |
|  | 2014 | 23.6 | 13.6 | 37.9 | NHBS data 95% CI | ✓ |  |
| Percentage of MSM who are Black | 2004 | 36.6 | 24.0 | 49.2 | NHBS data 95% CI | ✓ |  |
|  | 2011 | 45 | 34.5 | 56.1 | NHBS data 95% CI | ✓ |  |
|  | 2014 | 62 | 40.0 | 73.8 | NHBS data 95% CI; lower bound reduced from 48.6% to enable fitting to race data | ✓ |  |
| Percentage of Black 18-24 year old MSM with a PrEP indication | 2014 | 62.5 | 46.3 | 76.3 | NHBS data 95% CI | ✓ |  |
| Percentage of Black >24 year old MSM with a PrEP indication | 2014 | 53.0 | 45.0 | 60.9 | NHBS data 95% CI | ✓ |  |
| Percentage of White 18-24 year old MSM with a PrEP indication | 2014 | 35.7 | 13.4 | 66.6 | NHBS data 95% CI | ✓ |  |
| Percentage of White >24 year old MSM with a PrEP indication | 2014 | 54.2 | 47.4 | 60.8 | NHBS data 95% CI | ✓ |  |
| **HIV prevalence** |  |  |  |  |  |  |  |
| HIV prevalence Black MSM aged 18-24 years old | 2011 | 23.6 | 12.6 | 39.9 | NHBS data 95% CI | ✓ |  |
|  | 2014 | 28.6 | 18.8 | 40.8 | NHBS data 95% CI | ✓ |  |
| HIV prevalence Black MSM aged >24 years old | 2011 | 40.2 | 31.8 | 49.1 | NHBS data 95% CI | ✓ |  |
|  | 2014 | 49.7 | 42.1 | 57.3 | NHBS data 95% CI | ✓ |  |
| HIV prevalence White MSM aged 18-24 years old | 2011 | 0 | 0 | - | NHBS data 95% CI (numbers too small) |  |  |
|  | 2014 | 7.1 | 0.9 | 38.6 | NHBS data 95% CI | ✓ |  |
| HIV prevalence White MSM aged >24 years old | 2011 | 21.7 | 16.7 | 27.7 | NHBS data 95% CI | ✓ |  |
|  | 2014 | 23.7 | 15.7 | 34.1 | NHBS data 95% CI | ✓ |  |
| HIV prevalence Black MSM | 2017 | 38 | 32.5 | 43.5 | NHBS data, reported by Georgia DPH^60^ |  | ✓ |
| HIV prevalence White MSM | 2017 | 15 | 7.5 | 22.5 | NHBS data, reported by Georgia DPH^60^ |  | ✓ |
| **Care continuum indicators** |  |  |  |  |  |  |  |
| Percentage of HIV-positive MSM diagnosed | 2012 | 79.2 | 76.3 | 82.9 | CDC data for Georgia state^61^95% CI | ✓ |  |
| Percentage of diagnosed MSM in care | 2011 | 57.1 | 47.1 | 67.1 | Georgia DPH data^a^ ±10pp^55^ MSM data | ✓ |  |
|  | 2012 | 56 | 46 | 66 | Georgia DPH data^a^ ±10pp^31^ MSM data | ✓ |  |
|  | 2014 | 62 | 52 | 72 | Georgia DPH data^a^ ±10pp^32^ MSM data | ✓ |  |
|  | 2016 | 66 | 56 | 76 | Georgia DPH data^a^ ±10pp^62^ MSM data | ✓ |  |
|  | 2017 | 68 | 58 | 78 | Georgia DPH data^a^ ±10pp^63^ MSM data | ✓ |  |
|  | 2018 | 71 | 61 | 81 | Georgia DPH data^a^ ±10pp^64^ MSM data | ✓ |  |
|  | 2019 | 73 | 63 | 83 | Georgia DPH data^a^ ±10pp^65^ MSM data | ✓ |  |
| Percentage of diagnosed Black MSM in care | 2011 | 56.7 | 46.7 | 66.7 | Georgia DPH data^a^ ±10pp^55^ MSM data | ✓ |  |
|  | 2012 | 54.6 | 45.6 | 65.6 | Georgia DPH data^a^ ±10pp^31^ MSM data | ✓ |  |
|  | 2014 | 61.0 | 51.0 | 71.0 | Georgia DPH data^a^ ±10pp^32^ MSM data | ✓ |  |
|  | 2016 | 65 | 55 | 75 | Georgia DPH data^a^ ±10pp^62^ data for all PLHIV |  | ✓ |
|  | 2017 | 65 | 55 | 75 | Georgia DPH data^a^ ±10pp^63^ data for all PLHIV |  | ✓ |
|  | 2018 | 70 | 60 | 80 | Georgia DPH data^a^ ±10pp^64^ data for all PLHIV |  | ✓ |
|  | 2019 | 71 | 61 | 81 | Georgia DPH data^a^ ±10pp^65^ data for all PLHIV |  | ✓ |
| Percentage of diagnosed White MSM in care | 2011 | 55.6 | 45.6 | 65.6 | Georgia DPH data^a^ ±10pp^55^ MSM data | ✓ |  |
|  | 2012 | 55.6 | 45.6 | 65.6 | Georgia DPH data^a^ ±10pp^31^ MSM data | ✓ |  |
|  | 2014 | 64.0 | 54.0 | 74.0 | Georgia DPH data^a^ ±10pp^32^ MSM data | ✓ |  |
|  | 2016 | 64 | 54 | 74 | Georgia DPH data^a^ ±10pp^62^ data for all PLHIV |  | ✓ |
|  | 2017 | 66 | 56 | 76 | Georgia DPH data^a^ ±10pp^63^ data for all PLHIV |  | ✓ |
|  | 2018 | 69 | 59 | 79 | Georgia DPH data^a^ ±10pp^64^ data for all PLHIV |  | ✓ |
|  | 2019 | 73 | 63 | 83 | Georgia DPH data^a^ ±10pp^65^ data for all PLHIV |  | ✓ |
| Percentage of diagnosed MSM virally suppressed | 2011 | 41.54 | 31.54 | 51.54 | Georgia DPH data^a^ ±10pp^55^ MSM data | ✓ |  |
|  | 2012 | 40.0 | 30.0 | 50.0 | Georgia DPH data^a^ ±10pp^31^ MSM data | ✓ |  |
|  | 2014 | 47.0 | 37.0 | 57.0 | Georgia DPH data^a^ ±10pp^32^ MSM data | ✓ |  |
|  | 2016 | 51.0 | 41.0 | 61.0 | Georgia DPH data^a^ ±10pp^62^ MSM data | ✓ |  |
|  | 2017 | 52.0 | 42.0 | 62.0 | Georgia DPH data^a^ ±10pp^63^ MSM data | ✓ |  |
|  | 2018 | 55.0 | 45.0 | 65.0 | Georgia DPH data^a^ ±10pp^64^ MSM data | ✓ |  |
|  | 2019 | 61.0 | 51.0 | 71.0 | Georgia DPH data^a^ ±10pp^64^ MSM data | ✓ |  |
| Percentage of diagnosed Black MSM virally suppressed | 2011 | 37.6 | 27.6 | 47.6 | Georgia DPH data^a^ ±10pp^55^ MSM data | ✓ |  |
|  | 2012 | 35.9 | 25.9 | 45.9 | Georgia DPH data^a^ ±10pp^31^ MSM data | ✓ |  |
|  | 2014 | 43.0 | 33.0 | 53.0 | Georgia DPH data^a^ ±10pp^32^ MSM data | ✓ |  |
|  | 2016 | 50.0 | 40.0 | 60.0 | Georgia DPH data^a^ ±10pp^62^ data for all PLHIV |  | ✓ |
|  | 2017 | 50.0 | 40.0 | 60.0 | Georgia DPH data^a^ ±10pp^63^ data for all PLHIV |  | ✓ |
|  | 2018 | 54.0 | 44.0 | 64.0 | Georgia DPH data^a^ ±10pp^64^ data for all PLHIV |  | ✓ |
|  | 2019 | 58.0 | 48.0 | 68.0 | Georgia DPH data^a^ ±10pp^65^ data for all PLHIV |  | ✓ |
| Percentage of diagnosed White MSM virally suppressed | 2011 | 46.38 | 36.38 | 56.38 | Georgia DPH data^a^ ±10pp^55^ MSM data | ✓ |  |
|  | 2012 | 45.8 | 35.8 | 55.8 | Georgia DPH data^a^ ±10pp^31^ MSM data | ✓ |  |
|  | 2014 | 54.0 | 44.0 | 64.0 | Georgia DPH data^a^ ±10pp^32^ MSM data | ✓ |  |
|  | 2016 | 56.0 | 46.0 | 66.0 | Georgia DPH data^a^ ±10pp^62^ data for all PLHIV |  | ✓ |
|  | 2017 | 56.0 | 46.0 | 66.0 | Georgia DPH data^a^ ±10pp^63^ data for all PLHIV |  | ✓ |
|  | 2018 | 60.0 | 50.0 | 70.0 | Georgia DPH data^a^ ±10pp^64^ data for all PLHIV |  | ✓ |
|  | 2019 | 66.0 | 56.0 | 76.0 | Georgia DPH data^a^ ±10pp^65^ data for all PLHIV |  | ✓ |
| Percentage of MSM on ART virally suppressed | 2010 | 85 | 75 | 90 | National estimates for MSM^43,66^ range | ✓ |  |
| **PrEP coverage** |  |  |  |  |  |  |  |
| Percentage of all MSM taking PrEP | 2014 | 3.5 | 1.8 | 6.8 | NHBS data on use past 12 months 95% CI, multiplied by 0.8 to estimate current use^67^ | ✓ |  |
|  | 2015 | 11.9 | 5.9 | 20.8 | Study among Grindr-using MSM in Atlanta^68^ | ✓ |  |
|  | 2017 | 17.3 | 13.4 | 21.9 | NHBS data on use past 12 months^69^, multiplied by 0.8 to estimate current use^67^ | ✓ |  |
|  | 2018 | 20.6 | 16.0 | 25.8 | Study among MSM and TGW attending Gay pride events in Atlanta^70^ | ✓ |  |
| Percentage of Black MSM taking PrEP | 2013 | 1.1 | 0.2 | 3.3 | Study among MSM in Atlanta^71^ | ✓ |  |
|  | 2014 | 4.8 | 2.8 | 7.7 | Study among MSM and TGW attending Gay pride events in Atlanta^72^ | ✓ |  |
|  | 2018 | 16.7 | 11.1 | 23.6 | Study among MSM and TGW attending Gay pride events in Atlanta^70^ | ✓ |  |
| Percentage of White MSM taking PrEP | 2018 | 25.2 | 17.9 | 33.7 | Study among MSM and TGW attending Gay pride events in Atlanta^70^; estimate for non-Black MSM |  | ✓ |
| Percentage of 18-24 year old Black MSM taking PrEP | 2014 | 2.0 | 0.3 | 13.0 | NHBS data on use past 12 months 95% CI, multiplied by 0.8 to estimate current use^67^ | ✓ |  |
|  | 2016 | 4.2 | 1.8 | 8.0 | Baseline use in cohort study among MSM in Atlanta^73^ | ✓ |  |
| Percentage of >24 year old Black MSM taking PrEP | 2014 | 0.9 | 0.1 | 5.8 | NHBS data on use past 12 months 95% CI, multiplied by 0.8 to estimate current use^67^ | ✓ |  |
| Percentage of >24 year old White MSM taking PrEP | 2014 | 8.8 | 3.9 | 18.3 | NHBS data on use past 12 months 95% CI, multiplied by 0.8 to estimate current use^67^ | ✓ |  |

^a^definition of in care: percentage of those diagnosed with at least one CD4 test or viral load test past 12 months

^b^definition of virally suppressed: persons with viral suppression among all diagnosed

# SUPPLEMENTAL RESULTS

**Supplementary Figures**


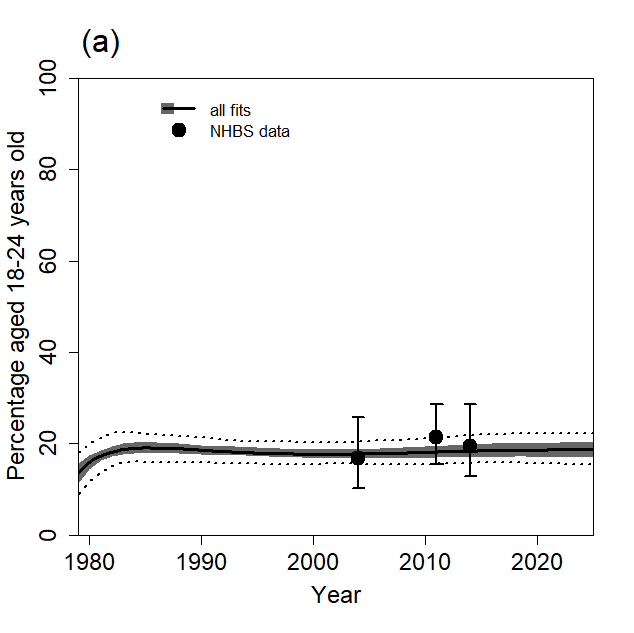

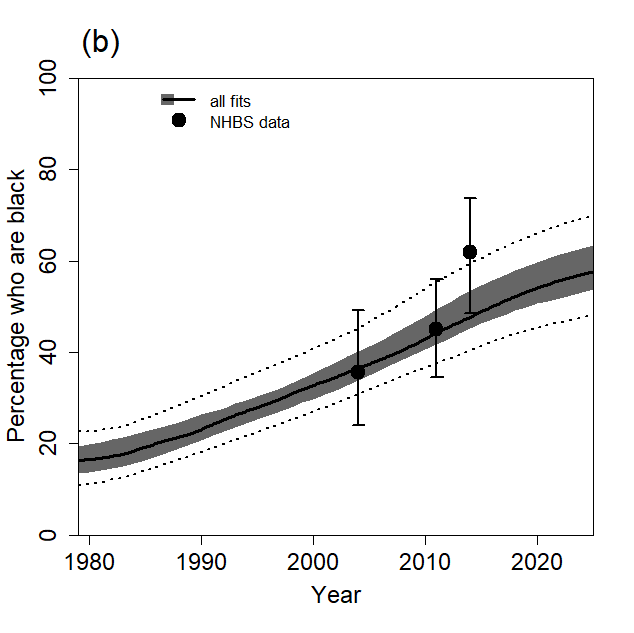

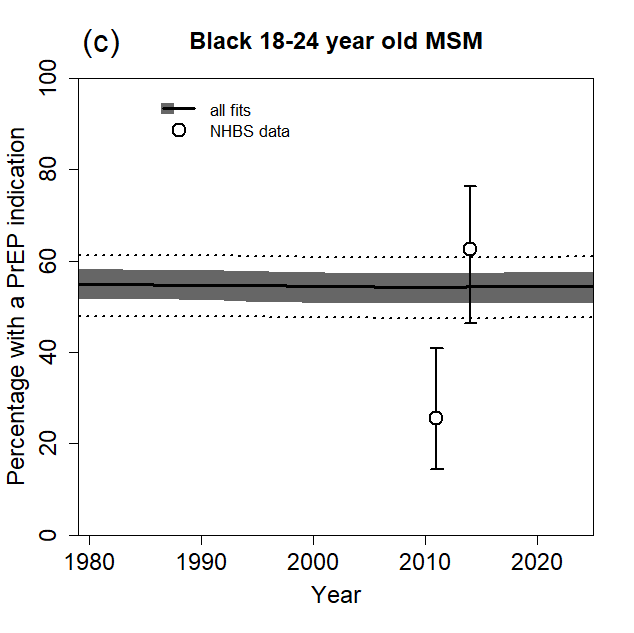

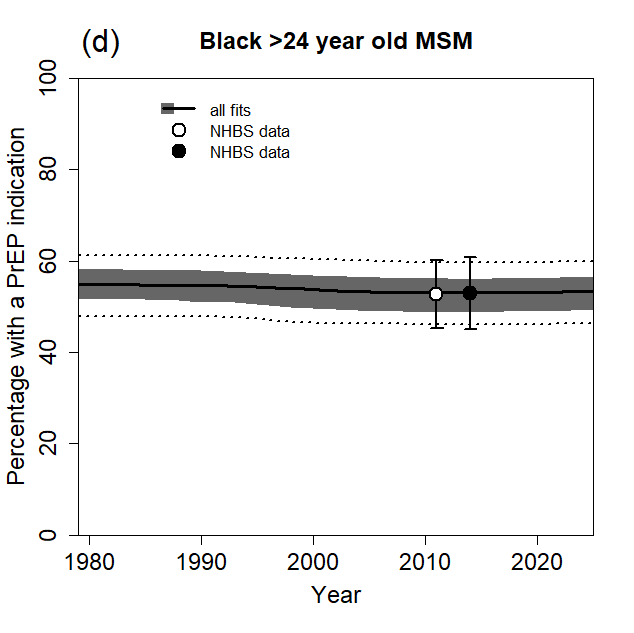

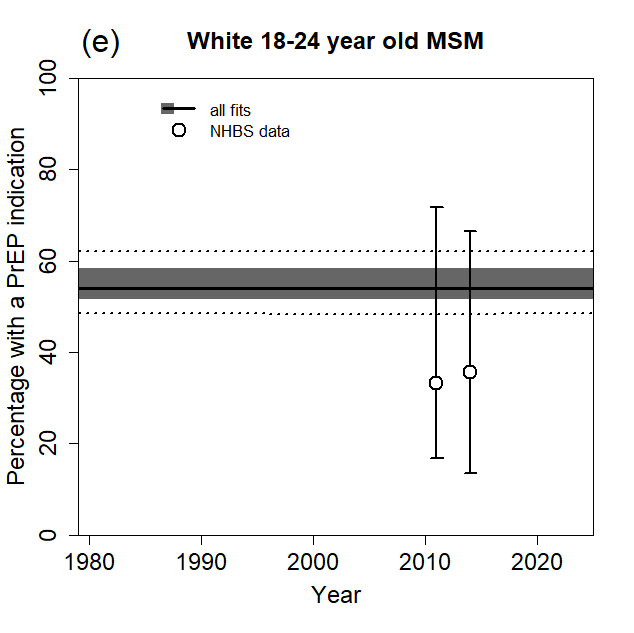

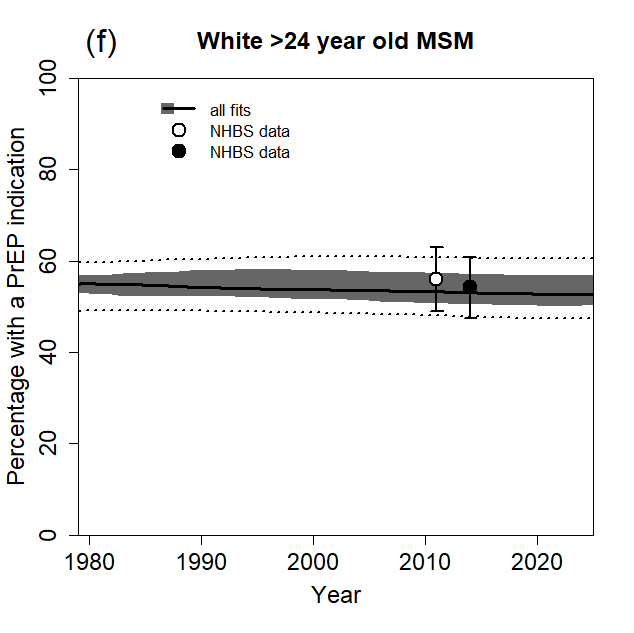

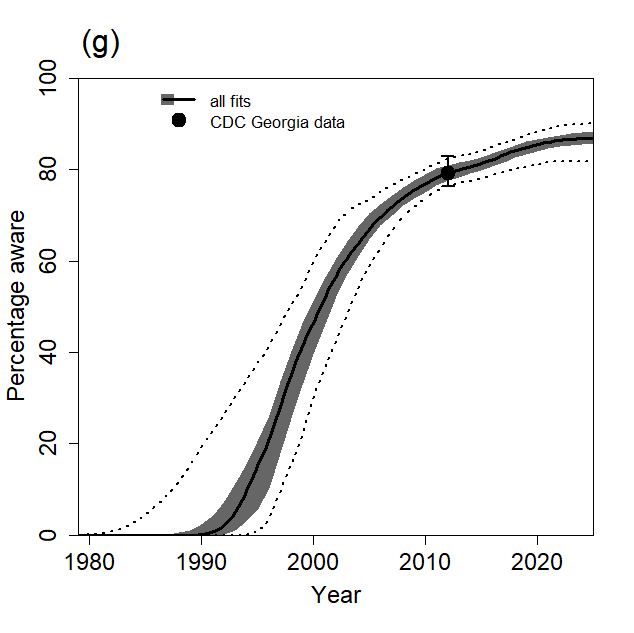

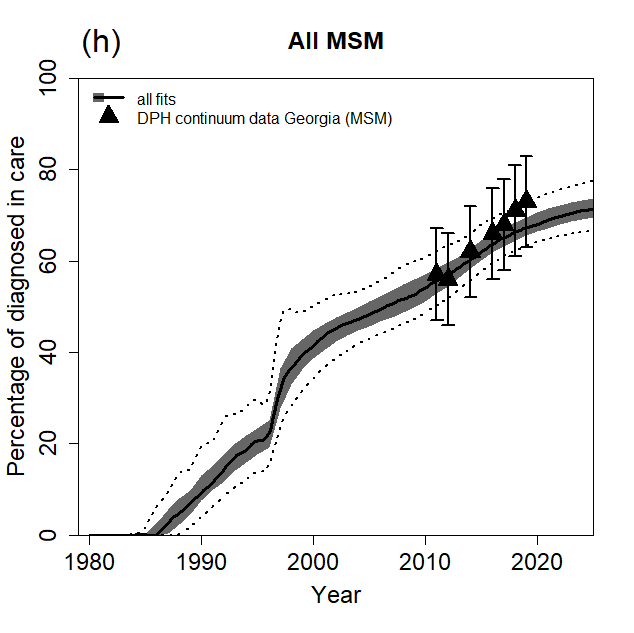

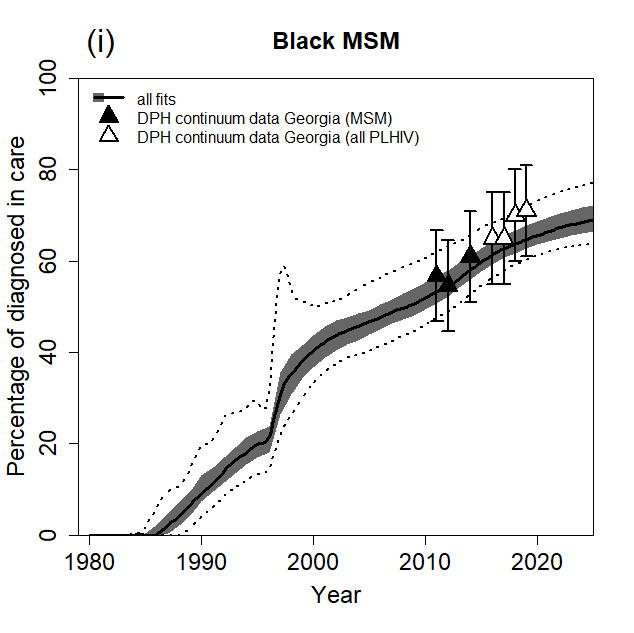

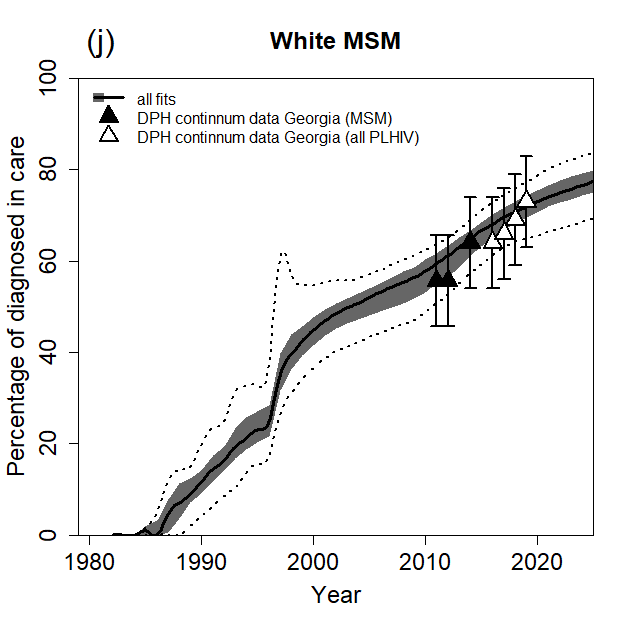

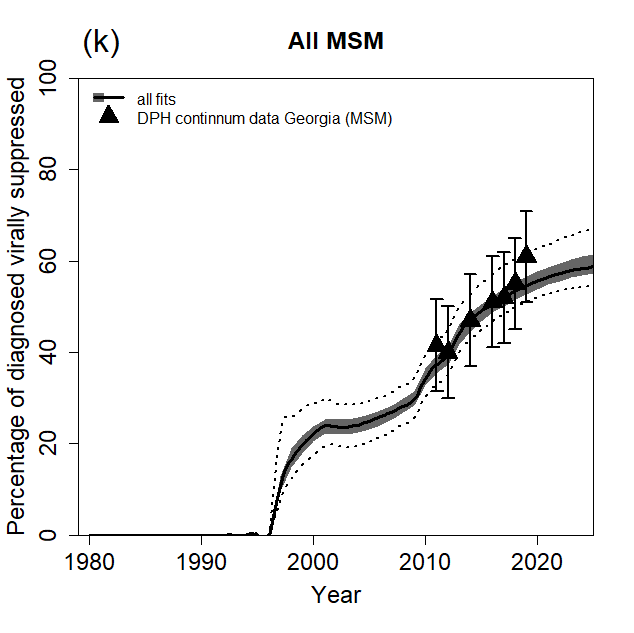

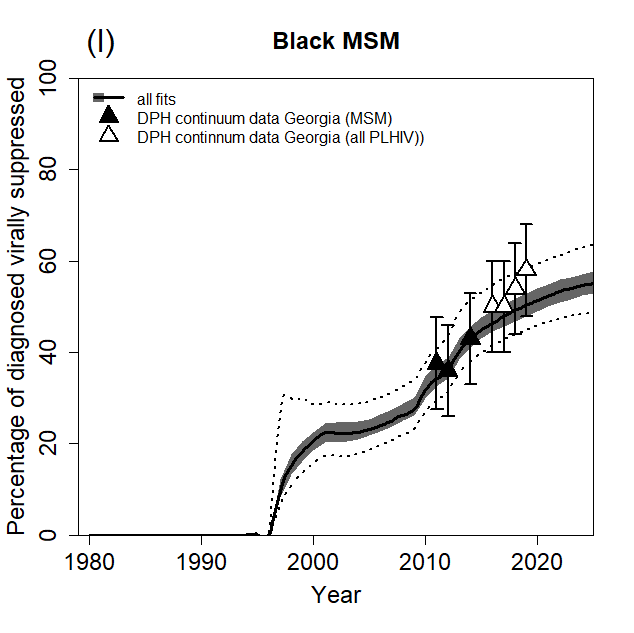


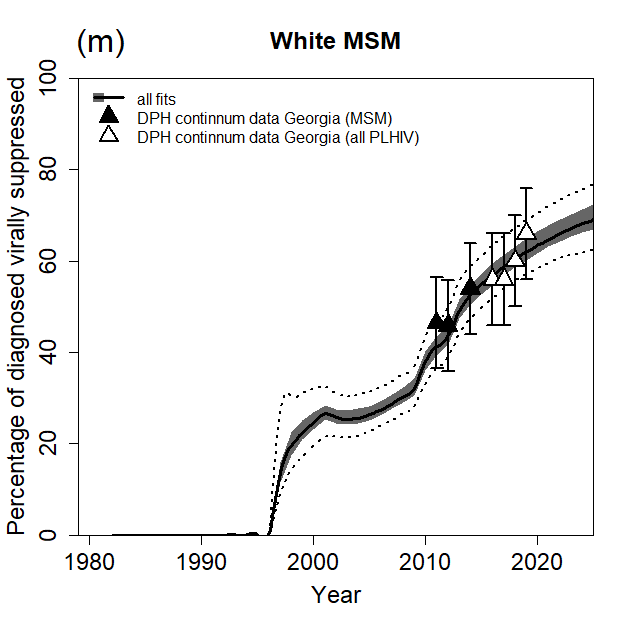

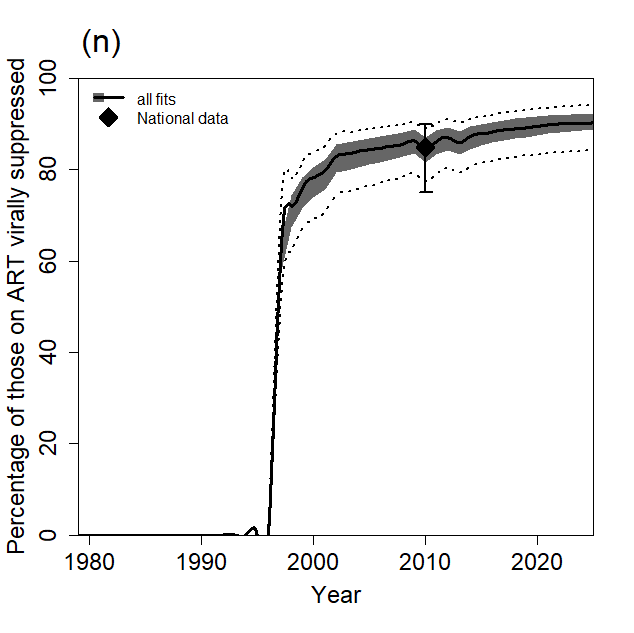


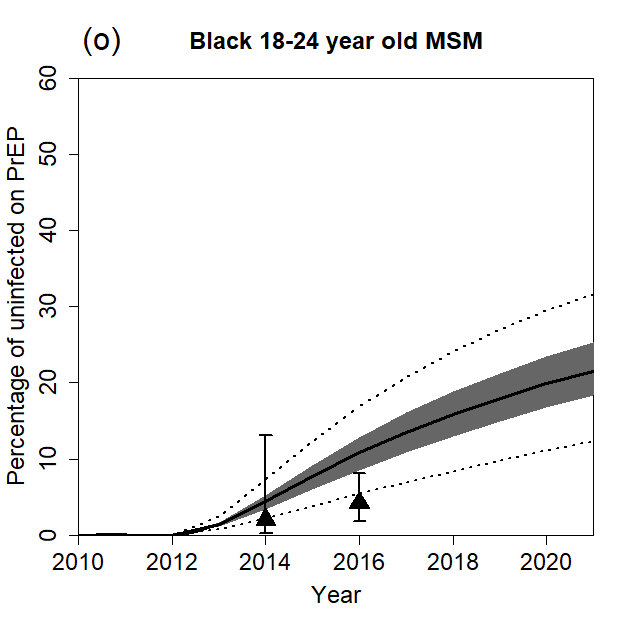

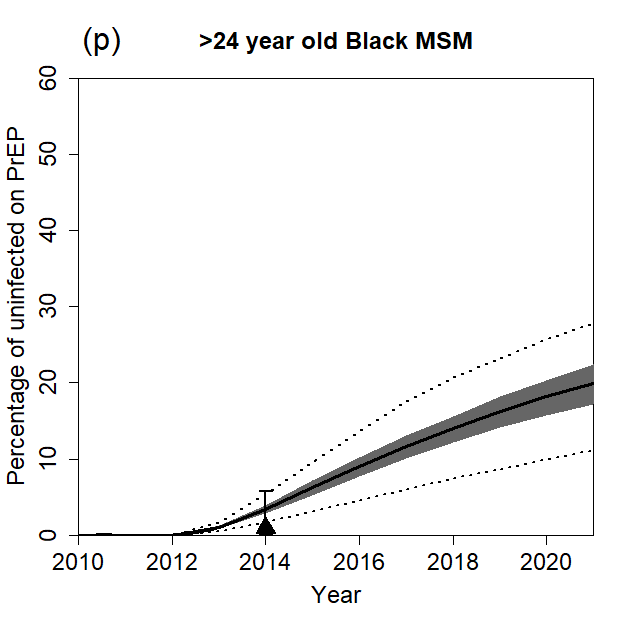

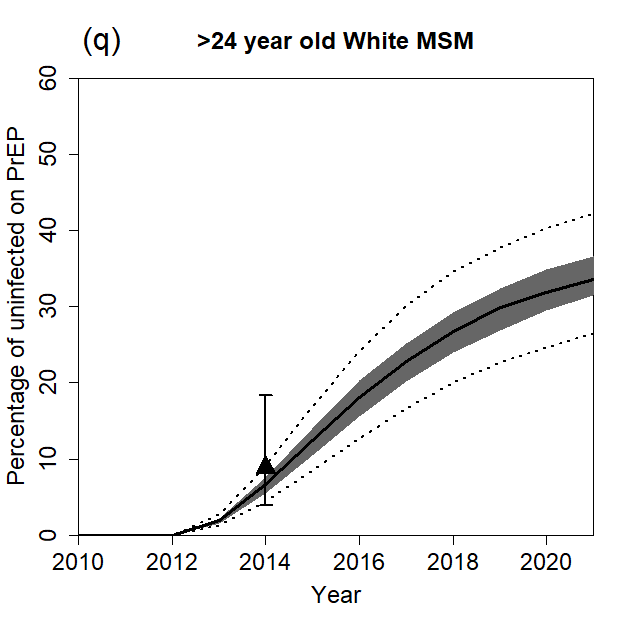

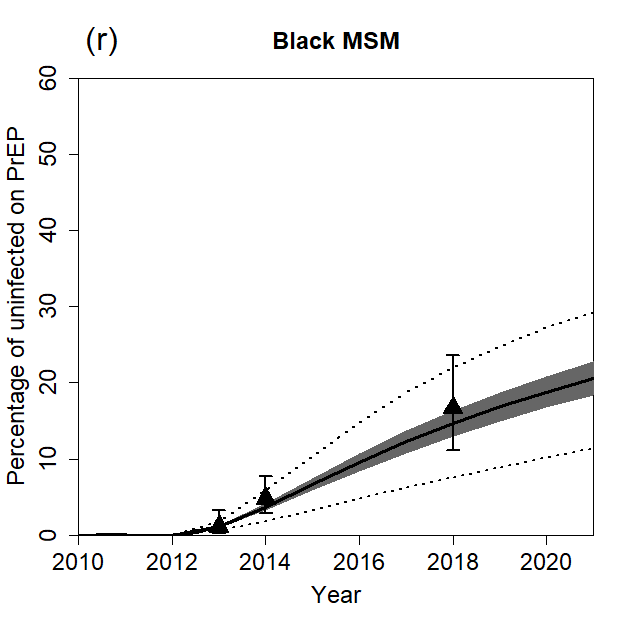


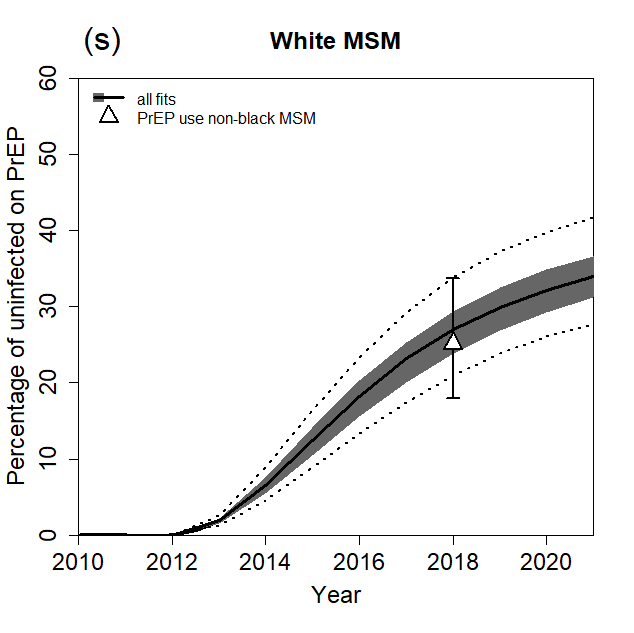


**Figure S4.** **Model fits to demography, care cascade and PrEP data for men who have sex with men (MSM) in Atlanta.** (a) Percentage of all MSM aged 18-24 years old, (b) percentage of MSM who are Black, (c-f) percentage of all MSM with a PrEP indication, for age- and race-groups indicated, (g) percentage of HIV-positive MSM who are aware of their HIV-positive status, (h) percentage of all diagnosed HIV-positive MSM who are linked into care, (i) percentage of Black diagnosed HIV-positive MSM who are linked into care, (j) percentage of White diagnosed HIV-positive MSM who are linked into care, (k) percentage of all diagnosed HIV-positive MSM who are virally suppressed, (l) percentage of Black diagnosed HIV-positive MSM who are virally suppressed, (m) percentage of White diagnosed HIV-positive MSM who are virally suppressed, (n) percentage of MSM on ART who are virally suppressed, (o-s) percentage of HIV-negative MSM who are taking PrEP, for age- and race-groups indicated . Results are for all 114 fitting parameter combinations. Results show median (thick lines), 25th to 75th percentile (dark shaded area), and 2.5th and 97.5th percentiles (dotted lines) across model fits. Points and error bars show the mean and 95% CI for NHBS data for Atlanta (a-f), mean and 95% CI for CDC state-level data (g), mean and ±10 percentage points for DPH state-level continuum data (h-m), mean and range for national data (n), and mean and 95%CI for PrEP use estimates from various sources (o-s). Black filled points used for model fitting, white points for model validation.

**Figure S5. Sensitivity analysis on assumptions for TDF/FTC adherence.** PrEP impact on new HIV infections over five years (2022-2026) for different PrEP interventions shown, using TDF/FTC adherence from the PrEP Demo project (white bars) or the HPTN 083 trial (striped bars). (a) Impact calculated in comparison with no PrEP base case scenario, (b) impact of CAB calculated in comparison with continued TDF/FTC use scenario (“incremental impact”). Only MSM with a PrEP indication use PrEP. Bars and error bars show median and 95% credible interval across 114 fits.

#

# References

1. Grey JA, Bernstein KT, Sullivan PS, et al. Estimating the population sizes of men who have sex with men in US states and counties using data from the American community survey. *JMIR Public Health Surveill* 2016; **2**(1): e14.

2. Purcell DW, Johnson CH, Lansky A, et al. Estimating the population size of men who have sex with men in the United States to obtain HIV and syphilis rates. *Open AIDS J* 2012; **6**: 98-107.

3. Centers for Disease Control and Prevention: US Public Health Service. Preexposure prophylaxis for the prevention of HIV infection in the United States—2017 Update: a clinical practice guideline. , 2018.

4. Hoots BE, Finlayson T, Nerlander L, Paz-Bailey G. Willingness to take, use of, and indications for pre-exposure prophylaxis among men who have sex with men-20 US cities, 2014. *Clin Infect Dis* 2016; **63**(5): 672-7.

5. CDC. WONDER online database. <https://wonder.cdc.gov/>.

6. Kalichman SC, Eaton L, Cain D, et al. Changes in HIV treatment beliefs and sexual risk behaviors among gay and bisexual men, 1997-2005. *Health Psychol* 2007; **26**(5): 650-6.

7. Sullivan PS, Salazar L, Buchbinder S, Sanchez TH. Estimating the proportion of HIV transmissions from main sex partners among men who have sex with men in five US cities. *AIDS* 2009; **23**(9): 1153-62.

8. Delaney KP, Rosenberg ES, Kramer MR, Waller LA, Sullivan PS. Optimizing human immunodeficiency virus testing interventions for men who have sex with men in the United States: a modeling study. *Open Forum Infect Dis* 2015; **2**(4): ofv153.

9. Mitchell JW, Petroll AE. Patterns of HIV and sexually transmitted infection testing among men who have sex with men couples in the United States. *Sex Transm Dis* 2012; **39**(11): 871-6.

10. Silhol R, Boily M-C, Dimitrov D, et al. Understanding the HIV epidemic among MSM in Baltimore: A modeling study estimating the impact of past HIV interventions and who acquired and contributed to infections. *J Acquir Immune Defic Syndr* 2020; **84**(3): 253-62.

11. Hollingsworth TD, Anderson RM, Fraser C. HIV-1 transmission, by stage of infection. *J Infect Dis* 2008; **198**(5): 687-93.

12. Boily MC, Baggaley RF, Wang L, et al. Heterosexual risk of HIV-1 infection per sexual act: systematic review and meta-analysis of observational studies. *Lancet Infect Dis* 2009; **9**(2): 118-29.

13. Dunn D, Woodburn P, Duong T, et al. Current CD4 cell count and the short-term risk of AIDS and death before the availability of effective antiretroviral therapy in HIV-infected children and adults. *J Infect Dis* 2008; **197**(3): 398-404.

14. Cori A, Pickles M, van Sighem A, et al. CD4+ cell dynamics in untreated HIV-1 infection: overall rates, and effects of age, viral load, sex and calendar time. *AIDS* 2015; **29**(18): 2435-46.

15. Egger M, May M, Chene G, et al. Prognosis of HIV-1-infected patients starting highly active antiretroviral therapy: a collaborative analysis of prospective studies. *Lancet* 2002; **360**(9327): 119-29.

16. Antiretroviral Therapy Cohort Collaboration. Importance of baseline prognostic factors with increasing time since initiation of highly active antiretroviral therapy: collaborative analysis of cohorts of HIV-1-infected patients. *J Acquir Immune Defic Syndr* 2007; **46**(5): 607-15.

17. Mellors JW, Munoz A, Giorgi JV, et al. Plasma viral load and CD4+ lymphocytes as prognostic markers of HIV-1 infection. *Ann Intern Med* 1997; **126**(12): 946-54.

18. Herbeck JT, Gottlieb GS, Li X, et al. Lack of evidence for changing virulence of HIV-1 in North America. *PLoS One* 2008; **3**(2): e1525.

19. Mitchell KM, Hoots B, Dimitrov D, et al. Improvements in the HIV care continuum needed to meaningfully reduce HIV incidence among men who have sex with men in Baltimore, US: a modelling study for HPTN 078. *J Int AIDS Soc* 2019; **22**(3): e25246.

20. Donnell D, Baeten JM, Kiarie J, et al. Heterosexual HIV-1 transmission after initiation of antiretroviral therapy: a prospective cohort analysis. *Lancet* 2010; **375**(9731): 2092-8.

21. Baggaley RF, White RG, Boily MC. HIV transmission risk through anal intercourse: systematic review, meta-analysis and implications for HIV prevention. *Int J Epidemiol* 2010; **39**(4): 1048-63.

22. Jin F, Jansson J, Law M, et al. Per-contact probability of HIV transmission in homosexual men in Sydney in the era of HAART. *AIDS* 2010; **24**(6): 907-13.

23. Blaser N, Wettstein C, Estill J, et al. Impact of viral load and the duration of primary infection on HIV transmission: systematic review and meta-analysis. *AIDS* 2014; **28**(7): 1021-9.

24. Smith DK, Herbst JH, Zhang X, Rose CE. Condom effectiveness for HIV prevention by consistency of use among men who have sex with men in the United States. *J Acquir Immune Defic Syndr* 2015; **68**(3): 337-44.

25. Mills E, Cooper C, Anema A, Guyatt G. Male circumcision for the prevention of heterosexually acquired HIV infection: a meta-analysis of randomized trials involving 11,050 men. *HIV Med* 2008; **9**(6): 332-5.

26. Rodger AJ, Cambiano V, Bruun T, et al. Sexual activity without condoms and risk of HIV transmission in serodifferent couples when the HIV-positive partner is using suppressive antiretroviral therapy. *JAMA* 2016; **316**(2): 171-81.

27. Anderson PL, Glidden DV, Liu A, et al. Emtricitabine-tenofovir concentrations and pre-exposure prophylaxis efficacy in men who have sex with men. *Sci Transl Med* 2012; **4**(151): 151ra25.

28. Hanscom B, Janes HE, Guarino PD, et al. Preventing HIV-1 infection in women using oral preexposure prophylaxis: A meta-analysis of current evidence. *J Acquir Immune Defic Syndr* 2016; **73**(5): 606-8.

29. Hanscom B, Hughes JP, Williamson BD, Donnell D. Adaptive non-inferiority margins under observable non-constancy. *Stat Methods Med Res* 2019; **28**(10-11): 3318-32.

30. Anderson JE, Carey JW, Taveras S. HIV testing among the general US population and persons at increased risk: information from national surveys, 1987-1996. *Am J Public Health* 2000; **90**(7): 1089-95.

31. Georgia Department of Public Health. HIV/AIDS Epidemiology Program HIV Care Continuum Surveillance Report, Georgia, 2012, 2014.

32. Georgia Department of Public Health. HIV Care Continuum Report, Georgia, 2014, 2016.

33. Centers for Disease Control and Prevention. Reported CD4+ T-lymphocyte results for adults and adolescents with HIV/AIDS - 33 states, 2005. *HIV/AIDS Surveillance Report* 2005; **11**(2).

34. Centers for Disease Control and Prevention. Reported CD4+ T-lymphocyte and viral load results for adults and adolesecents with HIV infection - 37 states, 2005-2007. *HIV Surveillance Supplemental Report* 2010; **16**(1).

35. Moore RD, Keruly JC, Bartlett JG. Improvement in the health of HIV-infected persons in care: reducing disparities. *Clin Infect Dis* 2012; **55**(9): 1242-51.

36. Rebeiro P, Althoff KN, Buchacz K, et al. Retention among North American HIV-infected persons in clinical care, 2000-2008. *J Acquir Immune Defic Syndr* 2013; **62**(3): 356-62.

37. Tedaldi EM, Richardson JT, Debes R, et al. Retention in care within 1 year of initial HIV care visit in a multisite US cohort: who's in and who's out? *J Int Assoc Provid AIDS Care* 2014; **13**(3): 232-41.

38. Li X, Margolick JB, Conover CS, et al. Interruption and discontinuation of highly active antiretroviral therapy in the multicenter AIDS cohort study. *J Acquir Immune Defic Syndr* 2005; **38**(3): 320-8.

39. Howe CJ, Cole SR, Napravnik S, Eron JJ. Enrollment, retention, and visit attendance in the University of North Carolina Center for AIDS Research HIV clinical cohort, 2001-2007. *AIDS Res Hum Retroviruses* 2010; **26**(8): 875-81.

40. Dasgupta S, Oster AM, Li J, Hall HI. Disparities in consistent retention in HIV care - 11 states and the district of Columbia, 2011-2013. *MMWR Morb Mortal Wkly Rep* 2016; **65**(4): 77-82.

41. Mocroft A, Furrer HJ, Miro JM, et al. The incidence of AIDS-defining illnesses at a current CD4 count >/= 200 cells/muL in the post-combination antiretroviral therapy era. *Clin Infect Dis* 2013; **57**(7): 1038-47.

42. Guiguet M, Porter K, Phillips A, Costagliola D, Babiker A. Clinical progression rates by CD4 cell category before and after the initiation of combination antiretroviral therapy (cART). *Open AIDS J* 2008; **2**: 3-9.

43. Singh S, Bradley H, Hu X, Skarbinski J, Hall HI, Lansky A. Men living with diagnosed HIV who have sex with men: progress along the continuum of HIV care--United States, 2010. *MMWR Morb Mortal Wkly Rep* 2014; **63**(38): 829-33.

44. Althoff KN, Rebeiro P, Brooks JT, et al. Disparities in the quality of HIV care when using US Department of Health and Human Services indicators. *Clin Infect Dis* 2014; **58**(8): 1185-9.

45. Novak RM, Hart RL, Chmiel JS, Brooks JT, Buchacz K. Disparities in initiation of combination antiretroviral treatment and in virologic suppression among patients in the HIV Outpatient Study (HOPS), 2000-2013. *J Acquir Immune Defic Syndr* 2015.

46. Weintrob AC, Grandits GA, Agan BK, et al. Virologic response differences between African Americans and European Americans initiating highly active antiretroviral therapy with equal access to care. *J Acquir Immune Defic Syndr* 2009; **52**(5): 574-80.

47. Robertson M, Laraque F, Mavronicolas H, Braunstein S, Torian L. Linkage and retention in care and the time to HIV viral suppression and viral rebound - New York City. *AIDS Care* 2015; **27**(2): 260-7.

48. Krishnan S, Wu K, Smurzynski M, et al. Incidence rate of and factors associated with loss to follow-up in a longitudinal cohort of antiretroviral-treated HIV-infected persons: an AIDS Clinical Trials Group (ACTG) Longitudinal Linked Randomized Trials (ALLRT) analysis. *HIV Clin Trials* 2011; **12**(4): 190-200.

49. Grinsztejn B. HPTN 083: Efficacy of pre-exposure prophylaxis (PrEP) containing long-acting injectable cabotegravir (CAB-LA) is maintained across regions and key populations. 23rd International AIDS Conference. virtual; 2020.

50. UK Office for National Statistics. Deaths: age sex. England and Wales [table 6.1]. *Popul Trends* 2006; **126**: 49.

51. Michigan Department of Community Health. Adult and Adolescent Spectrum of Disease Project in Michigan Summary Report 1990-2003.

52. Drake AL, Kinuthia J, Matemo D, et al. Virologic and immunologic response following antiretroviral therapy initiation among pregnant and postpartum women with acute HIV-1 infection [abstract #MOPDB0101]. International AIDS conference. Melbourne, Australia; 2014.

53. Donnell D, Baeten JM, Kiarie J, et al. Heterosexual HIV-1 transmission after initiation of antiretroviral therapy: a prospective cohort analysis. *Lancet* 2010; **375**(9731): 2092-8.

54. Hallett TB, Baeten JM, Heffron R, et al. Optimal uses of antiretrovirals for prevention in HIV-1 serodiscordant heterosexual couples in South Africa: a modelling study. *PLoS Med* 2011; **8**(11): e1001123.

55. Georgia Department of Public Health. HIV/AIDS epidemiology program HIV care continuum surveillance report, Georgia, 2011, 2013.

56. Hoots BE, Finlayson TJ, Wejnert C, Paz-Bailey G. Early linkage to HIV care and antiretroviral treatment among men who have sex with men - 20 cities, United States, 2008 and 2011. *PLoS One* 2015; **10**(7): e0132962.

57. Cohen MS, Chen YQ, McCauley M, et al. Antiretroviral therapy for the prevention of HIV-1 transmission. *N Engl J Med* 2016; **375**(9): 830-9.

58. Landovitz RJ, Donnell D, Clement ME, et al. Cabotegravir for HIV prevention in cisgender men and transgender women. *N Engl J Med* 2021; **385**(7): 595-608.

59. Palella FJ, Jr., Delaney KM, Moorman AC, et al. Declining morbidity and mortality among patients with advanced human immunodeficiency virus infection. HIV Outpatient Study Investigators. *N Engl J Med* 1998; **338**(13): 853-60.

60. Georgia Department of Public Health. HIV behavioral surveillance data summary: men who have sex with men (MSM) in Metro Atlanta, 2017, 2019.

61. Hall HI, An Q, Tang T, et al. Prevalence of diagnosed and undiagnosed HIV infection - United States, 2008-2012. *MMWR Morb Mortal Wkly Rep* 2015; **64**(24): 657-62.

62. Georgia Department of Public Health. Georgia HIV care continuum update: Persons living with HIV, 2016, and persons diagnosed with HIV, 2015 [slides], 2017.

63. Georgia Department of Public Health. Georgia HIV care continuum update: persons living with HIV and persons diagnosed with HIV, 2017 [slides], 2019.

64. Georgia Department of Public Health. Georgia HIV care continuum update: persons living with HIV, and persons diagnosed with HIV, 2018 [slides], 2020.

65. Georgia Department of Public Health. Georgia HIV care continuum update: Persons living with HIV, and persons diagnosed with HIV, 2019 [slides], 2021.

66. Hall HI, Frazier EL, Rhodes P, et al. Differences in human immunodeficiency virus care and treatment among subpopulations in the United States. *JAMA Inter Med* 2013; **173**(14): 1337-44.

67. Chapin-Bardales J, Martin A, Haaland R, et al. Factors associated with PrEP persistence and adherence among MSM in 4 U.S. cities [abstract number 0991]. Conference on Retroviruses and Opportunistic Infections (CROI). Boston, Massachusetts; 2020.

68. Goedel WC, Halkitis PN, Greene RE, Hickson DA, Duncan DT. HIV risk behaviors, perceptions, and testing and preexposure prophylaxis (PrEP) awareness/use in Grindr-using men who have sex with men in Atlanta, Georgia. *J Assoc Nurses AIDS Care* 2016; **27**(2): 133-42.

69. Centers for Disease Control and Prevention. HIV infection risk, prevention, and testing behaviors among men who have sex with men—national HIV behavioral surveillance, 23 U.S. cities, 2017. *HIV Surveillance Special Report 8* 2019; **22**.

70. Onwubiko U, Holland D, Ajoku S, et al. Using PrEP to #STOPHIVATL: Findings from a cross-sectional survey among gay men and transgender women participating in gay pride events in Atlanta, Georgia, 2018. *Arch Sex Behav* 2020; **49**(6): 2193-204.

71. Eaton LA, Driffin DD, Bauermeister J, Smith H, Conway-Washington C. Minimal awareness and stalled uptake of pre-exposure prophylaxis (PrEP) among at risk, HIV-negative, Black men who have sex with men. *AIDS Patient Care STDS* 2015; **29**(8): 423-9.

72. Eaton LA, Matthews DD, Driffin DD, Bukowski L, Wilson PA, Stall RD. A multi-US city assessment of awareness and uptake of pre-exposure prophylaxis (PrEP) for HIV prevention among Black men and transgender women who have sex with men. *Prev Sci* 2017; **18**(5): 505-16.

73. Rolle C-P, Rosenberg ES, Siegler AJ, et al. Challenges in translating PrEP interest into uptake in an observational study of young Black MSM. *J Acquir Immune Defic Syndr* 2017; **76**(3): 250-8.
