# Supplementary material for: Estimating the impact of HIV PrEP regimens containing long-acting injectable cabotegravir or daily oral tenofovir disoproxil fumarate/emtricitabine among men who have sex with men in the United States: a mathematical modelling study for HPTN 083
Source: Lancet Reg Health Am. 2023 Jan 17;18:100416. doi: 10.1016/j.lana.2022.100416 (PMC9950652; doi:10.1016/j.lana.2022.100416)
Supplement: Caption for Supplementary Material [file mmc2.docx]

**Supplementary material: Supplemental methods, including model structure, model equations, model parameters and fitting data, and supplemental results.**
